# Metathesis of a U^V^ imido complex: a route to a terminal U^V^ sulfide†
†Electronic supplementary information (ESI) available: Full computational and experimental details, NMR spectra, and detailed X-ray crystallographic data in CIF format. CCDC 1535285–1535289. For ESI and crystallographic data in CIF or other electronic format see DOI: 10.1039/c7sc01111c



**DOI:** 10.1039/c7sc01111c

**Published:** 2017-06-05

**Authors:** Rory P. Kelly, Marta Falcone, Carlos Alvarez Lamsfus, Rosario Scopelliti, Laurent Maron, Karsten Meyer, Marinella Mazzanti

**Affiliations:** a Institut des Sciences et Ingénierie Chimiques , Ecole Polytechnique Fédérale de Lausanne (EPFL) , 1015 Lausanne , Switzerland . Email: marinella.mazzanti@epfl.ch; b Université de Toulouse et CNRS INSA , UPS , CNRS , UMR 5215 , LPCNO , 135 avenue de Rangueil , 31077 Toulouse , France; c Department of Chemistry and Pharmacy , Inorganic Chemistry , Friedrich-Alexander University Erlangen-Nürnberg , Egerlandstraße 1 , 91058 Erlangen , Germany

## Abstract

Herein, we report the synthesis and characterisation of the first terminal uranium(v) sulfide and a related U^V^ trithiocarbonate complex supported by sterically demanding tris(*tert*-butoxy)siloxide ligands. The reaction of the potassium-bound U^V^ imido complex, [U(NAd){OSi(O*t*Bu)_3_}_4_K] (**4**), with CS_2_ led to the isolation of perthiodicarbonate [K(18c6)]_2_[C_2_S_6_] (**6**), with concomitant formation of the U^IV^ complex, [U{OSi(O*t*Bu)_3_}_4_], and S=C=NAd. In contrast, the reaction of the U^V^ imido complex, [K(2.2.2-cryptand)][U(NAd){OSi(O*t*Bu)_3_}_4_] (**5**), with one or two equivalents of CS_2_ afforded the trithiocarbonate complex, [K(2.2.2-cryptand)][U(CS_3_){OSi(O*t*Bu)_3_}_4_] (**7**), which was isolated in 57% yield, with concomitant elimination of the admantyl thiocyanate product, S=C=NAd. Complex **7** is likely formed by fast nucleophilic addition of a U^V^ terminal sulfide intermediate, resulting from the slow metathesis reaction of the imido complex with CS_2_, to a second CS_2_ molecule. The addition of a solution of H_2_S in thf (1.3 eq.) to **4** afforded the first isolable U^V^ terminal sulfide complex, [K(2.2.2-cryptand)][US{OSi(O*t*Bu)_3_}_4_] (**8**), in 41% yield. Based on DFT calculations, triple-bond character with a strong covalent interaction is suggested for the U–S bond in complex **7**.

## Abstract 2

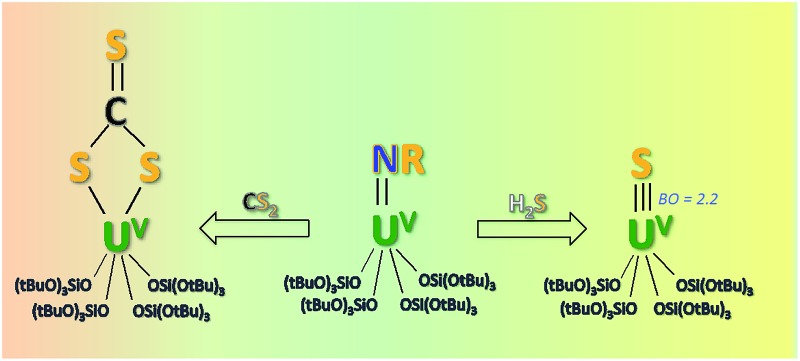
The metathesis reaction of a U^V^ imido complex supported by sterically demanding tris(*tert*-butoxy)siloxide ligands with CS_2_ afforded a terminal U^V^ thiocarbonate but metathesis with H_2_S afforded the first example of a terminal U^V^ sulfide.

## Introduction

Interest in multiply-bonded uranium pnictogen and chalcogen compounds has grown considerably in recent years.^1^ The study of actinide–chalcogen bonds is in part motivated by the efficiency of chalcogen donors in the selective separation of actinides from lanthanides in spent nuclear fuel, a property that has been related to covalent contributions in actinide–chalcogen bonds.^2^ Early attempts to prepare terminal sulfido, selenido and tellurido complexes of uranium involved oxidation of a U^III^ precursor with a chalcogen-atom donor, and led exclusively to chalcogenide-bridged compounds.^3a–g^ In recent years, a handful of terminal and alkali-capped mononuclear uranium chalcogenides have been prepared and characterised.^4^ All characterised terminal sulfido, selenido and tellurido complexes contain a tetravalent uranium ion.^4a,c–e,5^ Only one U^VI^ complex containing a linear O=U=S^2+^ core has been characterised by Hayton and co-workers.^6^ Several terminal mono–oxo complexes of pentavalent uranium^7–9^ and a few capped^8d,10^ and terminal U^V^ nitrides^8d,10a,11^ have been prepared in recent years, but terminal sulfido, selenido and tellurido complexes of U^V^ remain undiscovered. Since the degree of covalency in the uranium–chalcogenide bond is expected to be higher in higher oxidation states,^1*a*^ the isolation of a U^V^ terminal sulfide is of great interest for elucidating the involvement of 5f orbitals in U–S bonding. In general, pentavalent uranium compounds are attractive candidates for the investigation of bonding and magnetic properties due to their simple 5f^1^ configuration,^12^ but the number of molecular uranium compounds containing a U^V^–S bond remain rare.^3g,12b,13^ The presence of stable U^V^ cations in chalcogenide materials has also been reported.^14^

Different approaches have been used in order to prevent the formation of bridging species when preparing U^IV^ mono-chalcogenide complexes by oxidation of U^III^ compounds.^4b,c^ Recently, our group used sterically demanding tris(*tert*-butoxy)siloxide ligands to prevent the formation of a bridging chalcogenide complex. The reaction of the ate complex, [U(OSi(O*t*Bu)_3_)_4_K], with the two-electron oxidising agent, Ph_3_PS, in the presence of 2.2.2-cryptand, led to the isolation of the terminal uranium(iv) monosulfide complex, [K(2.2.2-cryptand)][US(OSi(O*t*Bu)_3_)_4_K].^4*e*^ We note that all of the examples mentioned above resulted in the formation of a U^IV^ mono-chalcogenide complex in spite of the fact that a two-electron oxidising agent was used in the sulfur–transfer reactions to U^III^. This suggests that the isolation of terminal sulfides of U^V^ or U^VI^ from U^III^ might not be possible. A monosulfido complex of U^IV^ was also prepared by deprotonation of a hydrosulfido analogue, [((^Ad,Me^ArO)_3_tacn)U–SH] supported by a tripodal hexadentate aminophenolate ligand.^4*d*^ The reported electrochemical studies indicated that this complex could be electrochemically oxidised, most likely to the U^V^ ≡S species, but preliminary attempts to chemically oxidise and isolate a U^V^ sulfido complex were not successful.

**Fig. 1:**
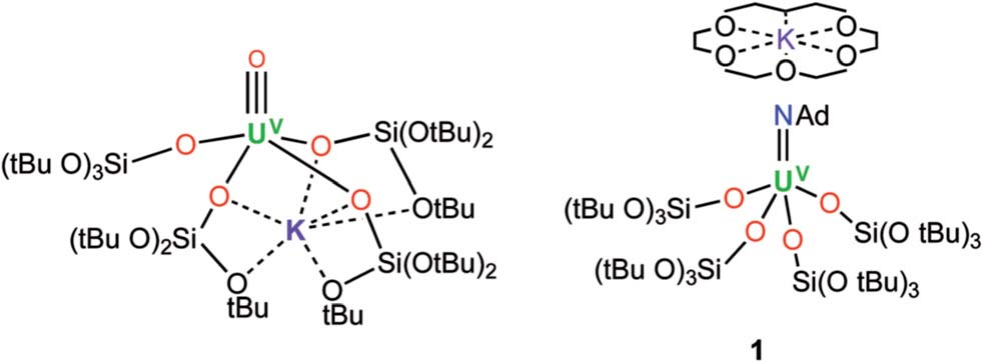
Molecular structures of the U^V^ terminal oxo complex, [UO{OSi(O*t*Bu)_3_}_4_K] (left), and the U^V^ terminal imido complex, [K(18c6)][U(NAd){OSi(O*t*Bu)_3_}_4_] (**1**) (right).

Here we investigate new possible routes to isolate a U^V^ terminal sulfide using tris(*tert*-butoxy)siloxide as the supporting ligands. This ligand previously allowed for the isolation and characterisation of the U^V^ terminal oxo complex, [UO(OSi(O*t*Bu)_3_)_4_K],^8*b*^ and of the U^V^ terminal imido complex, [K(18c6)][U(NAd)(OSi(O*t*Bu)_3_)_4_] (**1**)^15^ (Ad = adamantyl) (see Fig. 1). Herein, we show that metathesis reactions of U^V^ tetrasiloxide imido complexes with CS_2_ and H_2_S afford the first U^V^ terminal sulfide and trithiocarbonate complexes.

## Results and discussion

### Syntheses and molecular structures

Attempts to isolate a U^V^ terminal sulfide from the chemical oxidation of the uranium(iv) siloxide complex, [K(2.2.2-cryptand)][US(OSi(O*t*Bu)_3_)_4_K], only led to decomposition products.

Thus, in our search for a U^V^ terminal sulfide, we anticipated that U^V^ terminal imido complexes would be the ideal starting materials. Notably, several examples of reactions of transition metal imido compounds with CS_2_ have been reported and they usually lead to the formation of sulfide and isothiocyanate products *via* a cycloaddition pathway.^16^ The formation of a U^V^ terminal oxo complex from the reaction of a U^V^ imido complex with CO_2_ has been reported,^8*a*^ but a similar strategy using CS_2_ has never been used to prepare terminal uranium sulfides. Hydrosulfidolysis of imido compounds also represents a successful route to terminal or bridging sulfide complexes of d-block transition metals,^17^ but it has never been applied for f-elements.

We have previously shown that [K(18c6)][U{OSi(O*t*Bu)_3_}_4_] can be used to effect a two-electron reduction of adamantyl azide, affording the U^V^ monoimido complex [K(18c6)][U(NAd){OSi(O*t*Bu)_3_}_4_] (**1**).^15^ With regard to the important effect of alkali cations and crown-ether-bound alkali cations on the reactivity of uranium compounds supported by tris(*tert*-butoxy)siloxide ligands,^8b,18^ we have now prepared the analogous complexes, [U(NAd){OSi(O*t*Bu)_3_}_4_K] (**4**), and [K(2.2.2-cryptand)][U(NAd){OSi(O*t*Bu)_3_}_4_] (**5**), by reduction of adamantyl azide with [U{OSi(O*t*Bu)_3_}_4_K] (**2**) and [K(2.2.2-cryptand)][U{OSi(O*t*Bu)_3_}_4_] (**3**), respectively.

**Fig. 2:**
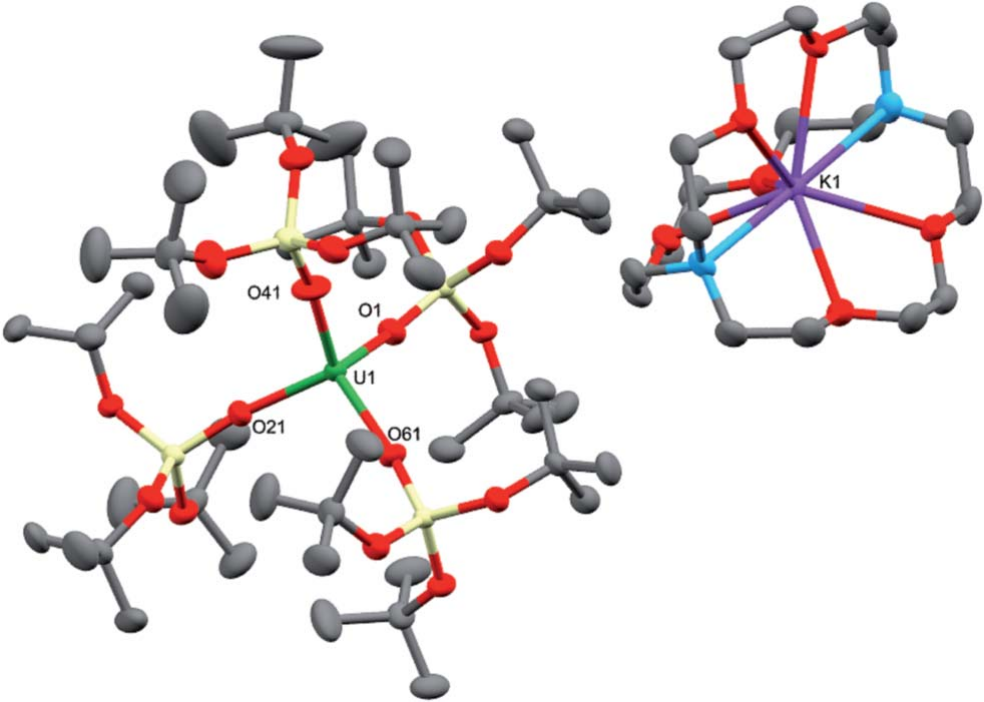
Molecular structure of one of the crystallographically unique pairs of [K(2.2.2-cryptand)][U{OSi(O*t*Bu)_3_}_4_] in crystals of **3** shown with 50% probability thermal ellipsoids. Hydrogen atoms have been omitted for clarity. Selected bond lengths (Å): U1–O1 = 2.223(4), U1–O21 = 2.193(4), U1–O41 = 2.211(4), U1–O61 = 2.220(4).

The charge-separated U^III^ tetrasiloxide precursor, [K(2.2.2-cryptand)][U{OSi(O*t*Bu)_3_}_4_] (**3**), is conveniently prepared in high yield by stirring the reported complex, [U{OSi(O*t*Bu)_3_}_4_K] (**2**),^8*b*^ with 2.2.2-cryptand in toluene. Complex **3** crystallised from a mixture of thf and hexane as two crystallographically unique pairs of [K(2.2.2-cryptand)]^+^ and [U{OSi(O*t*Bu)_3_}_4_]^–^ ions in the orthorhombic space group, *P*2_1_2_1_2_1_. The molecular structure is shown in Fig. 2. The four-coordinate uranium ions feature a tetrahedral coordination geometry formed by four monodentate tris(*tert*-butoxy)siloxide ligands. The structure is very similar to that of [K(18c6)][U(NAd){OSi(O*t*Bu)_3_}_4_] and the U–O bond lengths of the two complexes are comparable (U–O_ave_ = 2.228 Å in [K(18c6)][U(NAd){OSi(O*t*Bu)_3_}_4_]; U–O_ave_ = 2.21 Å in **3**).

**Scheme 1:**
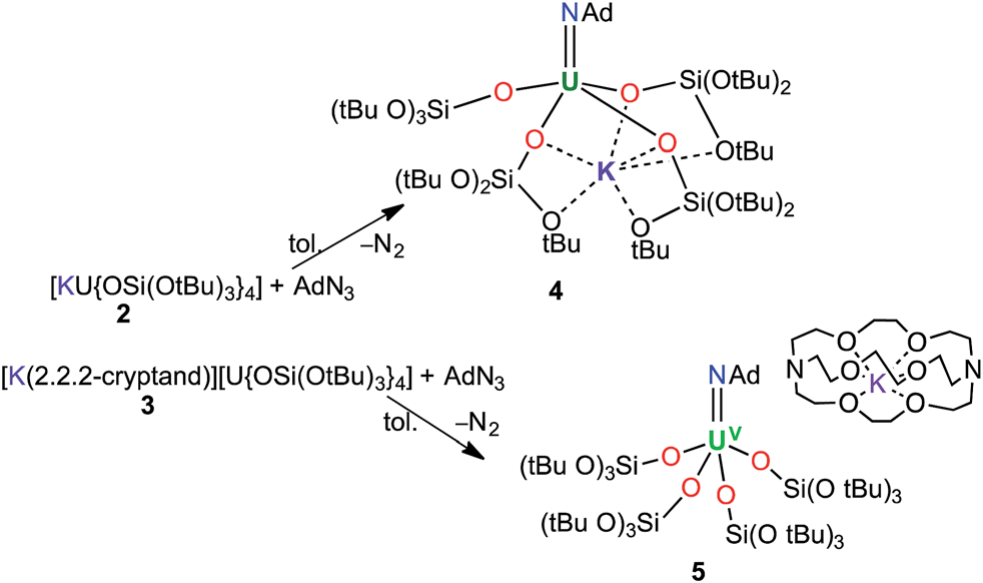
Syntheses of [U(NAd){OSi(O*t*Bu)_3_}_4_K] (**4**) and [K(2.2.2-cryptand)][U(NAd){OSi(O*t*Bu)_3_}_4_] (**5**).

Treating complexes **2** and **3** with adamantyl azide in toluene yielded the U^V^ imido complexes, [U(NAd){OSi(O*t*Bu)_3_}_4_K] (**4**), and [K(2.2.2-cryptand)][U(NAd){OSi(O*t*Bu)_3_}_4_] (**5**), respectively (Scheme 1). Complex **4** is highly soluble in hexane, toluene and thf, whereas **5** is sparingly soluble in toluene but highly soluble in thf. The ^1^H NMR spectra of **4** and **5** in d_8_-toluene are similar to that of the reported complex **1**,^15^ and show four paramagnetically shifted resonances attributable to the adamantyl protons, and one peak corresponding to the *tert*-butyl protons of the siloxide ligands. However, in the case of **5** the siloxide peak is sharp, while in the case of **4** a broad peak is observed, suggesting fluxional binding of the potassium ion in toluene solution for complex **4**. Complex **5** shows three additional cryptand resonances in the ^1^H NMR spectrum.

**Fig. 3:**
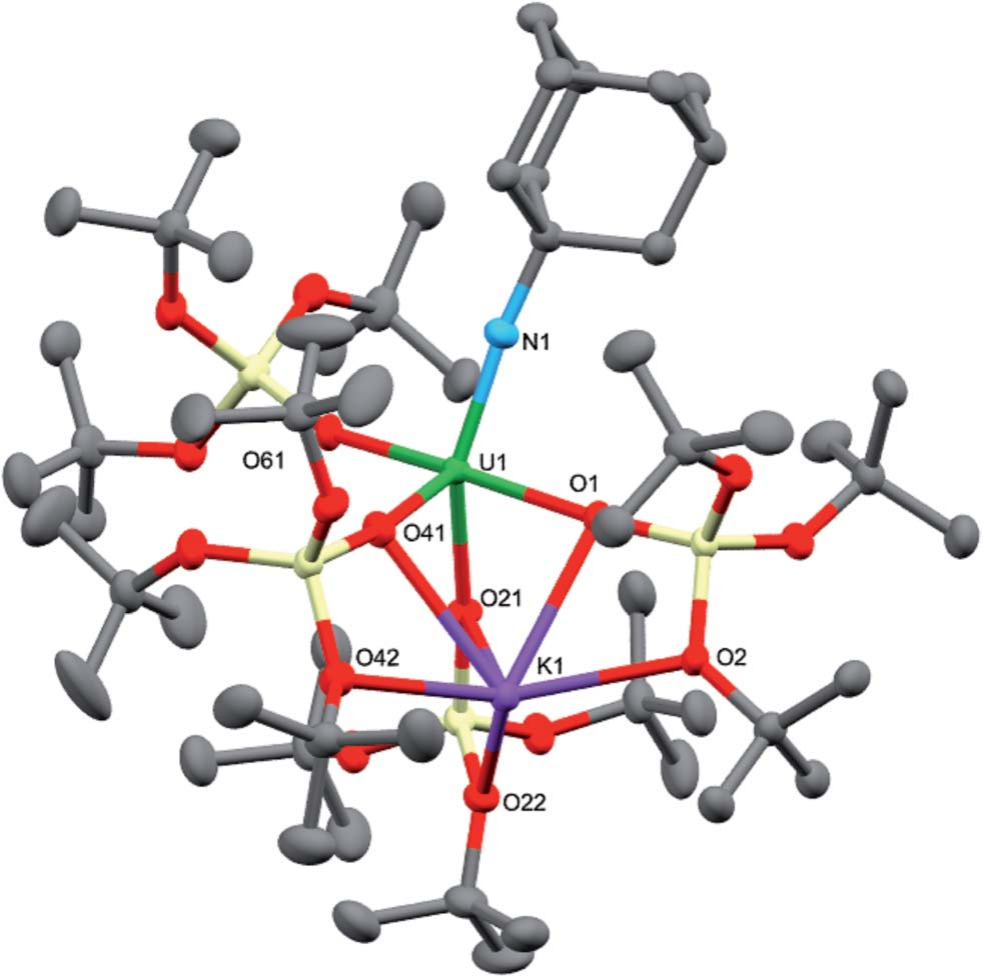
Molecular structure of [U(NAd){OSi(O*t*Bu)_3_}_4_K] in crystal of **4**·tol shown with 50% probability thermal ellipsoids. Hydrogen atoms and lattice solvent have been omitted for clarity. Selected bond lengths (Å): U1–N1 = 1.954(3), U1–O1 = 2.243(3), U1–O21 = 2.175(3), U1–O41 = 2.190(3), U1–O61 = 2.185(3), K1–O1 = 2.827(3), K1–O2 = 2.631(3), K1–O21 = 3.058(3), K1–O22 = 2.626(3), K1–O41 = 2.822(4), K1–O42 = 2.725(4).

Dark brown crystals of the heterobimetallic complex **4**·tol crystallised from toluene in the orthorhombic space group, *Fdd*2. The molecular structure is shown in Fig. 3. The central uranium ion is five coordinate and it is ligated by four negatively charged oxygen atoms of the tris(*tert*-butoxy)siloxide ligands, and one nitrogen atom of the imido group. The U–N bond length (1.954(3) Å) is slightly longer than the corresponding bond length in [K(18c6)][U(NAd){OSi(O*t*Bu)_3_}_4_] (1.937(7) Å), while the average U–O bond lengths (2.20(2) Å for [K(18c6)][U(NAd){OSi(O*t*Bu)_3_}_4_] and 2.20(3) Å for **4**) of the two complexes are about the same.^15^ The incorporation of the potassium ion into the structure of **4** results in significant distortion of the coordination geometry around the uranium ion relative to that found in **1**. In **1**, the coordination geometry of the uranium centre is roughly trigonal bipyramidal, with three siloxide ligands occupying the equatorial sites, and the axial sites being taken up by a siloxide ligand and an imido group, respectively. However, in **4**, the coordination geometry around the uranium ion is highly distorted due to the coordination of three siloxide ligands to the six-coordinate potassium ion, which fits into a pocket formed by three κ^2^O–siloxide ligands.

At first, we investigated the reaction of [U(NAd){OSi(O*t*Bu)_3_}_4_K] (**4**) with ^13^CS_2_. An analogous approach has been used to prepare a uranium terminal oxo complex by reaction of a U^V^ imido complex with CO_2_.^8*a*^ The proposed mechanism for the formation of the terminal oxo involves a [2 + 2] cycloaddition reaction followed by extrusion of isocyanate to afford the terminal oxo complex.^8*a*^

**Scheme 2:**
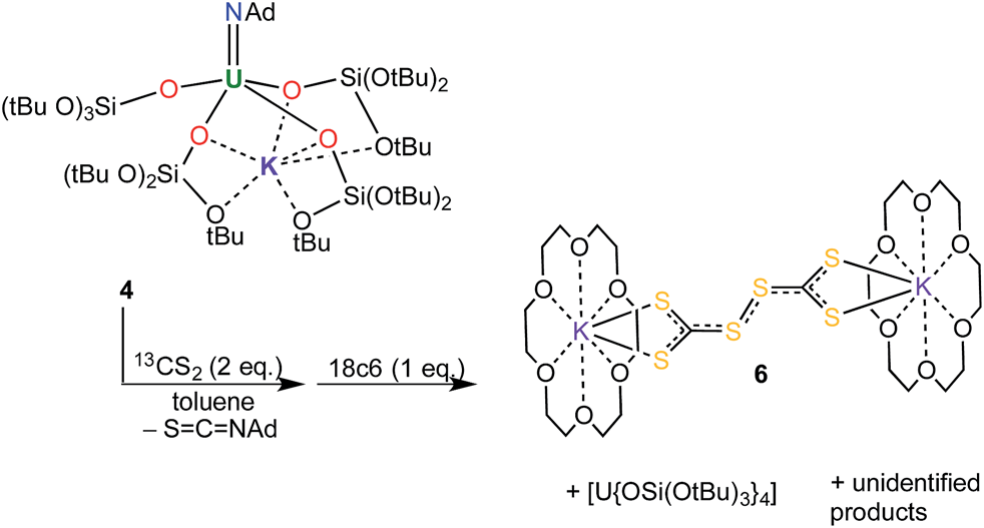
Reaction of **4** with CS_2_: isolation of **6**.

In the present case, reactions between **4** and one or two equivalents of ^13^CS_2_ were slow. Monitoring the reactions by ^1^H NMR spectroscopy showed that in both cases consumption of the starting material took place over two to three days, and it proceeded with the concomitant formation of [U{OSi(O*t*Bu)_3_}_4_] (in 35% yield) and additional unidentified uranium product(s). None of these products could be identified as a terminal sulfide, even when the **4** : CS_2_ ratio was 1 : 1. The ^13^C NMR spectra of the crude reaction mixtures in d_8_-toluene show the presence of the metathesis by-product, the isothiocyanate S=C=NAd, in both cases, as well as an additional signal at 132 ppm assigned to the perthiodicarbonate C_2_S_6_^2–^. The formation of an insoluble compound is also observed. The ^13^C NMR spectrum in d_6_-dmso of the residue obtained after removal of toluene form the reaction mixture shows the presence of peaks at 267 ppm and 129 ppm assigned to the CS_3_^2–^ and to the C_2_S_6_^2–^ species in a ratio of 1 : 1.2. Adding 18c6 to a 1 : 2 toluene reaction mixture of **4** and CS_2_ allowed for the crystallisation of the unusual CS_3_-coupling product, [K(18c6)]_2_[C_2_S_6_] (**6**) (Scheme 2). The molecular structure of **6** was determined by X-ray crystallography (see ESI†). Perthiodicarbonate species are rare but some examples are known, *e.g.* [PPh_4_]_2_[C_2_S_6_], which formed from aerial oxidation of a reaction mixture of PPh_4_Cl and K_2_(CS_3_).^19^

Complex **6** plausibly arises from uranium(v)-mediated oxidation of the trithiocarbonate in a putative [U^V^(CS_3_){OSi(O*t*Bu)_3_}_4_K] intermediate. Such an intermediate is likely to be formed from the reaction of a U^V^ terminal sulfide, formed from the metathesis of the imido group with a CS_2_ molecule, with a second CS_2_ molecule.

The presence of bound potassium ions incorporated into the structure of uranium siloxide complexes has been shown to have an important effect on the reactivity of U^III^ complexes with CS_2_, and on the stability of the resulting products with respect to trithiocarbonate or tetrathiooxalate ligand loss.^18*a*^ Thus, we anticipated that the analogous reactions carried out with the U^V^ imido complex **5**, where the presence of 2.2.2-cryptand prevents cation binding to the siloxides, might enable us to stabilise the U^V^ terminal sulfide and terminal trithiocarbonate intermediates.

**Scheme 3:**
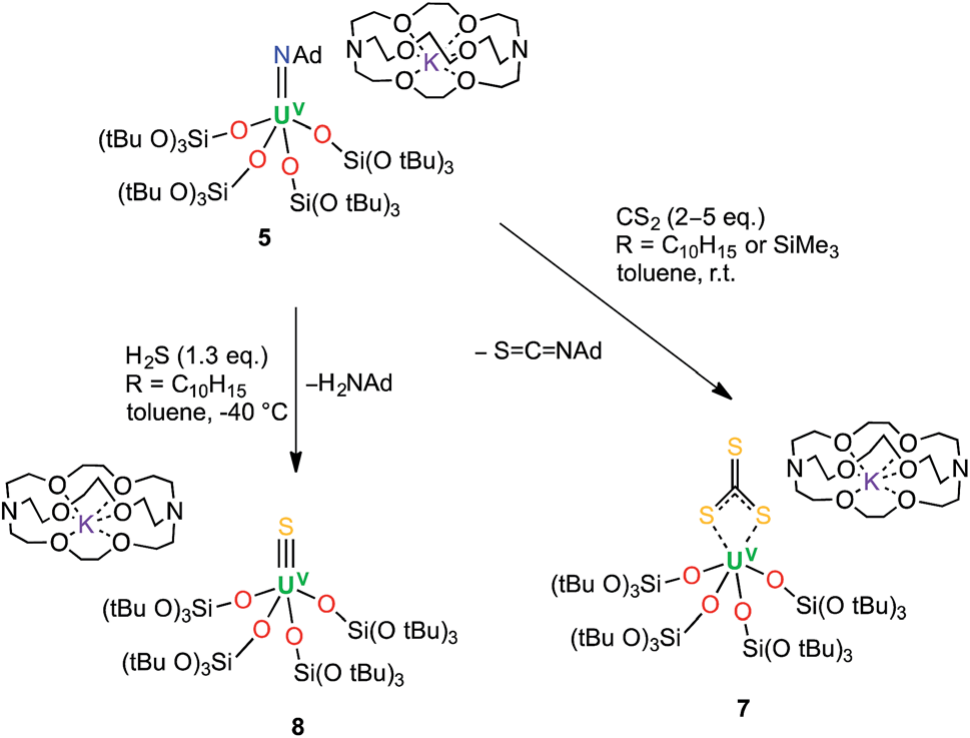
Syntheses of the terminal U^V^ trithiocarbonate complex, [K(2.2.2-cryptand)][U(CS_3_){OSi(O*t*Bu)_3_}_4_] (**7**), and the terminal U^V^ sulfide complex, [K(2.2.2-cryptand)][US{OSi(O*t*Bu)_3_}_4_] (**8**).

Indeed, the reaction of **5** with two to five equivalents of CS_2_ in toluene afforded the trithiocarbonate complex [K(2.2.2-cryptand)][U(CS_3_){OSi(O*t*Bu)_3_}_4_] (**7**) in 57% yield (Scheme 3). The ^1^H NMR spectrum of **7** in d_8_-toluene exhibits two signals with equal integration ratios at 1.77 ppm and 1.51 ppm, respectively, corresponding to the *tert*-butoxy protons of the siloxide ligands, indicating a *C*_2_-symmetric species in solution. The ^13^C NMR spectrum of **7** in toluene shows a broad signal at 180 ppm that is assigned to the bound thiocarbonate ligand. In addition to this signal, the ^13^C NMR spectrum of the crude reaction mixture in d_8_-toluene showed the presence of the isothiocyanate product, S=C=NAd, a resonance at 132 ppm assigned to C_2_S_6_^2–^, and a signal at 247 ppm (free CS_3_^2–^). The ^1^H NMR spectrum of the reaction mixture also shows the presence of a signal assigned to [U{OSi(O*t*Bu)_3_}_4_], but in a much smaller amount (8%) compared to what was found in the reaction of **4** with CS_2_.

**Fig. 4:**
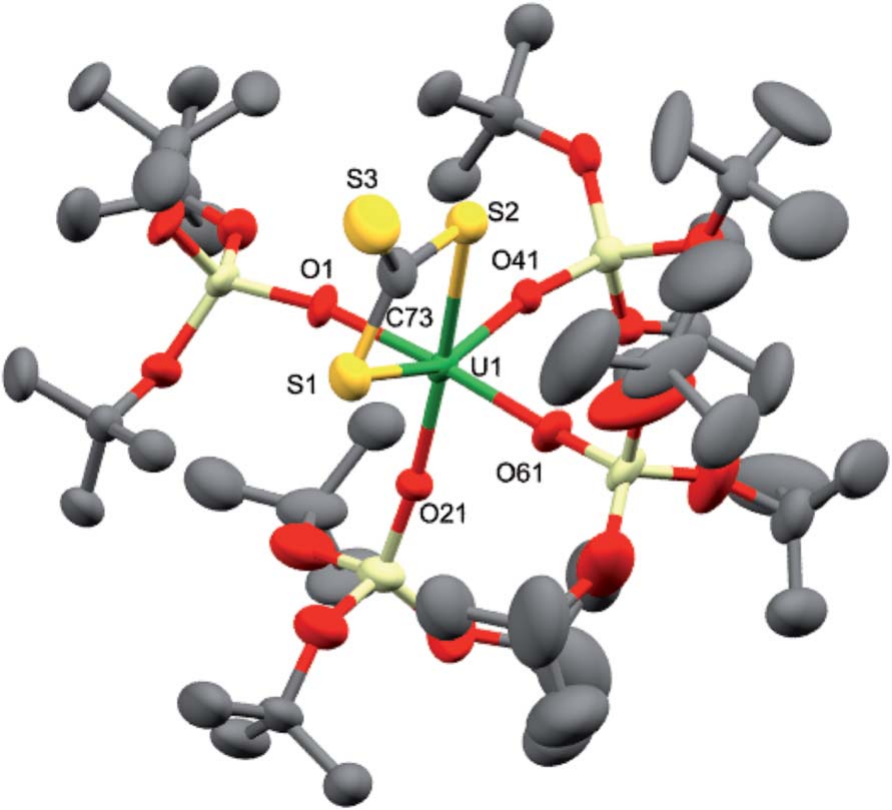
Molecular structure of [U(CS_3_){OSi(O*t*Bu)_3_}_4_]^–^ in crystals of **7**·tol shown with 50% probability thermal ellipsoids. [K(2.2.2-cryptand)]^+^, hydrogen atoms and lattice solvent have been omitted for clarity.

Dark brown crystals of complex **7**·tol crystallised from toluene in the monoclinic space group, *P*2_1_. The molecular structure is shown in Fig. 4 and selected bond lengths are summarised in Table 1. The six-coordinate uranium atom is coordinated by four siloxide oxygen atoms and two sulfur atoms of a terminally-bound κ^2^S–trithiocarbonate moiety, affording a distorted octahedral coordination geometry. The structure bears similarities to the recently reported terminal U^IV^ thiocarbonate [U(Tren^TIPS^)(κ^2^-CS_3_)][K(B15C5)_2_]^20^ and to the related U^IV^ trithiocarbonate complex, [{K(18c6)}_2_{μ_3_-κ^2^:κ^2^:κ^2^-CS_3_}{U(OSi(O*t*Bu)_3_)_4_}].^18*a*^ However, in the latter U^IV^ complex, the 18c6-bound potassium cation is still able to bind two sulfur atoms of the thiocarbonate group. The average U–O bond length (2.10(3) Å) is noticeably shorter than the corresponding average bond lengths in the U^V^ imido complexes, [K(18c6)][U(NSiMe_3_){OSi(O*t*Bu)_3_}_4_] and [K(18c6)][U(NAd){OSi(O*t*Bu)_3_}_4_] (2.16(2) Å and 2.20(2) Å, respectively), and this is presumably a result of greater steric congestion in the two imido complexes, although electronic effects cannot be ruled out. The U–S bond lengths (2.747(3) Å and 2.772(3) Å) are shorter than those in the aforementioned terminal (2.8415(8) and 2.8520(10) Å)^20^ and K(18c6)^+^-capped U^IV^ trithiocarbonate complex (2.9488(19) Å and 2.951(2) Å).^18*a*^ In the case of the capped complex, the difference is greater than would be expected given the difference in ionic radii between U^IV^ and U^V^ (0.13 Å for six-coordinate ions),^21^ probably due to the electron-withdrawing effect of the two coordinated {K(18c6)}^+^ units in the U^IV^ complex. The C–S bond lengths (1.679(13) Å, 1.696(12) Å and 1.749(14) Å) show similar values (within error) as previously observed for the related U^IV^ trithiocarbonate complex, [{K(18c6)]}_2_{κ^2^-CS_3_}{U(OSi(O*t*Bu)_3_)_4_}] (1.723(8), 1.711(10) and 1.704(8) Å),^18*a*^ in agreement with charge delocalisation over the CS_3_^2–^ unit.

**Table 1:** Selected bond lengths (Å) for complexes **7**·tol and **8**·1.5tol

Structural parameters	**7**·tol	**8**·1.5tol
U1–S1	2.772(3)	2.376(5)
U1–S2	2.747(3)	—
U1–O_ave_	2.14(3)	2.10(3)
C73–S_range_	1.68(1)–1.75(1)	—

**Scheme 4:**
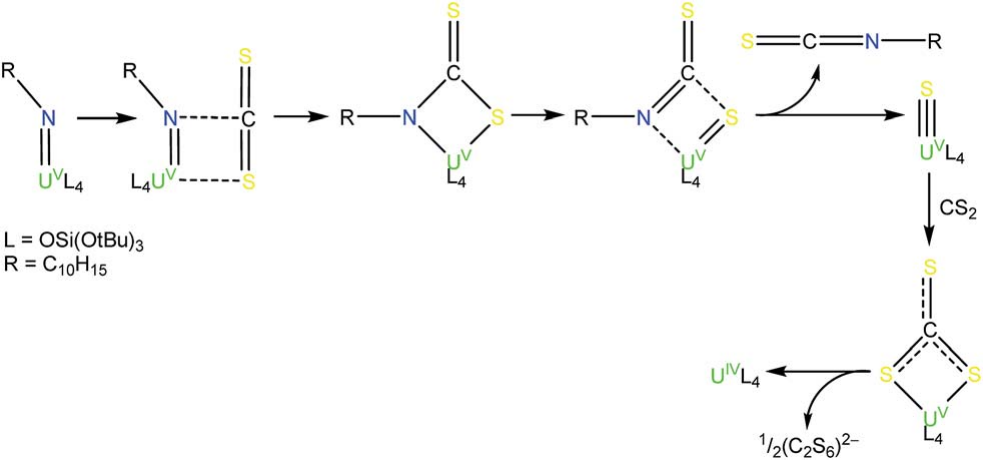
Proposed pathway for the formation of complex **7** from the reaction of complex **5** with CS_2_.

Complex **7** is the first example of a U^V^ uranium trithiocarbonate complex and is only the second example of a terminal trithiocarbonate complex in f element chemistry.^20^ Complex **7** shows higher stability than a putative trithiocarbonate intermediate formed in the reaction of the K^+^ (**4**) U^V^ imido complex with CS_2_. This is probably explained by the fact that in the absence of K^+^ cations binding the trithiocarbonate group, oxidation of the trithiocarbonate by U^V^ is not a favoured pathway. Complex **7** is likely formed by the nucleophilic addition of a U^V^ terminal sulfide intermediate to a CS_2_ molecule (Scheme 4). Fast addition of terminal and bridging U^IV^ sulfide to CS_2_ to afford terminal or bridging U^IV^ thiocarbonate complexes has been previously reported.^4e,22^

Monitoring the reaction between equimolar amounts of complex **5** and ^13^CS_2_ by ^1^H NMR spectroscopy showed a very slow reaction, due in part to the very low solubility of **5** in toluene, and after ten days, complex **7** and unreacted complex **4** were present in equimolar quantities. There is no evidence of the formation of the U^V^ terminal sulfide intermediate under these conditions, probably due to its fast reaction with an additional CS_2_ molecule.

We reasoned that using a less bulky imido complex might increase the rate of the first step of the reaction, thereby allowing for the isolation of a terminal sulfide complex, but NMR-scale reactions between [K(2.2.2-cryptand)][U(NSiMe_3_){OSi(O*t*Bu)_3_}_4_] and two equivalents of ^13^CS_2_ showed that this strategy was unsuitable (see ESI†). The reaction was slow, and although multiple products were formed, it was possible to identify complex **7** in the reaction mixture by ^1^H NMR spectroscopy. The presence of a terminal sulfide was not detected.

These results show that although the metathesis reaction of the U^V^ imido complex with CS_2_ leads to a terminal U^V^ sulfido complex, the reaction is rather slow and the plausible U^V^ terminal sulfide intermediate cannot be isolated due to its rapid reaction with another molecule of CS_2_ to afford the trithiocarbonate complex. In an analogous approach, we anticipated that the high basicity of the imido group could be exploited in an acid/base metathesis reaction with H_2_S to afford a terminal sulfide product. Indeed, treating a pre-chilled (–40 °C) suspension of [K(2.2.2-cryptand)][U(NAd){OSi(O*t*Bu)_3_}_4_] (**4**) in toluene with a fresh, commercially available 0.8 M solution of H_2_S in thf (1.3 eq.) afforded the first isolable U^V^ terminal sulfide complex, [K(2.2.2-cryptand)][US{OSi(O*t*Bu)_3_}_4_] (**8**) (Scheme 3) in 41% yield. Some unidentified side products also formed in the reaction, but a ^1^H NMR spectroscopy experiment using naphthalene as an internal standard showed that the conversion rate to the terminal sulfide product was 76%. The ^1^H NMR spectrum of **8** in d_8_-toluene only shows one broad resonance at 1.20 ppm that corresponds to the *tert*-butoxy protons of the siloxde ligands, along with three signals for the cryptand protons. The fact that only one signal is observed for the siloxide protons suggests that the structure of **8** is fluxional in solution. Complex **8** is reasonably thermally stable and it only showed minor decomposition in solution over the course of a week at room temperature. The formation of the terminal sulfide is likely to involve a double H-atom transfer from the H_2_S to the imido nitrogen. No intermediate reaction product was observed by NMR spectroscopy, suggesting that if the plausible uranium amide/hydrosulfide intermediate is formed (as previously proposed in the hydrosulfidolysis of titanium imido complexes), then the H-transfer from the bound SH to the resulting amido group is fast.^17b,c^

**Fig. 5:**
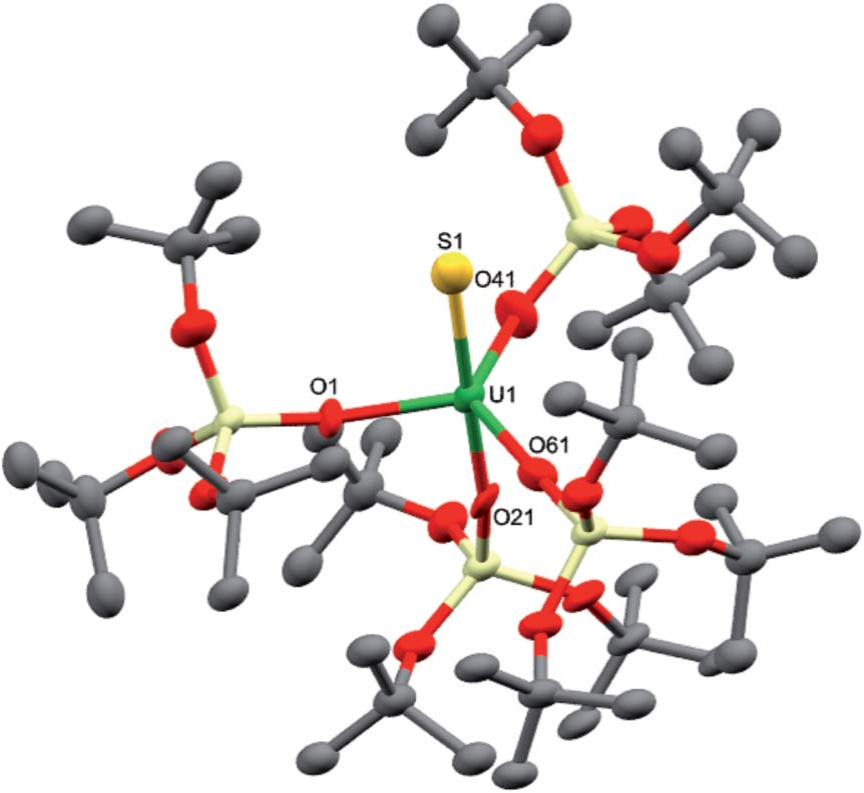
Molecular structure of the anionic fragment of one of the crystallographically independent pairs of [K(2.2.2-cryptand)][US{OSi(O*t*Bu)_3_}_4_] in crystals of **8**·1.5tol shown with 50% probability thermal ellipsoids. [K(2.2.2-cryptand)]^+^, hydrogen atoms and lattice solvent molecules have been omitted for clarity.

Dark brown crystals of complex **8**·1.5tol crystallised from toluene as two crystallographically independent units. The molecular structure is shown in Fig. 5 and selected bond lengths are listed in Table 1. The uranium atoms in each molecule are ligated by one terminally bound sulfide atom and the negatively charged oxygen atoms of four siloxide ligands, resulting in a distorted trigonal bipyramidal coordination geometry. The U–S bond lengths of the two independent molecules are 2.376(5) Å and 2.396(5) Å, respectively, which are considerably shorter than the corresponding bond length in the U^IV^ analogue, [K(2.2.2-cryptand)][US(OSi(O*t*Bu)_3_)_4_K] (2.5220(14) Å).^4*e*^ However, this difference is about what would be expected after accounting for the difference in ionic radii between U^IV^ and U^V^ (0.13 Å).^21^ The predicted values for the U–S double and triple bonds according to Pyykkö are significantly shorter (respectively 2.28 Å and 2.13 Å).^23^ A similar discrepancy between the Pyykkö values and experimental values was also observed for a triply bonded terminal U(vi) sulfide (U≡S = 2.39 Å in the O≡U≡S^2+^ fragment).^6^ The average U–O bond lengths (2.14(3) Å for molecule **1** and 2.13(4) Å for molecule **2**, respectively) are longer than the corresponding average bond length in complex **7**. Given that a sulfide ligand is considerably less bulky than a trithiocarbonate moiety, this difference can probably be ascribed primarily to electronic effects. The Vis/NIR spectrum of **8** in toluene (see ESI†) shows only the presence of four low intensity signals in the 1000–2000 nm region, as found in other U^V^ complexes.^8a,b,10c^

A ^1^H NMR experiment showed that complex **7** reacted immediately with 1 equiv. of ^13^CS_2_ in d_8_-toluene to yield **8** as the only product. This result supports the possibility of **7** as an intermediate in the formation of **8** from **5**.

The X-band EPR spectra of **7** and **8** were measured in a toluene/acetonitrile glass (see ESI†). While no signal was detected at room temperature, an EPR signal, featuring broad linewidths (600 to 800 mT), that unambiguously originates from a metal-centred unpaired electron was observed at 10 K for both complexes. In both cases, the EPR signal was fitted with a rhombic set of *g*-values (*g*_1_ = 1.25; *g*_2_ = 1.03; *g*_3_ = 0.72 for **7** and *g*_1_ = 1.38; *g*_2_ = 1.24; *g*_3_ < 0.6 for **8**) that are comparable to those reported for the octahedral uranium(v) complex, [UO(OSi(O*t*Bu)_3_)_4_K] (*g*_1_ = 1.248; *g*_2_ = 0.856; *g*_3_ = 0.485).^8*b*^

### Computational bonding analysis

**Fig. 6:**
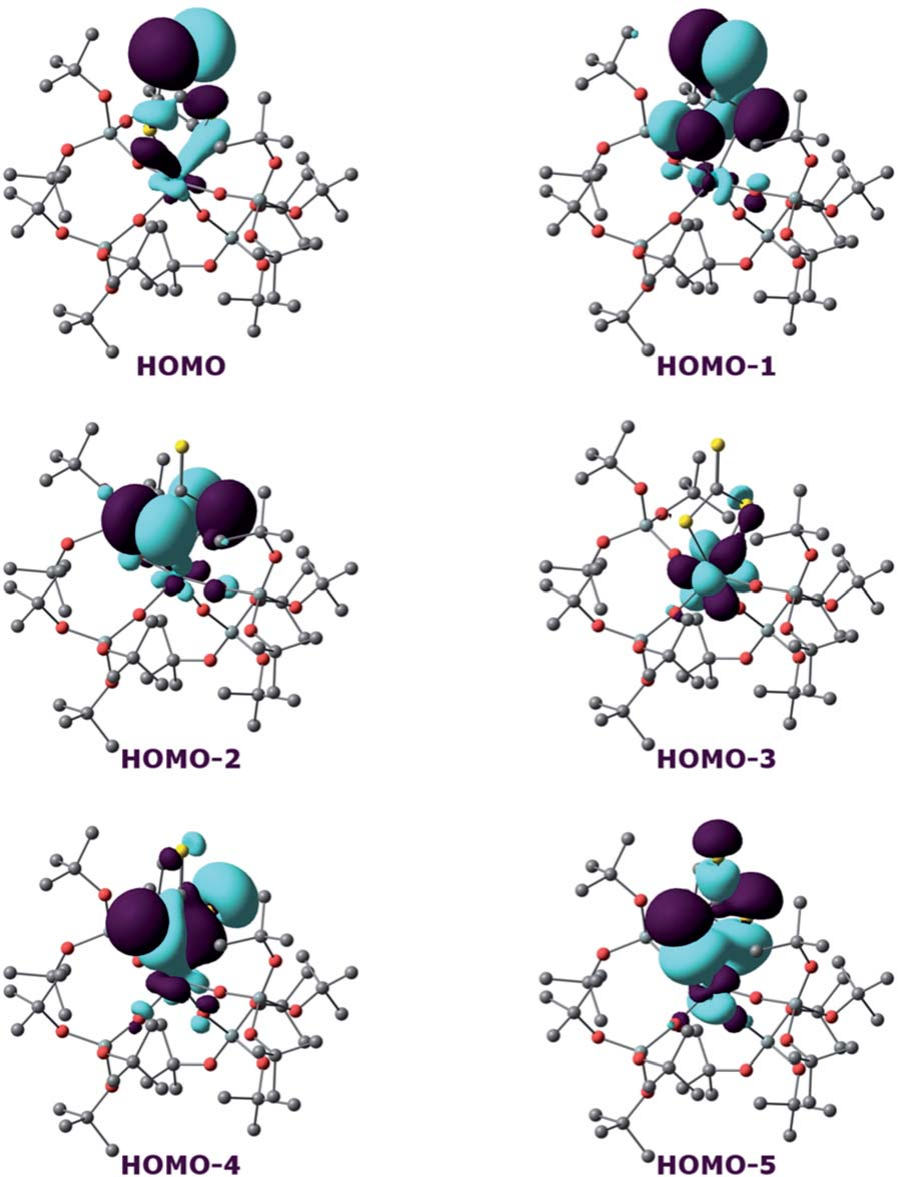
Frontier molecular orbitals of complex **7**.

In order to investigate the nature of the U–S bond in complexes **7** and **8**, we performed calculations at the B3PW91 level, as this method was successfully applied to describe the U–chalcogen bonds in previous studies.^4d,e^ Firstly, the bonding situation was analysed in the U^V^ trithiocarbonate complex (**7**). No clear U–S multiple bond character was found. Rather, two σ U–S bonds (HOMO-4 and HOMO-5 in Fig. 6) and a C=S double bond (HOMO and HOMO-1 in Fig. 6) are found in the MO spectrum. The NBO analysis indicates the same bonding situation, with 77–78% S and 23–22% U, and involve a hybrid 6d/5f orbital at the uranium centre. Finally, the WBI of the U–S bonds are 0.94 and 1.02, in line with a σ bond with highly covalent character. The bonding in the U^IV^ dipotassium trithiocarbonate is quite similar to the one found in **7**. Indeed, two σ U–S bonds are found but these bonds are even more polarised than in **7**, with a contribution of 90% from sulfur. This is reflected in the WBI (only 0.47/0.50), indicating a less covalent bond. However, since the U^IV^ trithiocarbonate complex involves the coordination of two potassium atoms, its putative U^V^ equivalent was computed to check the influence of the two potassium ions on the bonding. In the latter U^V^ complex, the bonding is also consistent with two U–S σ bonds. These bonds appear to be as polarised as in **7**, with a 77/80% contribution from sulfur to the bonding.

**Fig. 7:**
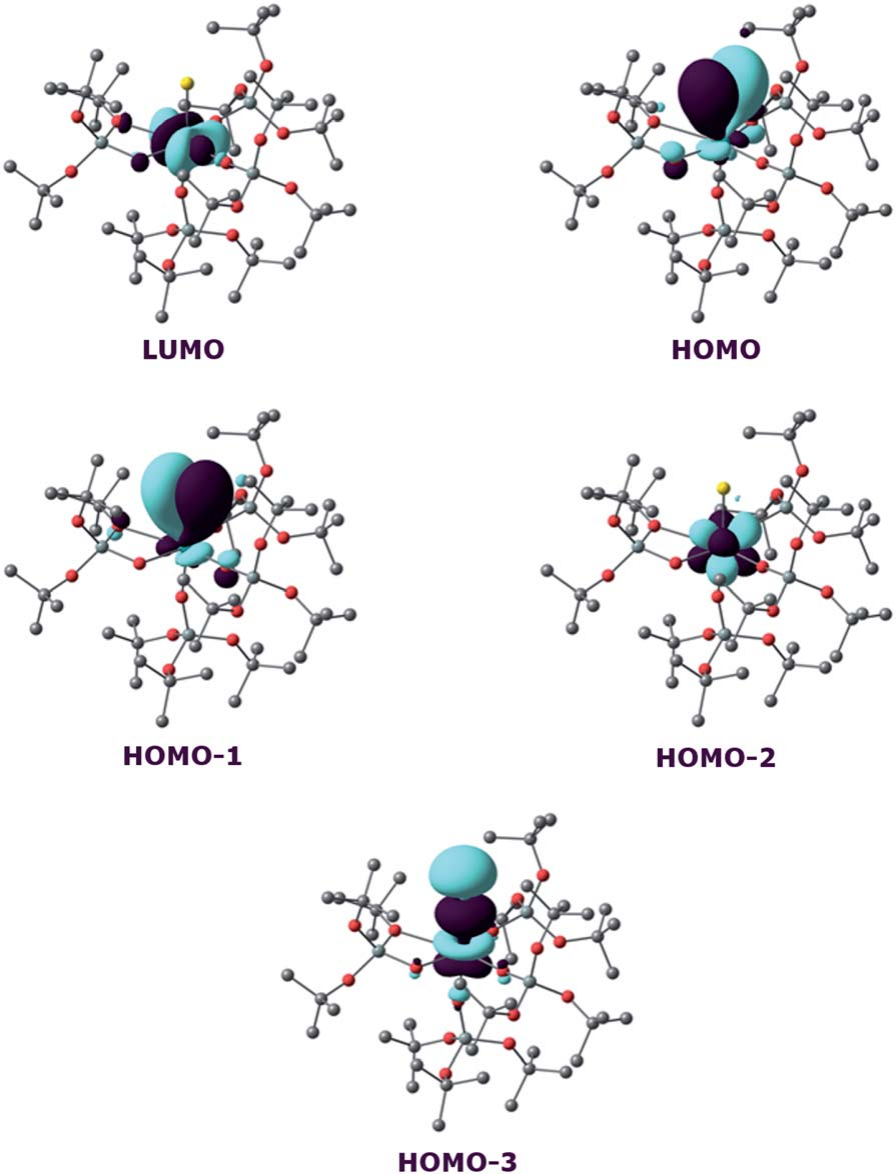
Frontier molecular orbitals of complex **8**.

On the other hand, the WBI are 0.72/0.75, intermediate between the values found for **7** and the U^IV^ compound, in line with an influence of the potassium on the covalency. Indeed, the presence of the interaction between the potassium and the trithiocarbonate decreases the covalency in the U–S bond, mainly because the atomic orbitals of sulfur need to overlap with both U and K. Using similar methods, we analysed the bonding in complex **8** and compared it with the bonding found in its U^IV^ analogue.^4*e*^ Molecular orbital analysis (Fig. 7) clearly indicates a triple bond that is similar to that observed for the U^IV^ analogue. The HOMO-3 is the σ bond, whereas HOMO-1 and HOMO are the two π orbitals. Natural Bonding Orbital (NBO) analysis is in line with this bonding description. Indeed, at the first order, three bonding orbitals (1σ and 2π) are found and they are strongly polarised towards S (77%, 80% and 81% for the σ orbital and the two π orbitals, respectively). Finally, the Wiberg Bond Index (WBI) is 2.2, in line with a triple bond with very strong covalent character. This is very close to the value of 2.25 that was found for the U^IV^ analogue, indicating that oxidation of the U^IV^ complex does not affect the bonding but only removes an electron from one of the 5f orbitals that becomes the LUMO of the U^V^ system (Fig. 7).

## Experimental

### General procedures

Unless otherwise noted, all manipulations were carried out at ambient temperature under an inert atmosphere using Schlenk techniques and an MBraun glovebox equipped with a purifier unit. The water and oxygen level were always kept at less than 1 ppm. Glassware was dried overnight at 150 °C prior to use.

### Starting materials

The solvents were purchased, in their anhydrous form, from Aldrich or Cortecnec (deuterated solvents), conditioned under argon and vacuum distilled from K/benzophenone (toluene, THF) or sodium dispersion (hexane) or dried over 4 Å molecular sieves for one week (DMSO). All reagents were dried under high-vacuum for 5 days prior to use. HOSi(O*t*Bu)_3_ was purified by sublimation prior to use. Depleted uranium turnings were purchased from the “Société Industrielle du Combustible Nucléaire” of Annecy (France). [U(OSi(O*t*Bu)_3_)_4_K] (**2**),^8*b*^ [K(18c6)][U{OSi(O*t*Bu)_3_}_4_],^15^ and [K(18c6)][U(NAd){OSi(O*t*Bu)_3_}_4_] (**1**)^15^ were prepared according to the published procedures. The complex [K(2.2.2-cryptand)][U(NSiMe_3_){OSi(O*t*Bu)_3_}_4_] was prepared from **3** following a procedure analogous to that reported for [K(18c6)][U(NSiMe_3_)(OSi(O*t*Bu)_3_)_4_].^15^

Synthetic details for the preparation of [K(2.2.2-cryptand)][U{OSi(O*t*Bu)_3_}_4_] (**3**), [U(NAd){OSi(O*t*Bu)_3_}_4_K] (**4**), and [K(2.2.2-cryptand)][U(NAd){OSi(O*t*Bu)_3_}_4_] (**5**) are given in the ESI.†

### NMR, IR, Vis/NIR and EPR spectroscopy

NMR spectra were performed in J. Young NMR tubes. ^1^H and ^13^C NMR spectra were recorded on a Bruker 400 MHz spectrometer. NMR chemical shifts are reported in ppm and were referenced to the residual ^1^H and ^13^C signals of the deuterated solvents. IR analyses were performed with a Perkin-Elmer Spectrum One FT-IR Spectrometer. The sample was placed into the Harrick High Temperature Chamber DRIFT cell under an argon atmosphere. Scans were performed in a range between 400 and 4000 cm^–1^ at a resolution of 4 cm^–1^. Vis/NIR spectra were recorded on a Perkin Elmer Lambda 950 instrument. Data were collected in 10 mm path length cuvettes equipped with a J. Young valve. The samples were loaded under argon in the glovebox and were run in toluene. EPR spectra of **7** and **8** were measured with a Bruker Elexsys E500 spectrometer working at 9.4 GHz frequency with an oxford ESR900 cryostat for 4–300 K operation. Baseline correction of the raw EPR spectrum was performed with cubic spline (Xepr 2.4b.12, Bruker). Simulations were performed with the Easyspin 5.1.3 program.^24^

### Elemental analyses

Samples were analysed under nitrogen by the elemental analyses department of the EPFL using a Thermo Scientific Flash 2000 Organic Elemental Analyzer.

### X-ray analyses

Crystallographic data for X-ray analyses of all complexes are given in Table S1.† Figure graphics were generated using MERCURY 3.9: Cambridge, U.K., 2001–2016. CCDC-; 1535285 (**7**), CCDC-; 1535286 (**6**), CCDC-; 1535287 (**8**) CCDC-; 1535288 (**4**) and CCDC-; 1535289 (**3**) contain the supplementary crystallographic data for this paper.†

Bragg-intensities of **3**, **4**, **6**, **7** and **8** were measured at low temperature [100 K and 140 K (compound **8**)], respectively using Cu Kα radiation (*λ* = 1.54184 Å) on a Rigaku SuperNova dual system diffractometer equipped with an Atlas CCD detector for compound **3** and **7** and equipped with an Atlas S2 CCD detector for compound **4**, **6** and **8**. The datasets were reduced and then corrected for absorption with CrysAlisPro.^25^

The solutions and refinements for the structures were performed by SHELXT^26^ and SHELXL-2016 (release 6),^26^ respectively. In the case of **7**, the solution and refinement for the structure were performed by SHELX-97.^27^ The crystal structures were refined using full-matrix least-squares based on *F* ^2^ with all non-hydrogen atoms anisotropically defined. The hydrogen atoms were placed in calculated positions by means of the “riding” model.

In the case of **4**, the structure contained half of a toluene molecule in the asymmetric unit and it was disordered along a two-fold axis. The atoms were refined anisotropically and in order to have a convergent least-squares refinement, distance and similarity restraints (SADI, SIMU, ISOR and FLAT) were applied.

In the case of **6**, the structure was refined as a two-component twin with HKLF 5 file obtained by treating the data with CrysAlisPro^25^ yielding to the value of 0.432(2) for the BASF parameter. One 18c6 is disordered over two positions. The atoms of each orientation were located in difference Fourier map. The major and minor parts were refined anisotropically, but distance and similarity restraints (DFIX, SADI, ISOR and SIMU) were used for a convergent least-squares refinement, yielding to site occupancy ratios of 0.511(5)/0.489(5). The second 18c6 was just partially disordered over two positions but treated in the same way yielding to site occupancy ratios of 0.64(1)/0.36(1).

In compound **7**, light atoms (C and O) showed unstable anisotropic behaviour and restraints (SIMU 0.02 card) were necessary to handle them.

In the case of **8**, the structure was refined as a two-component twin crystal and data (in HKLF 5 format) were obtained by treating the data with CrysAlisPro^25^ yielding to the value of 0.448(1) for the BASF parameter. The structure included one molecule of toluene in the asymmetric unit, it was disordered over an inversion centre and refined in a ‘PART-1’ environment. The atoms were refined anisotropically, but distance and similarity restraints (DFIX and SIMU) were employed for a stable least-squares refinement.

### Synthesis of [K(2.2.2-cryptand)][U(CS_3_){OSi(O*t*Bu)_3_}_4_] (**7**)

[K(2.2.2-cryptand)][U{OSi(O*t*Bu)_3_}_4_] (46 mg, 0.025 mmol) was suspended in toluene (0.5 mL) and then ^13^CS_2_ (7.4 μL, 0.12 mmol) was added by syringe. The mixture was monitored periodically by ^1^H NMR spectroscopy until there was no more starting material (*ca.* 10 days). The product crystallised from solution in two batches and the dark brown crystals of **7** were dried under vacuum (26 mg, 57%). Single crystals suitable for X-ray crystallography were grown from toluene. Anal. calcd for **7** C_67_H_144_KN_2_O_22_S_3_Si_4_U (1815.55): C, 44.32; H, 7.99; N, 1.54. Found C, 44.37; H, 8.22; N, 1.45. ^1^H NMR (400 MHz, d_8_-toluene, 298 K): *δ* [ppm] 3.24 (brs, 12H, 2.2.2-cryptand), 3.13 (brs, 12H, 2.2.2-cryptand), 2.14 (brs, 12H, 2.2.2-cryptand), 1.77 (brs, 54H, OSiO*t*Bu), 1.51 (brs, 54H, OSi(O*t*Bu)_3_). ^13^C NMR (100.6 MHz, d_8_-toluene, 298 K): *δ* [ppm] 180.88 (CS_3_), 77.78 (OSiOC(CH_3_)), 71.80 (OSiOC(CH_3_)), 70.82 (2.2.2-cryptand) 67.81 (2.2.2-cryptand), 54.08 (2.2.2-cryptand), 32.62 (OSiOC(CH_3_)), 27.97 (OSiOC(CH_3_)).

### Synthesis of [K(2.2.2-cryptand)][US{OSi(O*t*Bu)_3_}_4_] (**8**)

[K(2.2.2-cryptand)][U{OSi(O*t*Bu)_3_}_4_] (89 mg, 0.048 mmol) was suspended in toluene (1.5 mL) and the mixture was chilled to –40 °C. A 0.8 M solution of H_2_S in thf (75 μL, 0.060 mmol) was added by syringe, immediately giving a dark brown solution. A slight excess (1.3 eq.) of H_2_S is needed to ensure consumption of the starting material and the reaction is sensitive to the quality of the H_2_S solution that is used. The resulting dark brown solution was stirred overnight at –40 °C and then for two hours at room temperature the following morning. The solvent was then removed under vacuum, leaving a dark brown oil. Hexane (0.5 mL) was added to the oil and then the mixture was dried under vacuum giving a brown solid. The solid was washed with hexane (3 × 1 mL), and then the resulting solid was recrystallised from toluene several times at –40 °C, affording analytically pure dark brown crystals of **8** (36 mg, 41%). Single crystals suitable for X-ray crystallography were grown from toluene at –40 °C. Anal. calcd for **8**·0.8toluene C_71.6_H_150.4_KN_2_O_22_SSi_4_U (1813.12): C, 47.43; H, 8.36; N, 1.55. Found C, 47.46; H, 8.83; N, 1.58. ^1^H NMR (400 MHz, d_8_-toluene, 298 K): *δ* [ppm] 4.08 (s, 12H, 2.2.2-cryptand), 4.01 (t, 12H, 2.2.2-cryptand), 3.05 (t, 12H, 2.2.2-cryptand), 1.20 (brs, 108H, OSi(O*t*Bu)_3_). ^13^C NMR (100.6 MHz, d_8_-toluene, 298 K): *δ* [ppm] 180.88 (CS_3_), 73.76 (OSiOC(CH_3_)), 71.05 (OSiOC(CH_3_)), 56.38 (2.2.2-cryptand), 32.44 (OSiOC(CH_3_)). IR (DRIFT, cm^–1^): 2967s, 2924m, 2898m, 2818m, 1477w, 1459w, 1447w, 1385m, 1360s, 1297w, 1261m, 1239s, 1222m, 1196s, 1133m, 1108s, 1077sh, 1050vs, 1024s, 975s, 957s, 917s, 825m, 801sh, 765w, 755sh, 701m.

A conversion experiment using naphthalene as an internal standard determined the conversion of **4** to **8** to be 76% by ^1^H NMR spectroscopy.

### Reaction of **4** with CS_2_: isolation of [K(18c6)]_2_[C_2_S_6_] (**6**)

^13^CS_2_ (1.6 μL, 0.027 mmol) was added to a dark brown solution of **4** (20 mg, 0.014 mmol) in d_8_-toluene (0.5 mL), and the resulting dark brown solution was kept at room temperature for several days until complete consumption of **4** was observed. Then, 18c6 (3.7 mg, 0.014 mmol) was added. After several days, a few yellow single crystals of [K(18c6)]_2_[C_2_S_6_] (**6**) deposited. The crystals were reproducibly obtained but attempts to isolate larger amounts only gave mixtures of products.

A conversion experiment using naphthalene as an internal standard determined the conversion of **4** into [U{OSi(O*t*Bu)_3_}_4_] to be 35% by ^1^H NMR spectroscopy.

### Reaction of **8** with CS_2_ to afford **7**

A 0.59 M solution of ^13^CS_2_ in d_8_-toluene (5.0 μL, 0.0030 mmol) was added to a brown solution of [K(2.2.2-cryptand)][US{OSi(O*t*Bu)_3_}_4_] (**8**) (4.0 mg, 0.0023 mmol) in d_8_-toluene (0.5 mL). ^1^H NMR spectroscopy showed immediate and complete consumption of **8**, and the appearance of signals corresponding to [K(2.2.2-cryptand)][U(CS_3_){OSi(O*t*Bu)_3_}_4_] (**7**).

### Computational details

All the structures reported in this study were fully optimised with the Becke's 3-parameter hybrid functional combined with the non-local correlation functional provided by Perdew/Wang (denoted as B3PW91).^28^ The Stuttgart–Dresden RECP (relativistic effective core potential) 5*f*-in-valence was used for uranium atom in combination with its adapted basis set.^29^ However, in some cases, the 5*f*-in-core ECP augmented by a *f* polarisation function (*α* = 1.0) was used for the fixed oxidation state IV or V of the uranium atom.^30^ In addition, silicon atoms were treated with the corresponding Stuttgart–Dresden RECP in combination with its adapted basis sets,^31^ each one augmented by an extra set of polarisation functions.^32^ For the rest of the atoms, the 6-31G(d,p) basis set was used.^33^ For analysing the bonding situation in the complexes of interest, we mainly used natural bond orbital analysis (NBO) using Weinhold's methodology.^34^ Also, the Multiwfn program,^35^ was used for obtaining the composition of the molecular orbitals, based on natural atomic orbital method,^36^ as well as the Wiberg bond order analysis in a Löwdin orthogonalised basis. The Chemcraft program was used for the visualisation of the molecular orbitals.^37^

Finally, the GAUSSIAN09 program suite was used in all calculations.^38^

## Conclusions

To summarise, we have prepared and fully characterised the first examples of stable terminal U^V^ sulfide and thiocarbonate complexes using bulky siloxides as supporting ligands.

DFT calculations were performed to investigate the nature of the U–S bond in complexes **7** and **8**, and the results were compared with the analyses of the analogous U^IV^ complexes. Based on this analysis, triple-bond character with strong covalent character is suggested for the U–S bond in the terminal uranium(v) sulfide **8**, in line with previous studies on terminal U^IV^ sulfides. Single-bond character was found for the U–S bond in complex **7**, which turned out to be more covalent than in the U^IV^ analogue.

In conclusion, we have shown that the metathesis of U^V^ imido complexes with CS_2_ or H_2_S provides a convenient route to terminal sulfides. However, the metathesis reaction with CS_2_ was very slow and resulted in nucleophilic addition of the putative sulfide intermediate to CS_2_. Moreover, the presence of siloxide-bound cations in the U^V^ imido precursor resulted in the isolation of a side-reaction product, the perthiodicarbonate, salt [K(18c6)]_2_[C_2_S_6_], resulting from the oxidation of CS_3_^2–^ by U^V^.

In contrast, the metathesis of U^V^ with H_2_S readily forms a stable terminal U^V^ sulfide. The hydrosulfidolysis of uranium imides reported here provide a versatile route to uranium terminal chalcogenides that should be easily extended to other uranium oxidation states and to other chalcogenides. Work in this direction is in progress.
