# Supplementary material for: Metathesis of a UV imido complex: a route to a terminal UV sulfide
Source: Chem Sci. 2017 Jun 5;8(8):5319–28. doi: 10.1039/c7sc01111c (PMC5607896; doi:10.1039/c7sc01111c)
Supplement: Supplementary file 1 [file SC-008-C7SC01111C-s001.pdf]

Electronic Supplementary Material (ESI) for Chemical Science.
This journal is © The Royal Society of Chemistry 2017

Supporting Information for the Manuscript

Metathesis of a U^V Imido Complex: a Route to a Terminal U^V Sulfide

Rory P. Kelly,[a] Marta Falcone,[a] Rosario Scopelliti,[a] Carlos A. Lamfsus,[b] Laurent Maron,[b] Karsten Meyer,[c] and M. Mazzanti*[a]

[a] Institut des Sciences et Ingénierie Chimiques, Ecole Polytechnique Fédérale de Lausanne (EPFL),

1015 Lausanne, Switzerland.

E-mail: marinella.mazzanti@epfl.ch

[b]Université de Toulouse et CNRS INSA, UPS, CNRS, UMR 5215, LPCNO, 135 Avenue de Rangueil, 31077 Toulouse, France.

[c]Department of Chemistry and Pharmacy, Inorganic Chemistry, Friedrich-Alexander University Erlangen-Nürnberg, Egerlandstraße 1, 91058 Erlangen, Germany.

*Correspondence to Prof. Marinella Mazzanti

Contents:

A) Synthesis..........3

B) NMR spectra..........4

C) EPR spectra..........29

D) Electrochemistry..........30

E) IR and Vis/NIR spectra..........31

F) X-ray crystallographic data..........32

G) Computational data..........39

A) Synthesis

Synthesis of [K(2.2.2-cryptand)][U{OSi(OtBu)$_3$}$_4$] (3)

[U{OSi(OtBu)$_3$}$_4$K] (318 mg, 0.239 mmol) was dissolved in toluene (5 mL), giving a dark brown-orange solution. A colourless solution of 2.2.2-cryptand (90 mg, 0.24 mmol) in toluene (2 mL) was added, and then the dark brown-orange suspension was stirred for approximately one hour. The mixture was then filtered, giving a terracotta-coloured solid. The solid was washed with toluene (3 x 2 mL) and then dried under vacuum. The solid was dissolved in thf (1 mL) and then 1 mL of hexane was layered on top of the dark brown-orange solution. Dark brown-orange crystals formed, which were then isolated by decanting the supernatant. Storage of the filtrate at −40 °C afforded another batch of crystals. The two batches of crystals were dried under vacuum (307 mg, 73 %). Anal. calcd for **3**·0.8thf $C_{69.2}H_{150.4}KN_2O_{22.8}Si_4U$ (1765.03): C, 47.09; H, 8.59; N, 1.59. Found C, 47.38; H, 8.63; N, 1.65. ^{1}H NMR (400 MHz, d$_8$-thf, 298 K): δ [ppm] 3.61 (s, 12H, 2.2.2-cryptand), 3.57 (t, 12H, 2.2.2-cryptand), 2.58 (t, 12H, 2.2.2-cryptand), 1.18 (brs, 108H, OSi(OtBu)$_3$).

Synthesis of [U(NAd){OSi(OtBu)$_3$}$_4$K] (4)

[KU{OSi(OtBu)$_3$}$_4$] (135 mg, 0.101 mmol) was dissolved in toluene (1 mL). A solution of AdN_3 (18 mg, 0.10 mmol) in toluene (0.5 mL) was added, leading to immediate bubbling. The resulting dark brown solution was stirred overnight, and then it was concentrated under vacuum to about half of the original volume, and then it was stored at −40 °C. After a couple of hours, dark brown crystals deposited. The solution was decanted and the dark brown crystals were dried under vacuum (83 mg, 56 %). The yield can be improved by collecting further batches but they are contaminated with increasing amounts of impurities. Single crystals suitable for X-ray crystallography were grown from toluene at −40 °C. Anal. calcd for **4** $C_{58}H_{123}KNO_{16}Si_4U$ (1480.08): C, 47.07; H, 8.38; N, 0.95. Found C, 47.15; H, 8.46; N, 0.96. ^{1}H NMR (400 MHz, d$_8$-toluene, 298 K): δ [ppm] 21.81 (brs, 6H, adamantyl), 14.93 (brs, 3H, adamantyl), 10.59 (brs, 3H, adamantyl), 8.43 (brs, 3H, adamantyl), −0.73 (brs, 108H, OSi(OtBu)$_3$).

Synthesis of [K(2.2.2-cryptand)][U(NAd){OSi(OtBu)$_3$}$_4$] (5)

[K(2.2.2-cryptand)][U{OSi(OtBu)$_3$}$_4$] (485 mg, 0.284 mmol) was suspended in toluene (5 mL). A solution of AdN_3 (50 mg, 0.28 mmol) in toluene (1.5 mL) was added slowly, leading to immediate bubbling. The resulting dark brown solution was stirred for one hour and then filtered into another flask. The solution was concentrated under vacuum to about half of the original volume, and then it was stored at −40 °C. Overnight, a large mass of dark brown crystals deposited. The solution was decanted and the dark brown crystals were dried under vacuum (407 mg, 76 %). Single crystals suitable for X-ray crystallography were grown from toluene at −40 °C. Anal. calcd for **5**·0.25toluene $C_{77.75}H_{161}KN_3O_{22}Si_4U$ (1879.61): C, 49.68; H, 8.63; N, 2.24. Found C, 49.65; H, 8.83; N, 2.26. ^{1}H NMR (400 MHz, d$_8$-thf, 298 K): δ [ppm] 26.47 (brs, 6H, adamantyl), 16.24 (brs, 3H, adamantyl), 12.36 (brs, 3H, adamantyl), 9.66 (brs, 3H, adamantyl), 3.62 (s, 12H, 2.2.2-cryptand), 3.58 (t, 12H, 2.2.2-cryptand), 2.60 (t, 12H, 2.2.2-cryptand), −0.87 (brs, 108H, OSi(OtBu)$_3$). ^{1}H NMR (400 MHz, d$_8$-toluene, 298 K): δ [ppm] 26.98 (brs, 6H, adamantyl), 16.72 (brs, 3H, adamantyl), 12.84 (brs, 3H, adamantyl), 10.03 (brs, 3H, adamantyl),

3.09 (s, 12H, 2.2.2-cryptand), 3.00 (s, 12H, 2.2.2-cryptand), 2.03 (s, 12H, 2.2.2-cryptand), −0.48 (brs, 108H, OSi(OtBu)$_3$).

Synthesis of [K(2.2.2-cryptand)][U(NSiMe$_3$){OSi(OtBu)$_3$}$_4$]

This complex was synthesised by the same procedure that was published for [K(18c6)][U(NSiMe$_3$){OSi(OtBu)$_3$}$_4$]. It was isolated as a dark brown crystalline material in 38 % yield. Anal. Calcd for $C_{69}H_{153}KN_3O_{22}Si_5U$ (1794.52): C, 46.18; H, 8.59; N, 2.34. Found C, 46.39; H, 8.38; N, 2.36. ^{1}H NMR (400 MHz, d$_8$-toluene, 298 K): δ [ppm] 13.74 (brs, 9H, NSiMe$_3$), 3.76 (s, 12H, 2.2.2-cryptand), 3.66 (s, 12H, 2.2.2-cryptand), 2.67 (s, 12H, 2.2.2-cryptand), −0.19 (brs, 108H, OSi(OtBu)$_3$).

B) NMR Spectra

Note: L = OSi(OtBu)$_3$

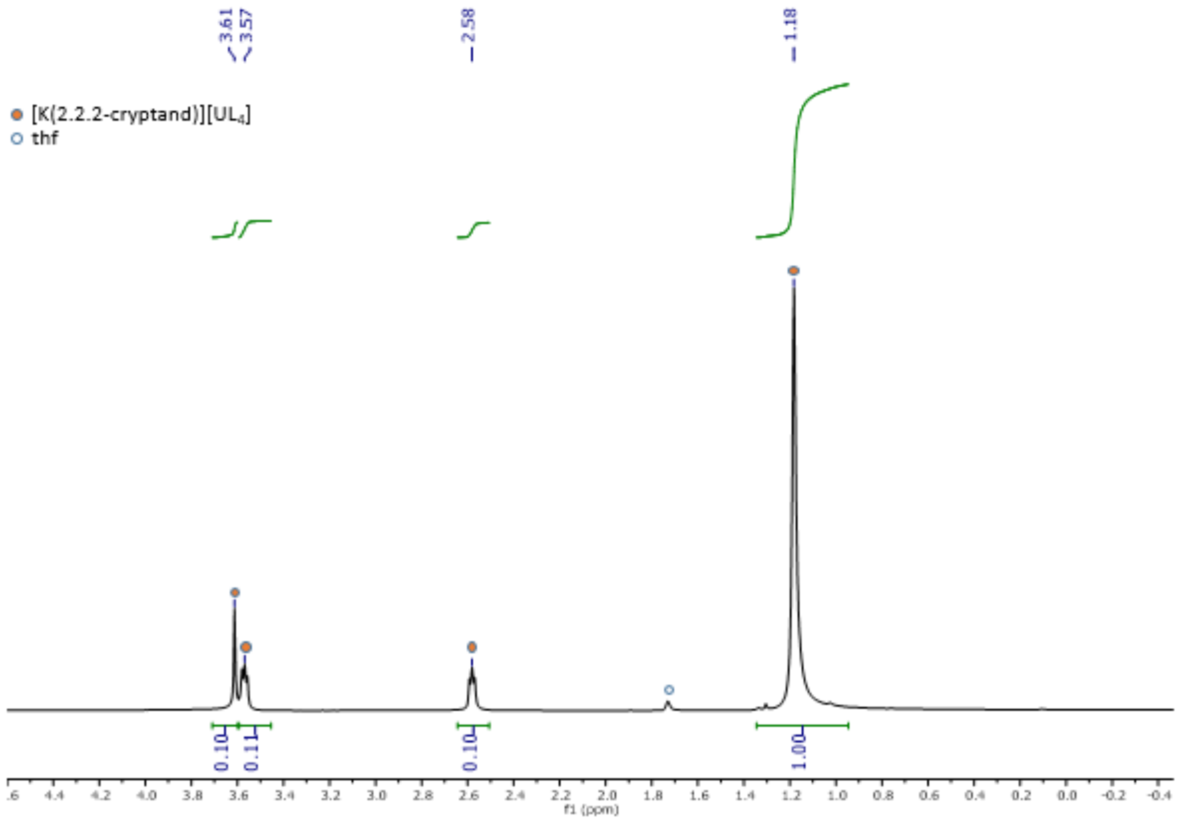

Fig. S1 ^{1}H NMR spectrum of [K(2.2.2-cryptand)][U{OSi(OtBu)$_3$}$_4$] (**3**) (400 MHz, d$_8$-thf, 298 K).

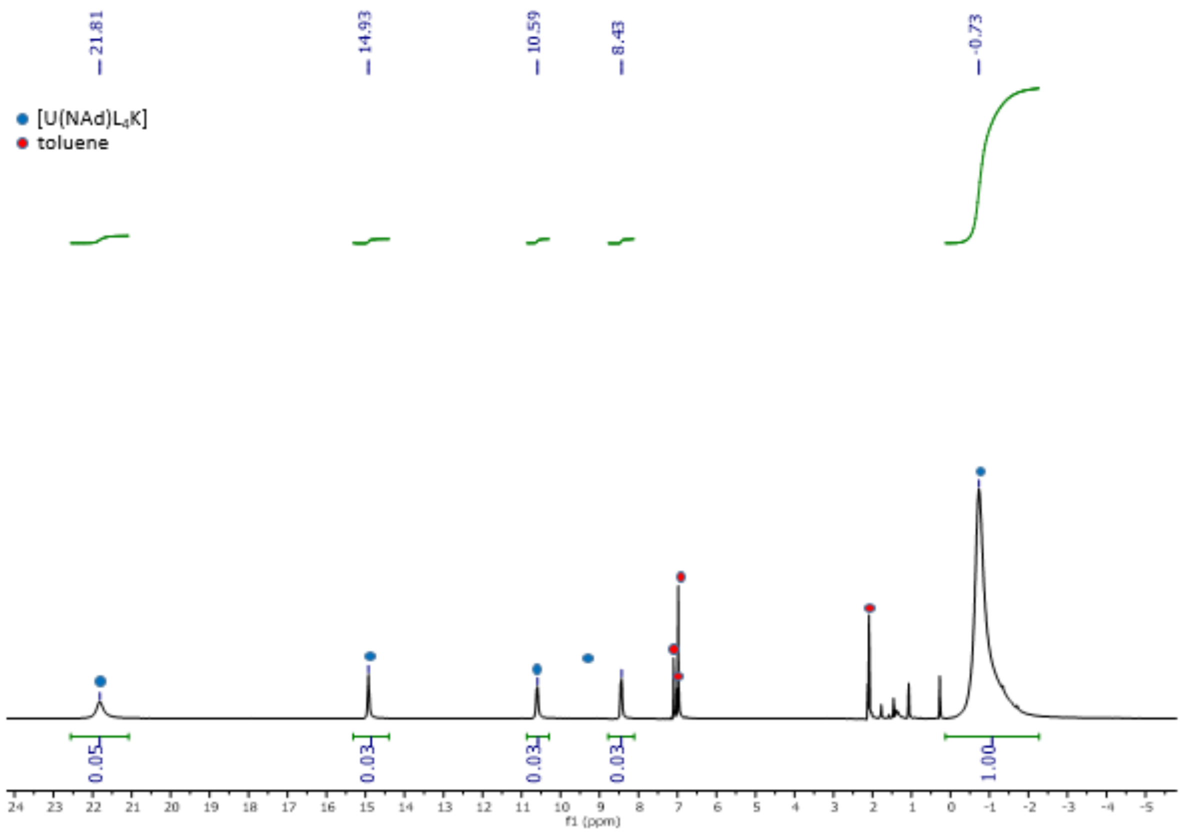

Fig. S2 ^{1}H NMR spectrum of [U(NAd){OSi(OtBu)$_3$}$_4$K] (**4**) (400 MHz, d$_8$-toluene, 298 K).

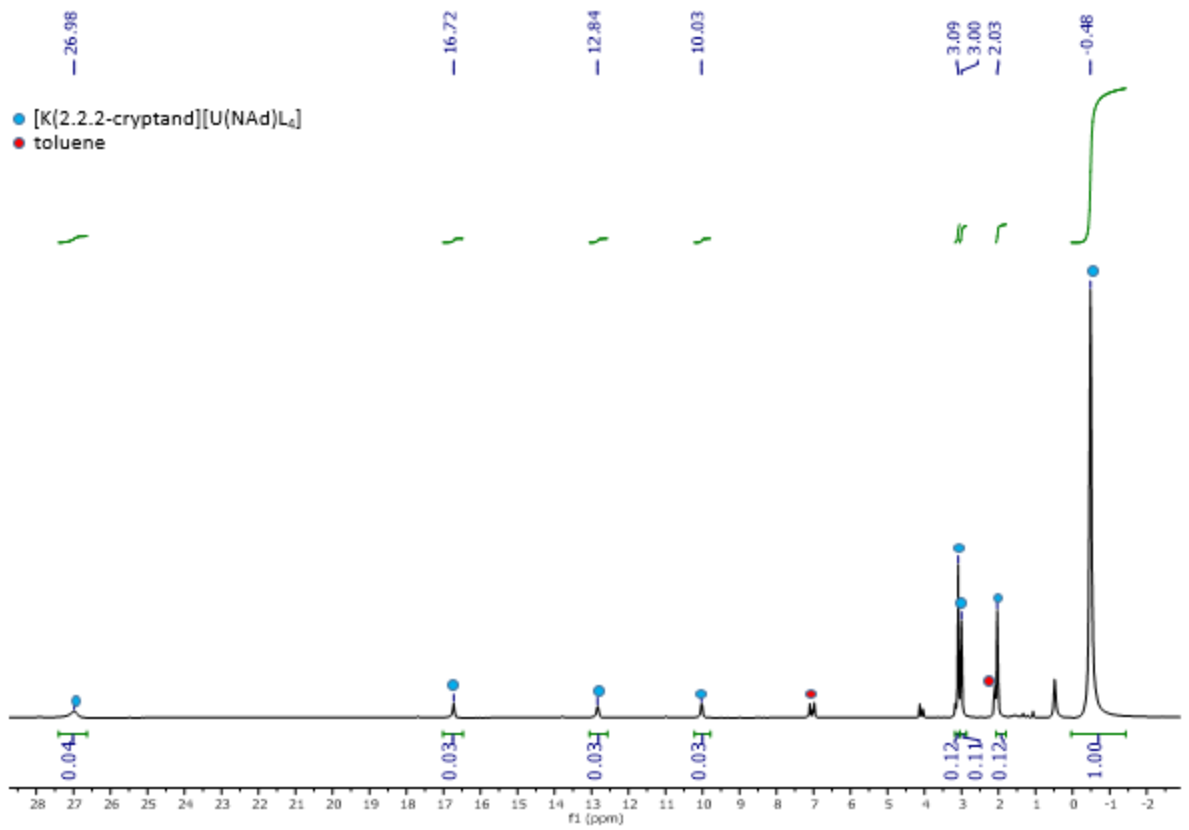

Fig. S3 ^{1}H NMR spectrum of [K(2.2.2-cryptand)][U(NAd){OSi(OtBu)$_3$}$_4$] (**5**) (400 MHz, d$_8$-toluene, 298 K).

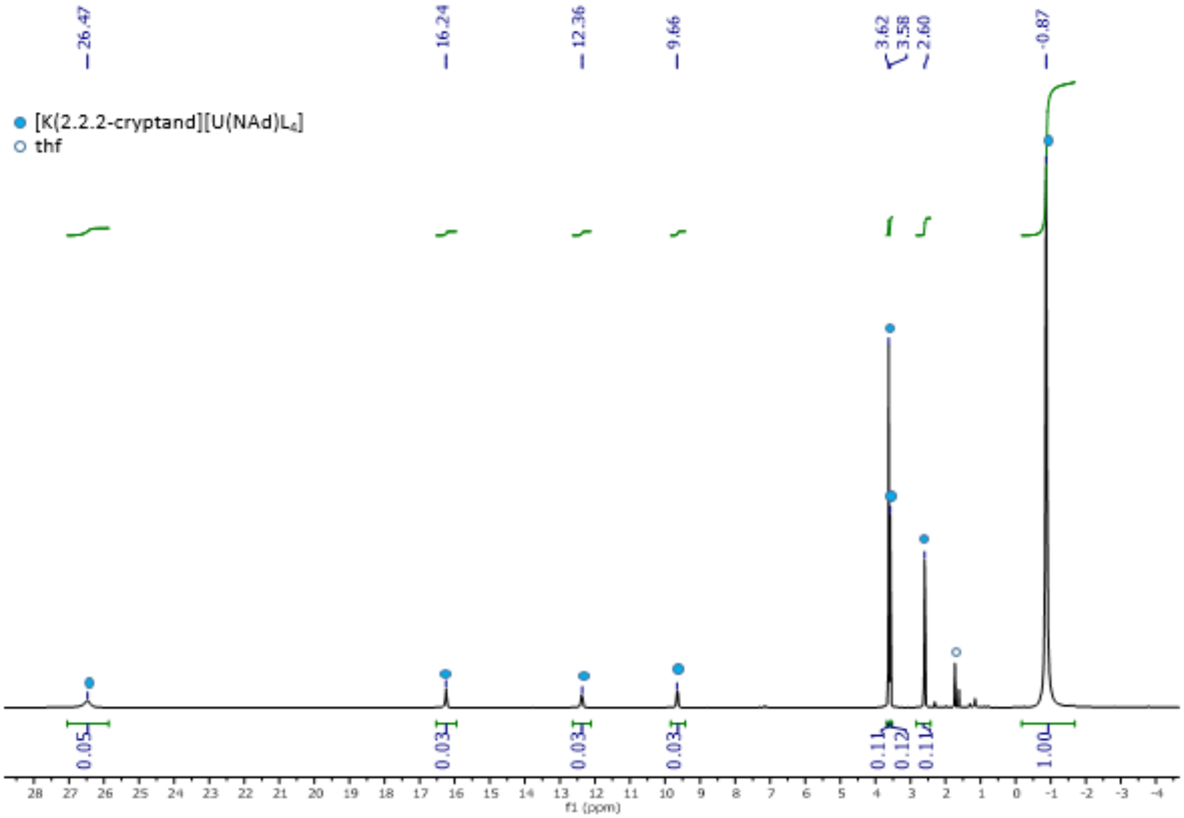

Fig. S4 ^{1}H NMR spectrum of [K(2.2.2-cryptand)][U(NAd){OSi(OtBu)$_3$}$_4$] (**5**) (400 MHz, d_8-thf, 298 K).

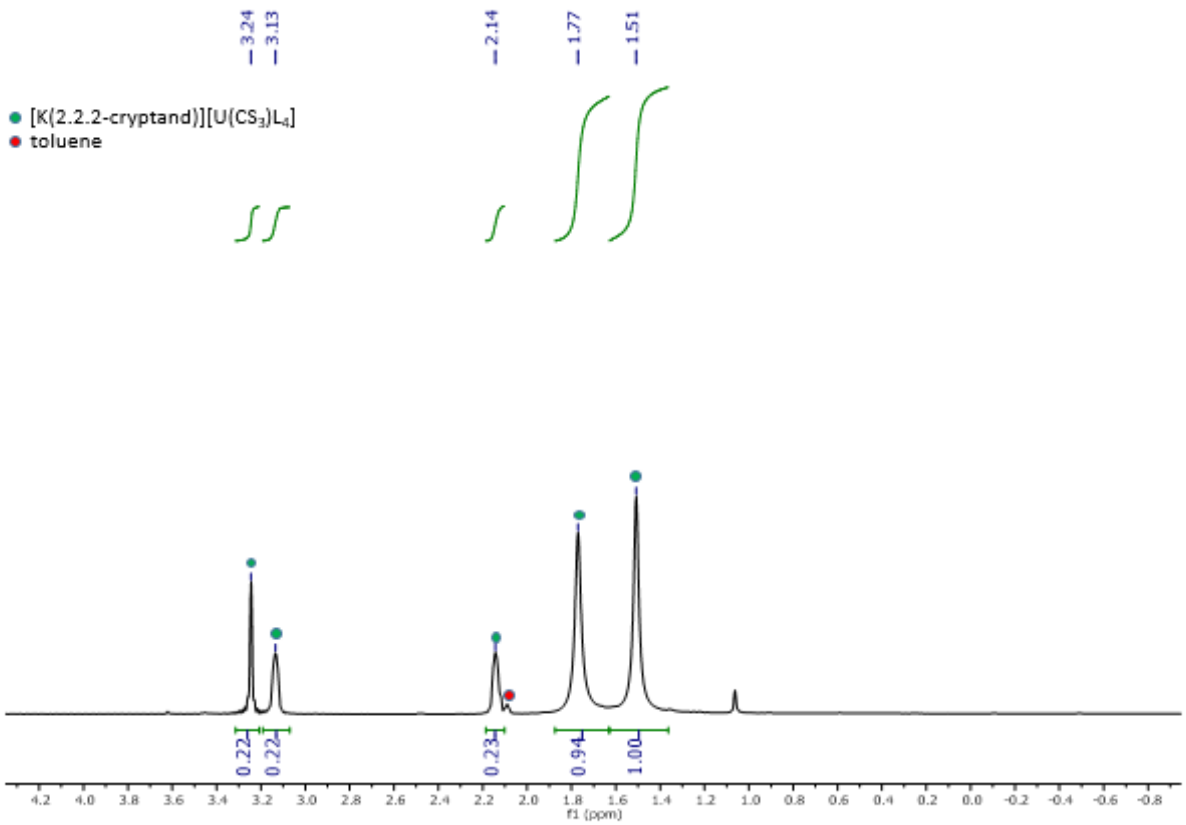

Fig. S5 ^{1}H NMR spectrum of [K(2.2.2-cryptand)][U(CS_3){OSi(OtBu)$_3$}$_4$] (**7**) (400 MHz, d_8-toluene, 298 K).

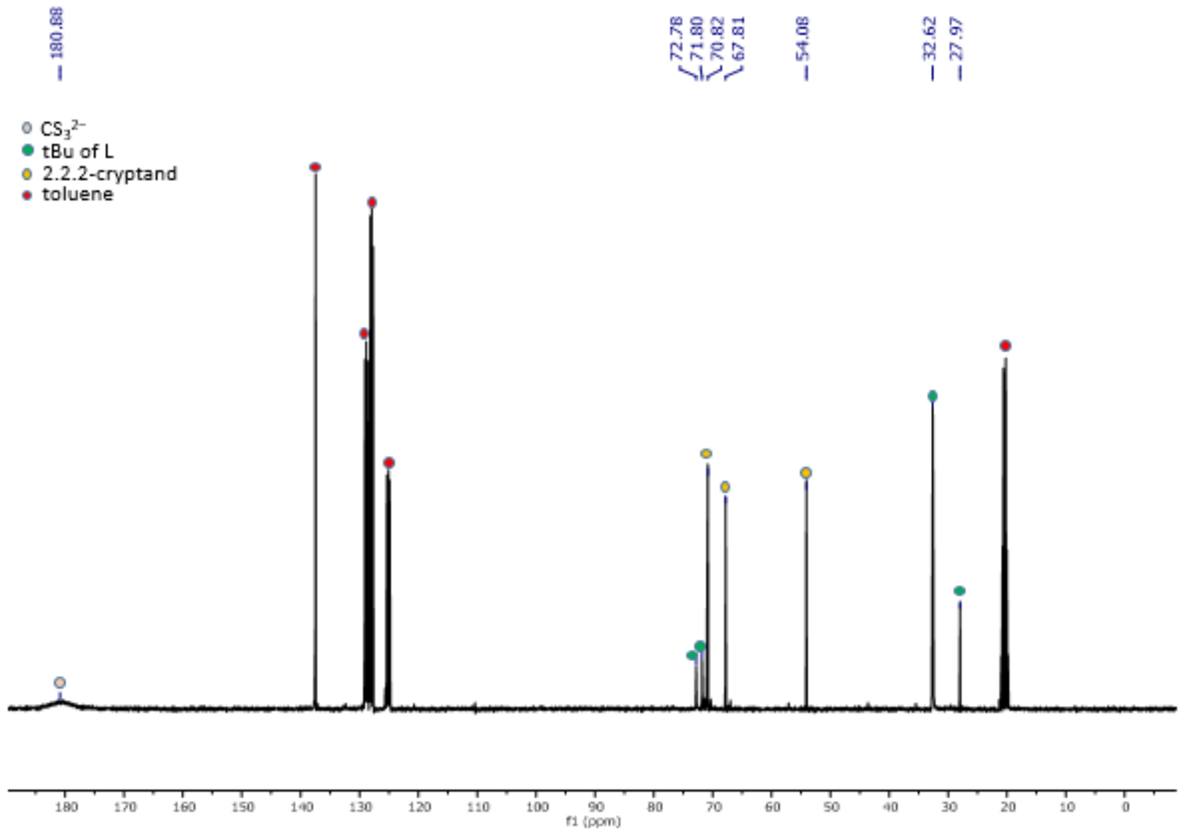

Fig. S6 ^{13}C NMR spectrum of [K(2.2.2-cryptand)][U(CS_3){OSi(OtBu)$_3$}$_4$] (**7**) (400 MHz, d_8-toluene, 298 K).

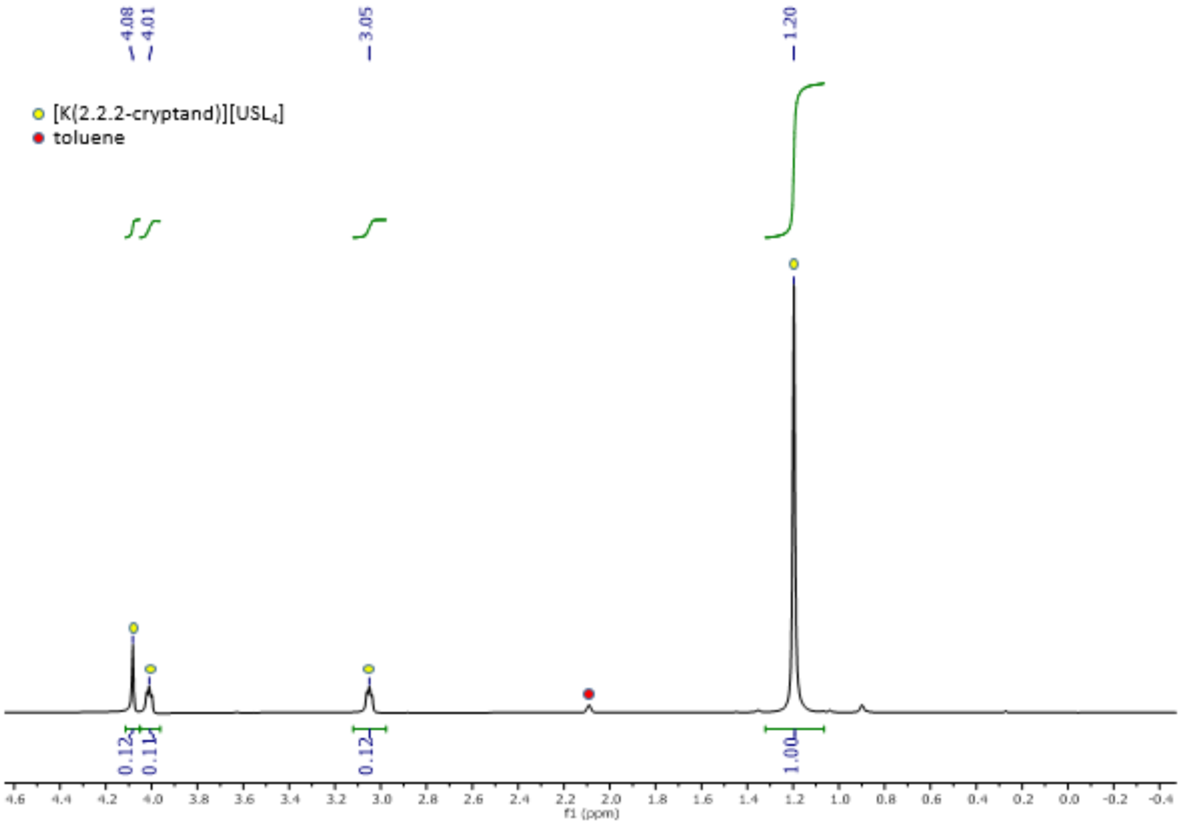

Fig. S7 ^{1}H NMR spectrum of [K(2.2.2-cryptand)][US{OSi(OtBu)$_3$}$_4$] (**8**) (400 MHz, d_8-toluene, 298 K).

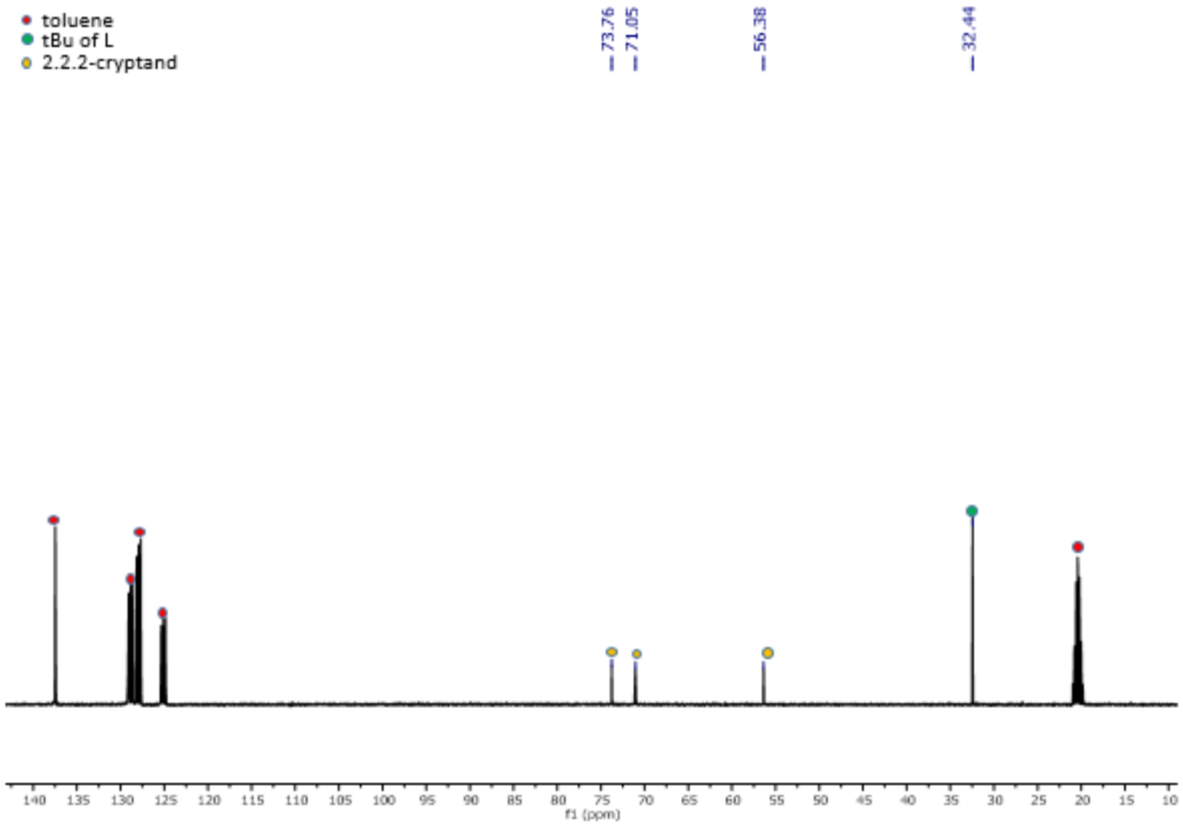

Fig. S8 ^{13}C NMR spectrum of [K(2.2.2-cryptand)][US{OSi(OtBu)$_3$}$_4$] (**8**) (400 MHz, d_8-toluene, 298 K).

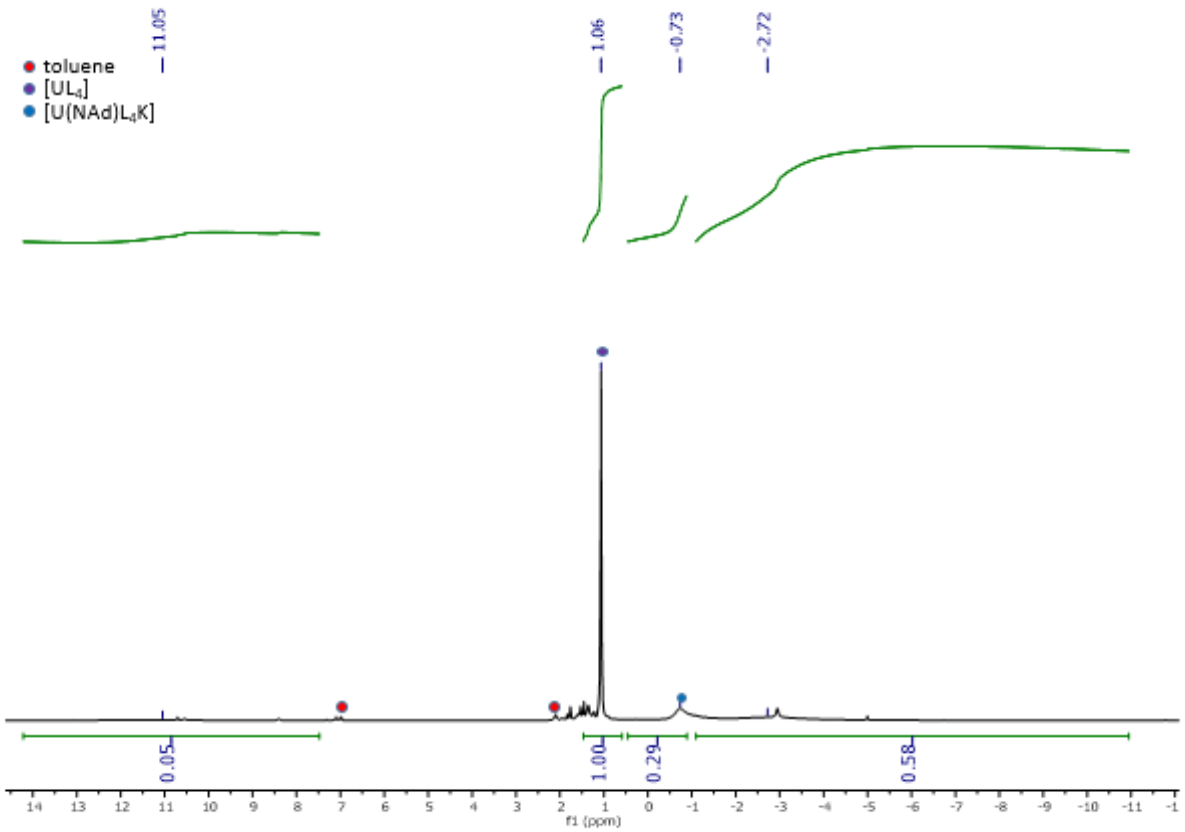

Fig. S9 ^{1}H NMR spectrum (400 MHz, d_8-toluene, 298 K) of the crude reaction mixture 2–3 days after the addition of $^{13}CS_2$ (1 eq.) to a solution of **4** (1 eq.).

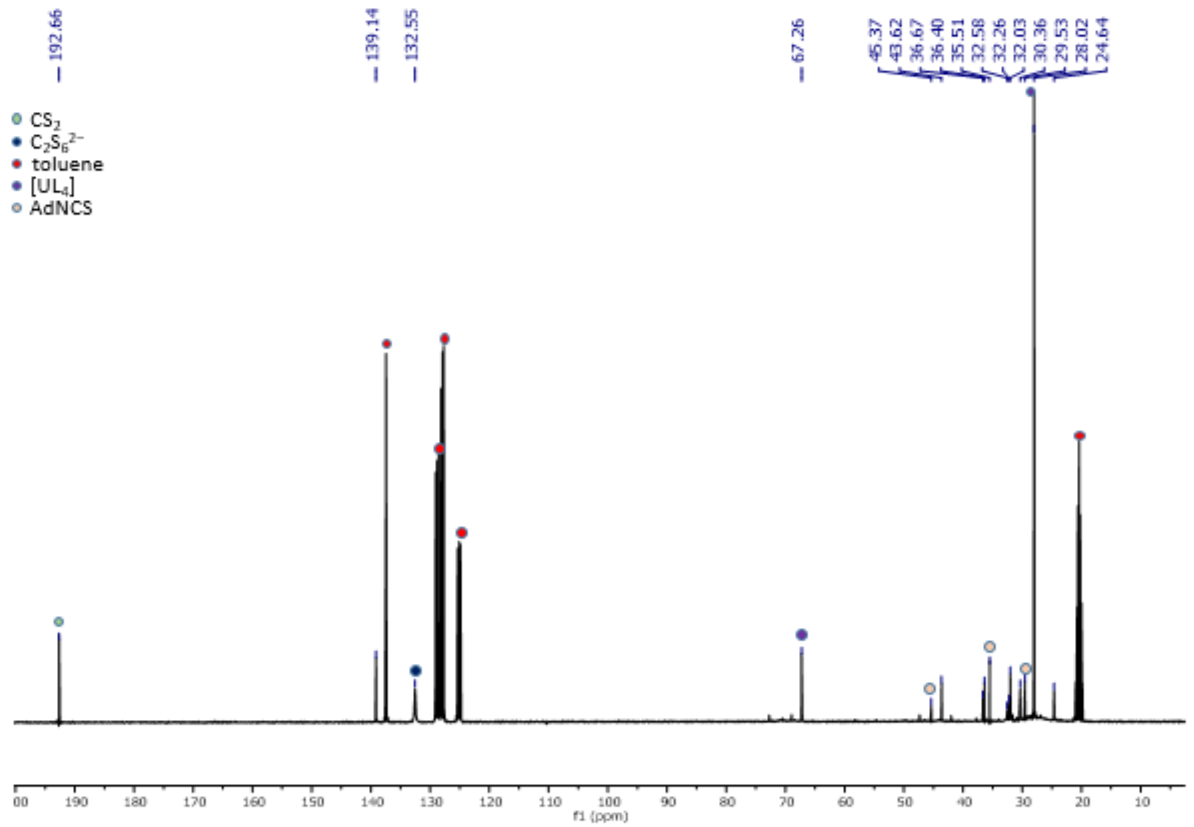

Fig. S10 ^{13}C NMR spectrum (100.6 MHz, d_8-toluene, 298 K) of the crude reaction mixture 2–3 days after the addition of $^{13}CS_2$ (1 eq.) to a solution of **4** (1 eq.).

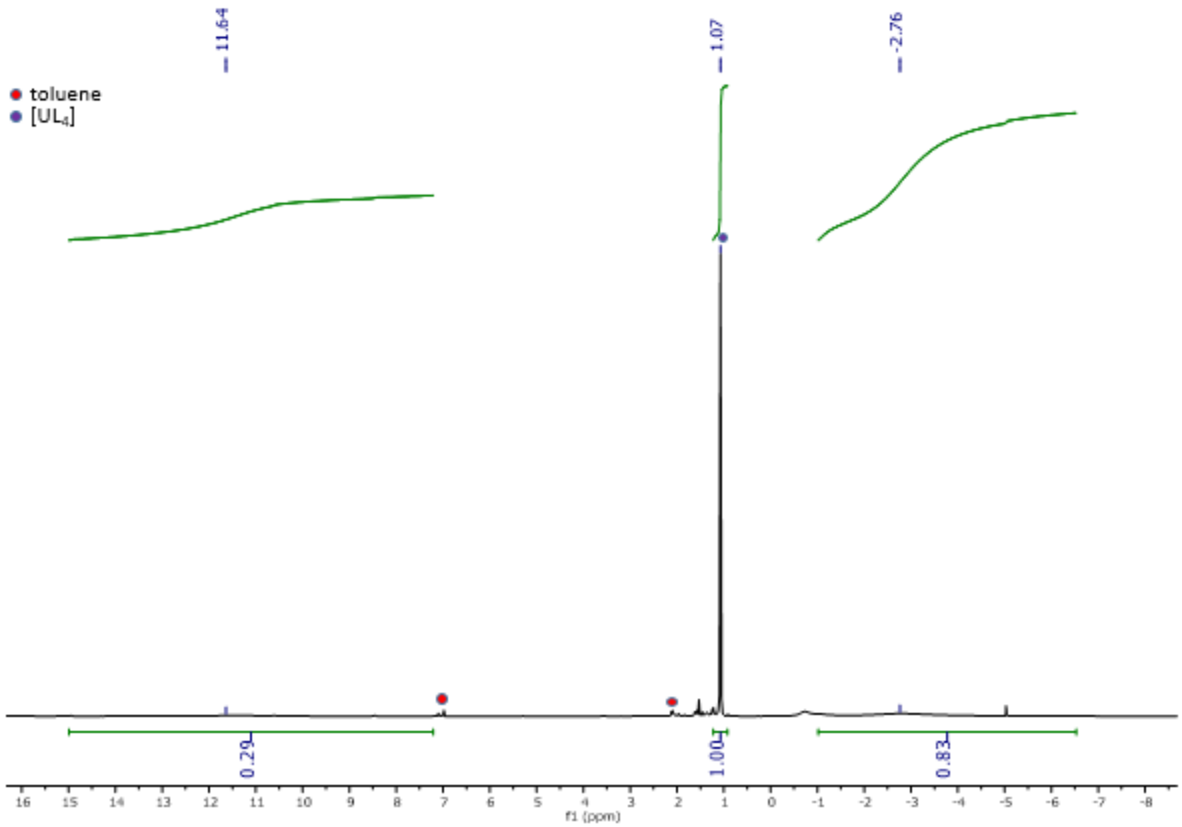

Fig. S11 ^{1}H NMR spectrum (400 MHz, d_8-toluene, 298 K) of the crude reaction mixture 2–3 days after the addition of $^{13}CS_2$ (2 eq.) to a solution of **4** (1 eq.). The conversion of **4** into $[U\{OSi(OtBu)_3\}_4]$ was determined to be 35 % by quantitative ^{1}H NMR spectroscopy with naphthalene as an internal standard.

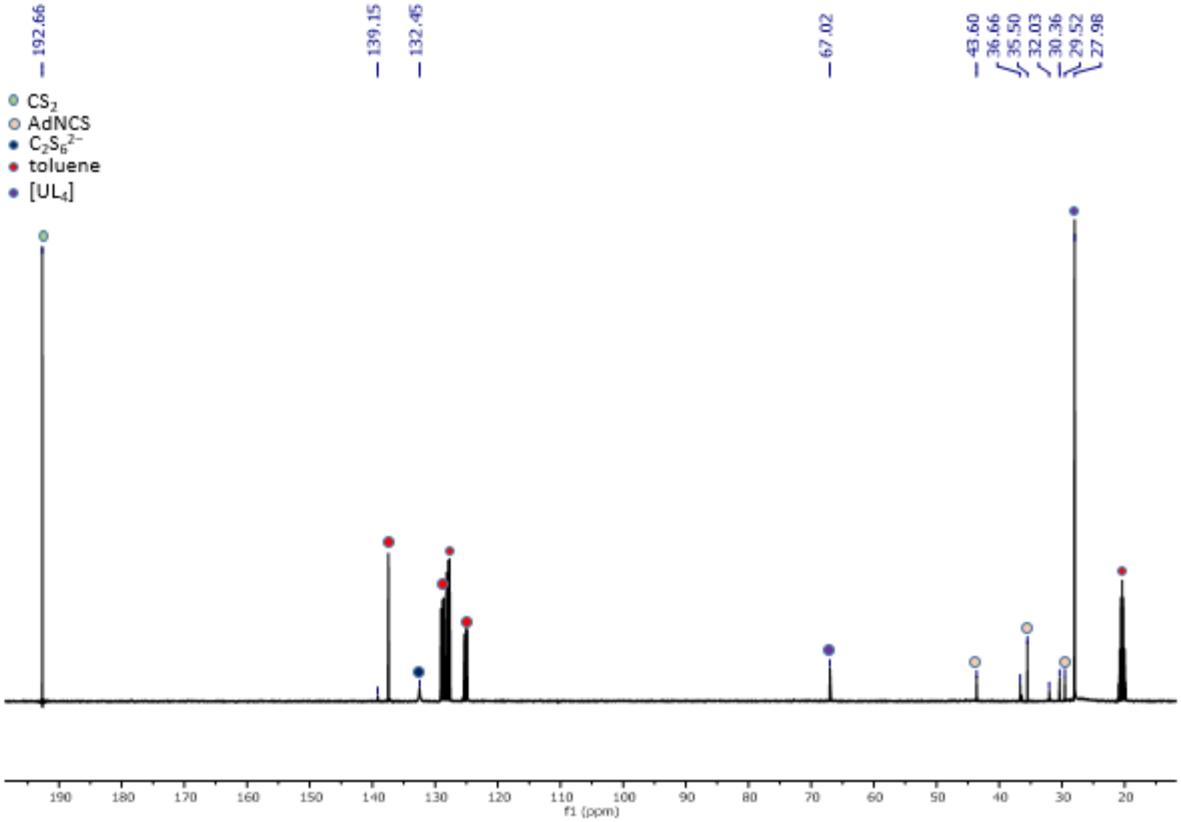

Fig. S12 ^{13}C NMR spectrum (100.6 MHz, d_8-toluene, 298 K) of the crude reaction mixture 2–3 days after the addition of $^{13}CS_2$ (2 eq.) to a solution of **4** (1 eq.) (a solid is also present in the reaction mixture).

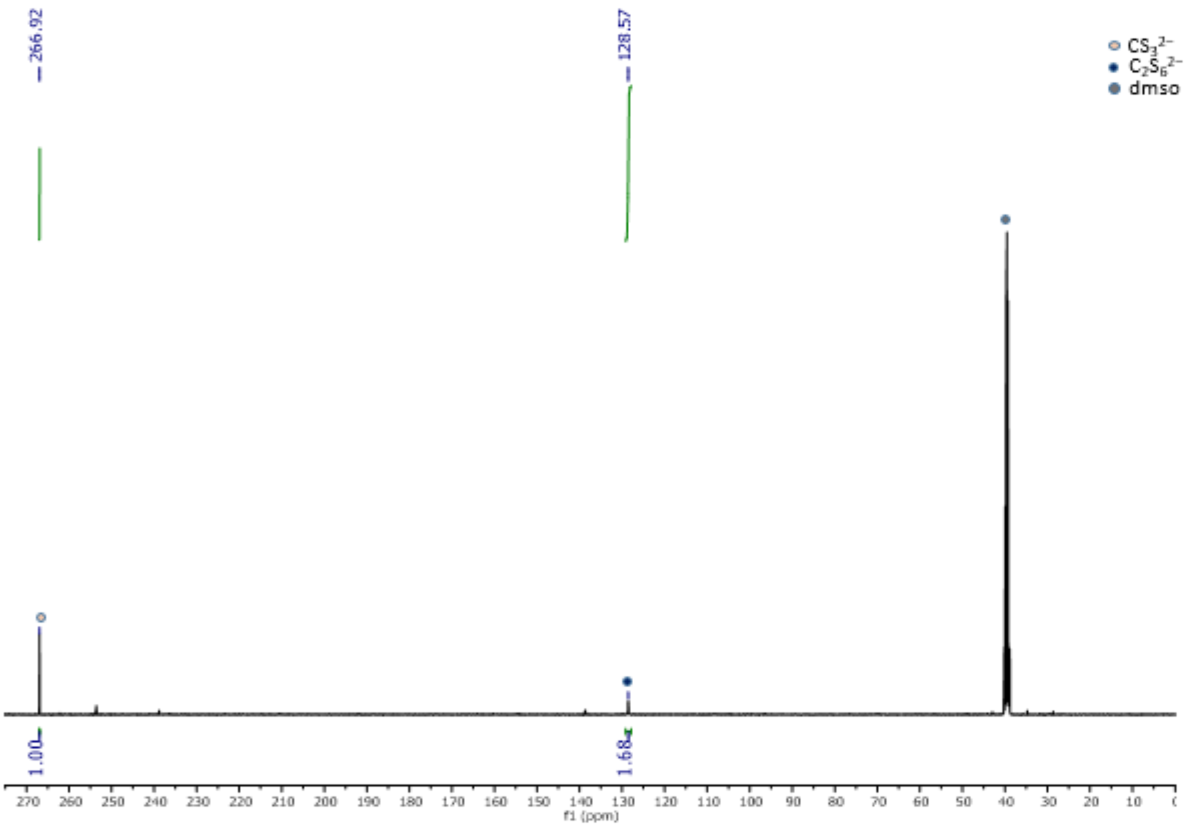

Fig. S13 ^{13}C NMR spectrum (100.6 MHz, d_6-dmso, 298 K) of the residue obtained after toluene was removed from the crude reaction mixture resulting from the addition of $^{13}CS_2$ (2 eq.) to a solution of **4** (1 eq.).

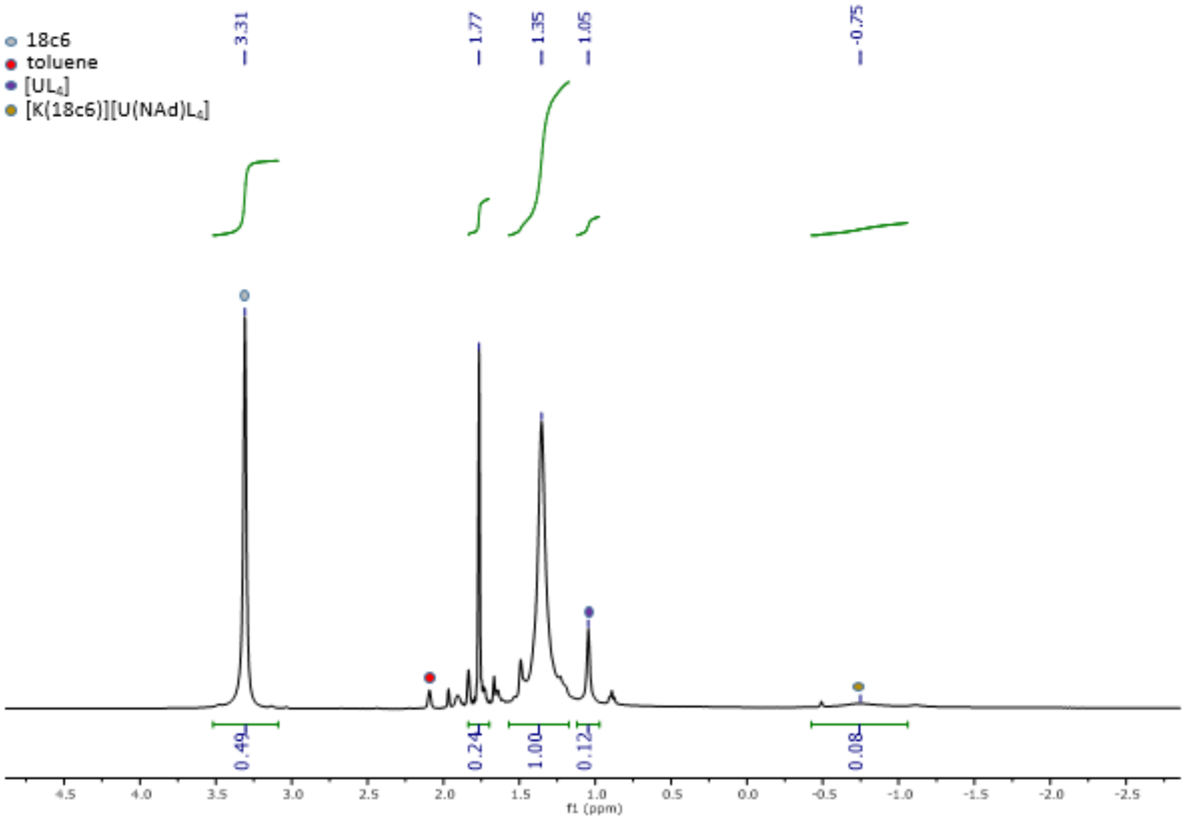

Fig. S14 ^1H NMR spectrum (400 MHz, d_8-toluene, 298 K) of the crude reaction mixture 2–3 days after the addition of $^{13}CS_2$ (1 eq.) to a solution of **1** (1 eq.).

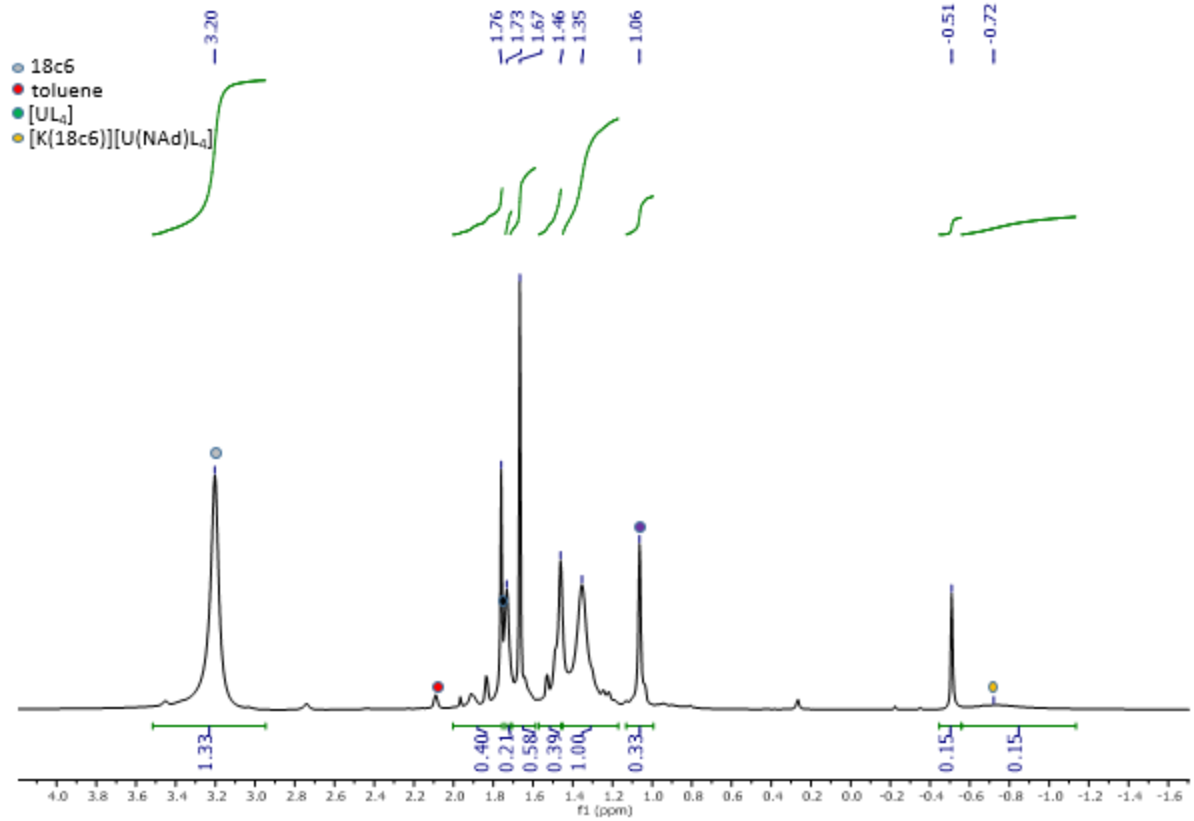

Fig. S15 ^{1}H NMR spectrum (400 MHz, d_8-toluene, 298 K) of the crude reaction mixture 2–3 days after the addition of $^{13}CS_2$ (2 eq.) to a solution of **1** (1 eq.).

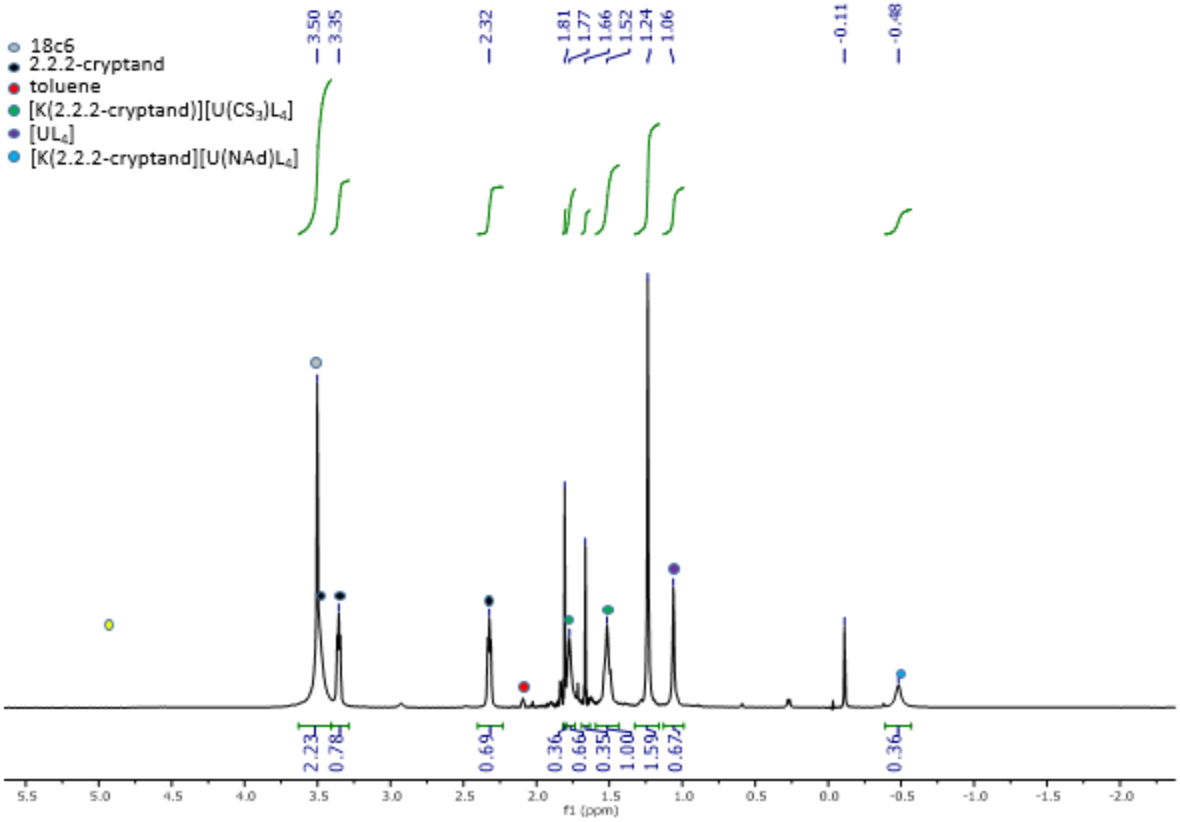

Fig. S16 ^{1}H NMR spectrum (400 MHz, d_8-toluene, 298 K) after addition of 2.2.2-cryptand (1 eq.) to the crude reaction mixture obtained three days after the addition of $^{13}CS_2$ (2 eq.) to a solution of **1** (1 eq.). The conversion of **1** into [U{OSi(OtBu)$_3$}$_4$] was determined to be 9 % by quantitative ^{1}H NMR spectroscopy with naphthalene as an internal standard.

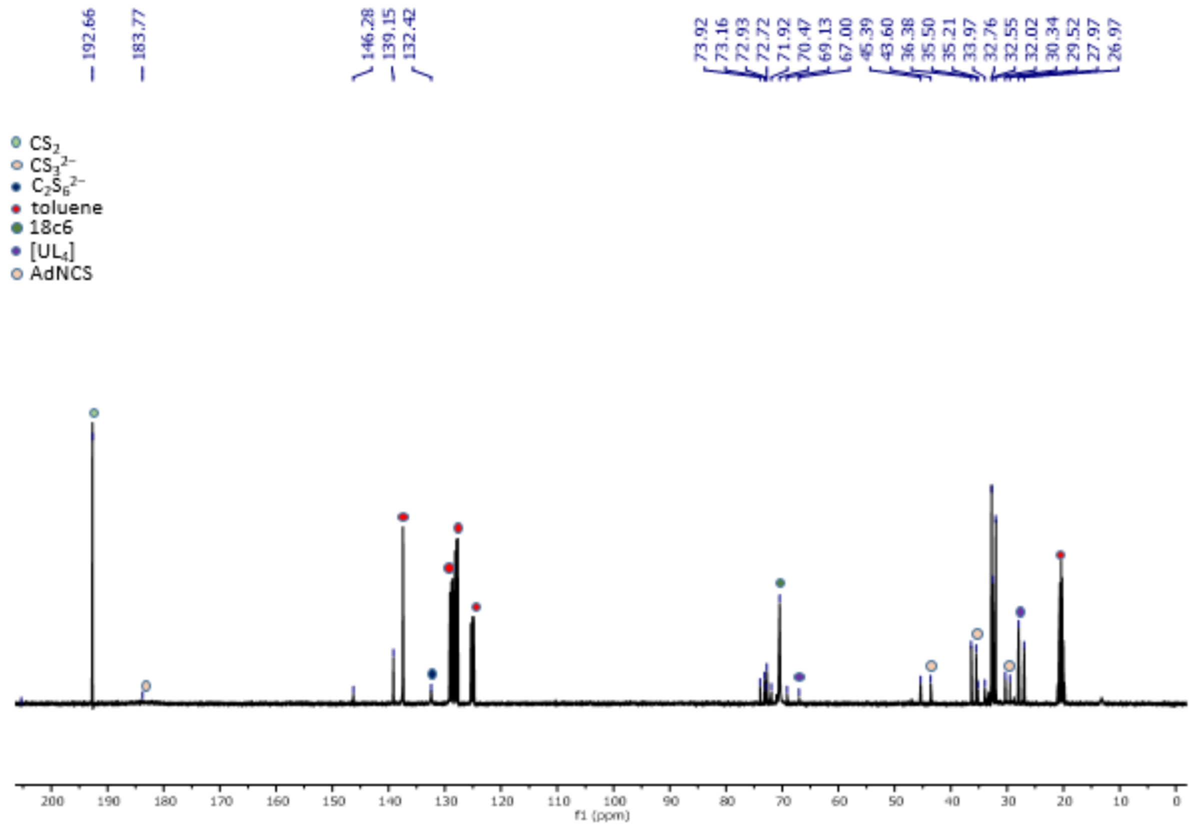

Fig. S17 ^{13}C NMR spectrum (100.6 MHz, d_8-toluene, 298 K) of the crude reaction mixture 2–3 days after the addition of $^{13}CS_2$ (2 eq.) to a solution of **1** (1 eq.).

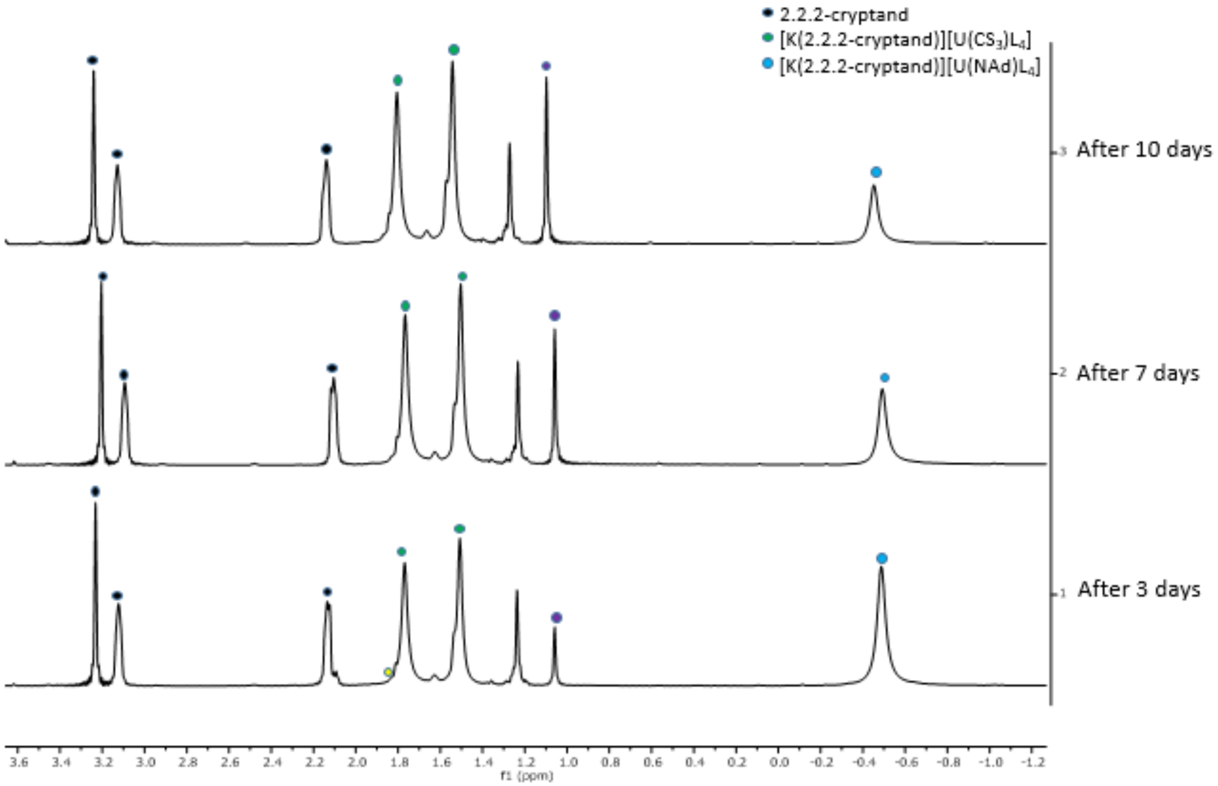

Fig. S18 ^{1}H NMR spectra (400 MHz, d_8-toluene, 298 K) over time of the crude reaction mixture after the addition of $^{13}CS_2$ (1 eq.) to a suspension of **5** (1 eq.), affording **7**.

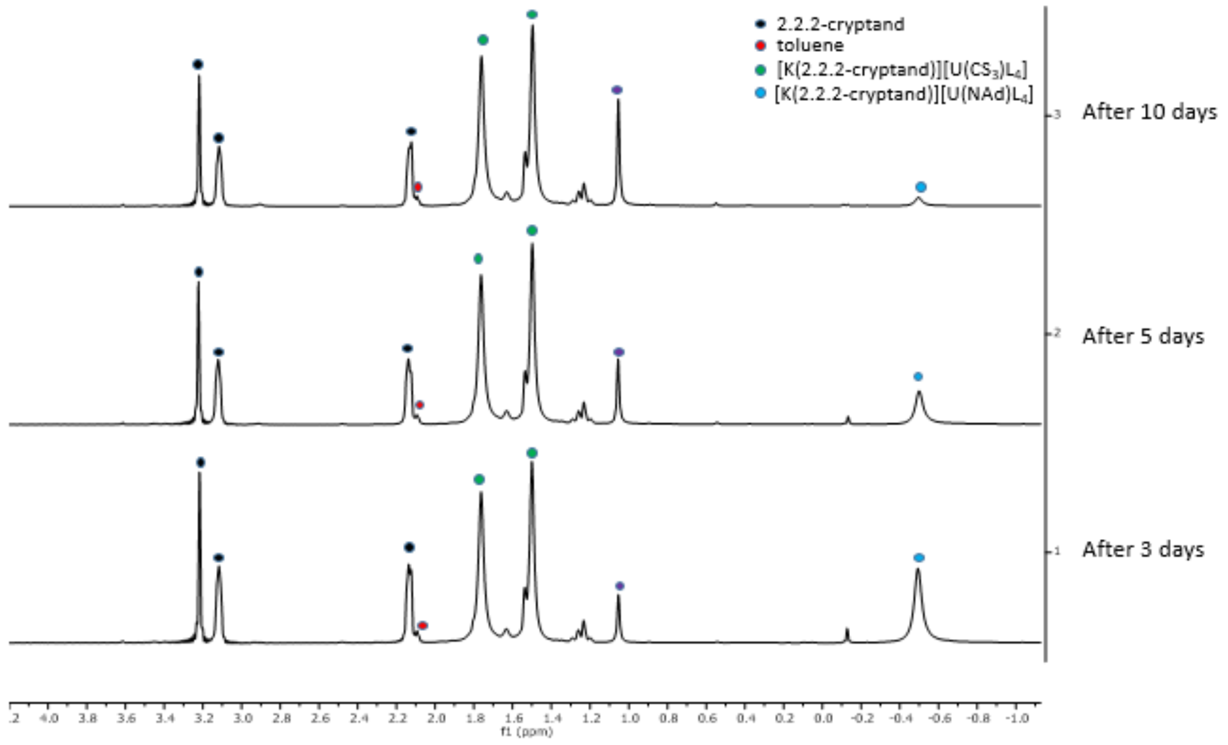

Fig. S19 ^{1}H NMR spectra (400 MHz, d_8-toluene, 298 K) over time of the crude reaction mixture after the addition of $^{13}CS_2$ (2 eq.) to a suspension of **5** (1 eq.), affording **7**.

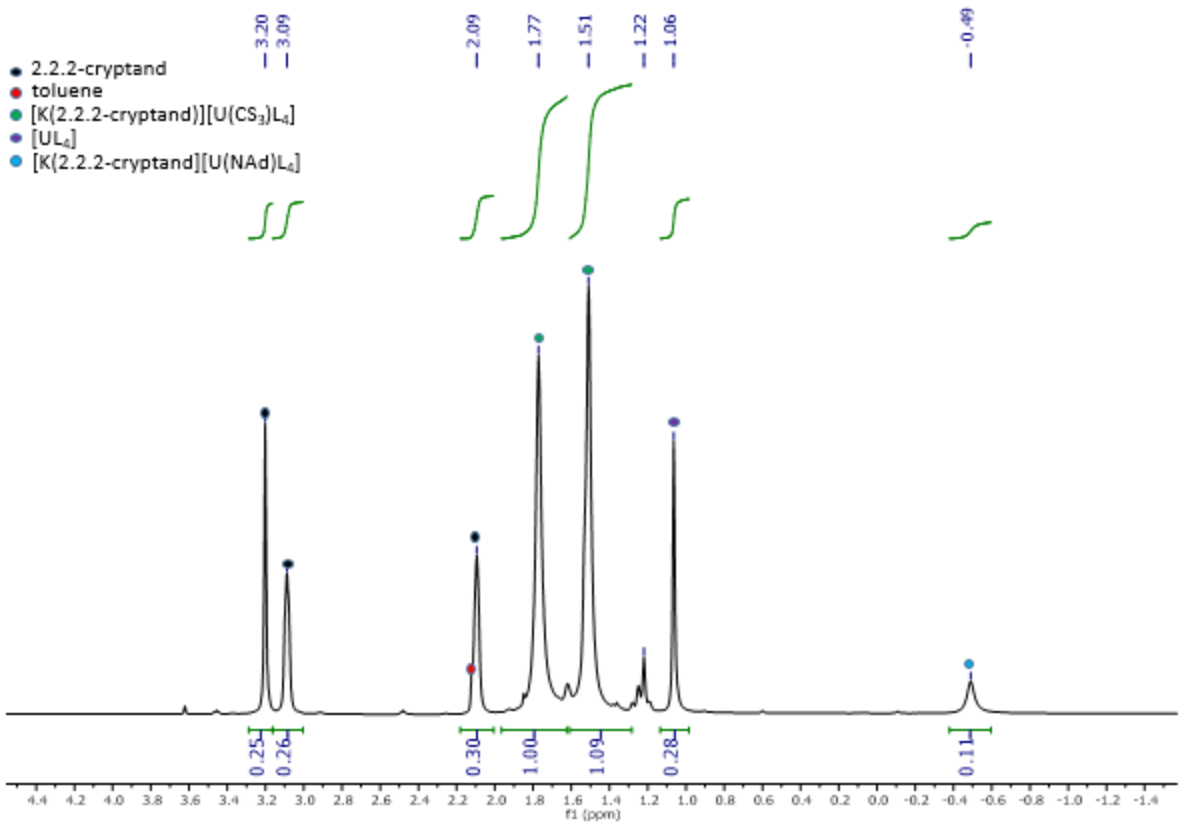

Fig. S20 ^{1}H NMR spectrum (400 MHz, d_8-toluene, 298 K) of the crude reaction mixture ca. 10 days after the addition of $^{13}CS_2$ (2 eq.) to a suspension of **5** (1 eq.), affording **7**. The conversion of **1** into [U{OSi(OtBu)$_3$}$_4$] was determined to be 8 % by quantitative ^{1}H NMR spectroscopy with naphthalene as an internal standard.

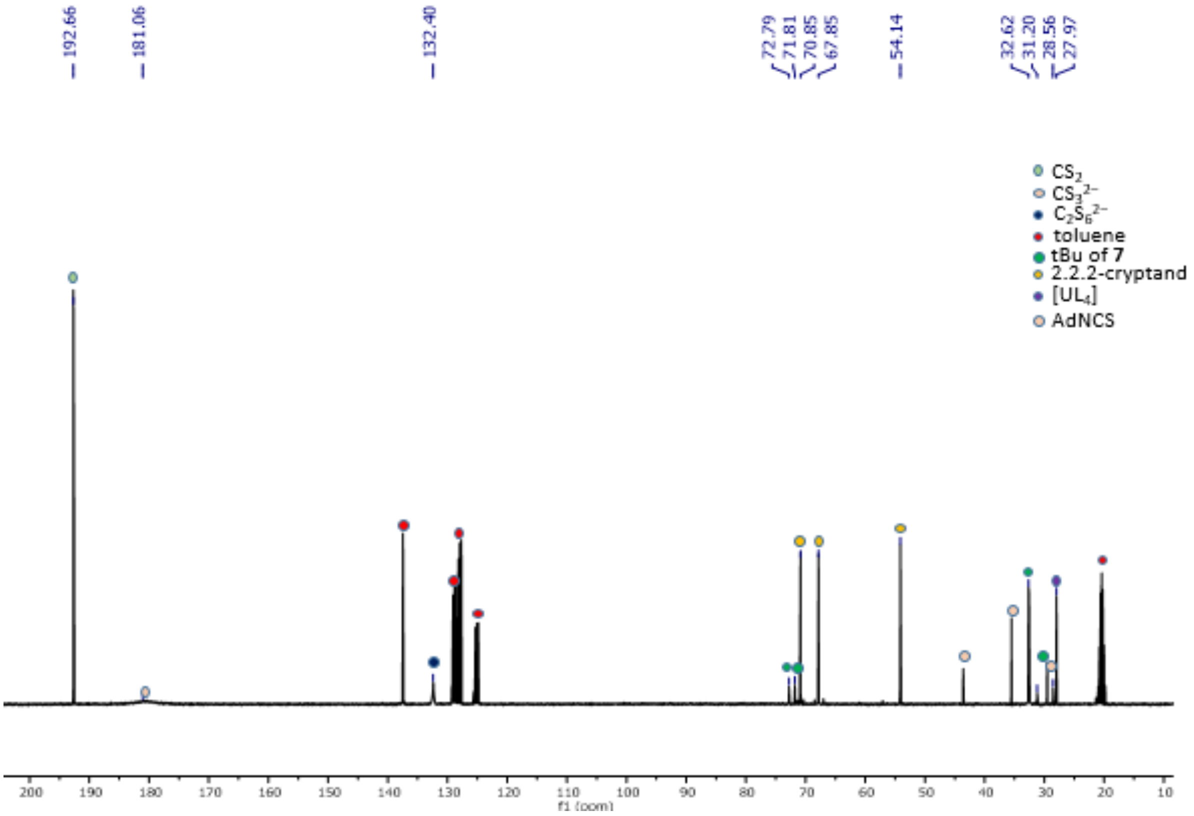

Fig. S21 ^{13}C NMR spectrum (100.6 MHz, d_8-toluene, 298 K) of the crude reaction mixture ca. 10 days after the addition of $^{13}CS_2$ (2 eq.) to a suspension of **5** (1 eq.), affording **7**.

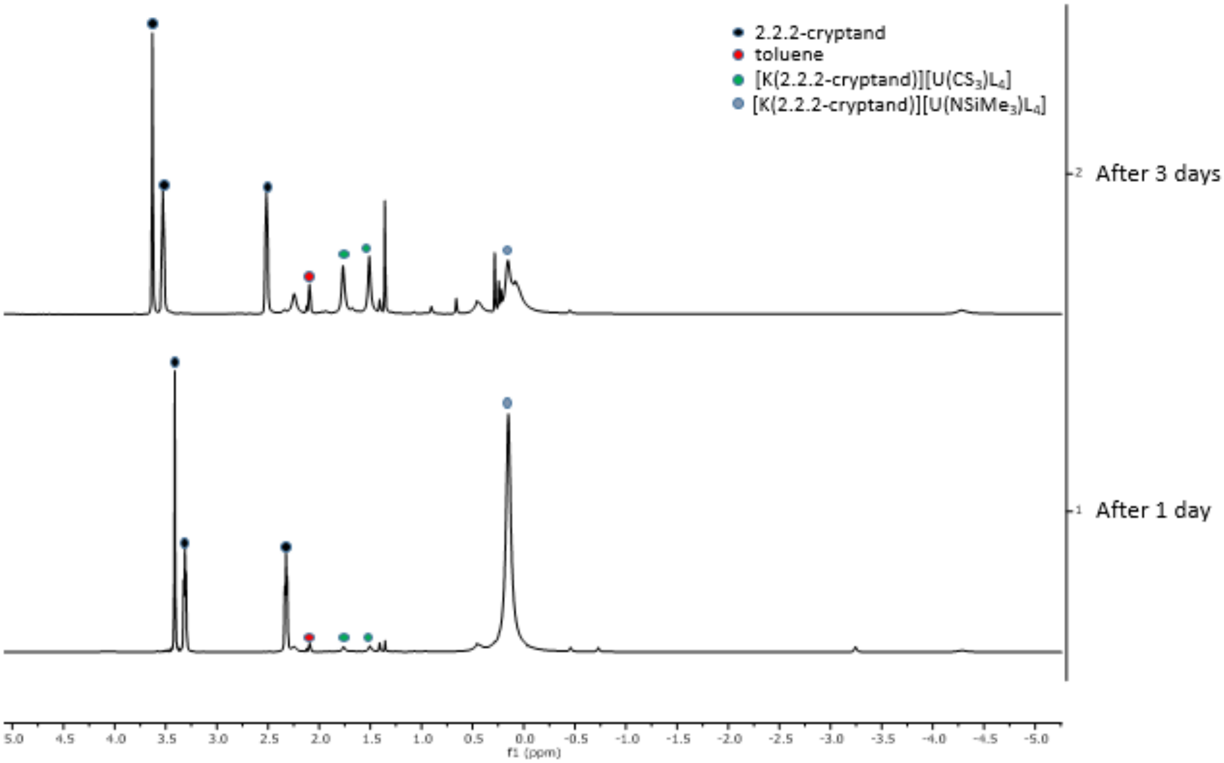

Fig. S22 ^{1}H NMR spectra (400 MHz, d_8-toluene, 298 K) over time of the crude reaction mixture after the addition of $^{13}CS_2$ (1 eq.) to a solution of [K(2.2.2-cryptand)][U(NSiMe$_3$){OSi(OtBu)$_3$}$_4$] (1 eq.), affording **7**.

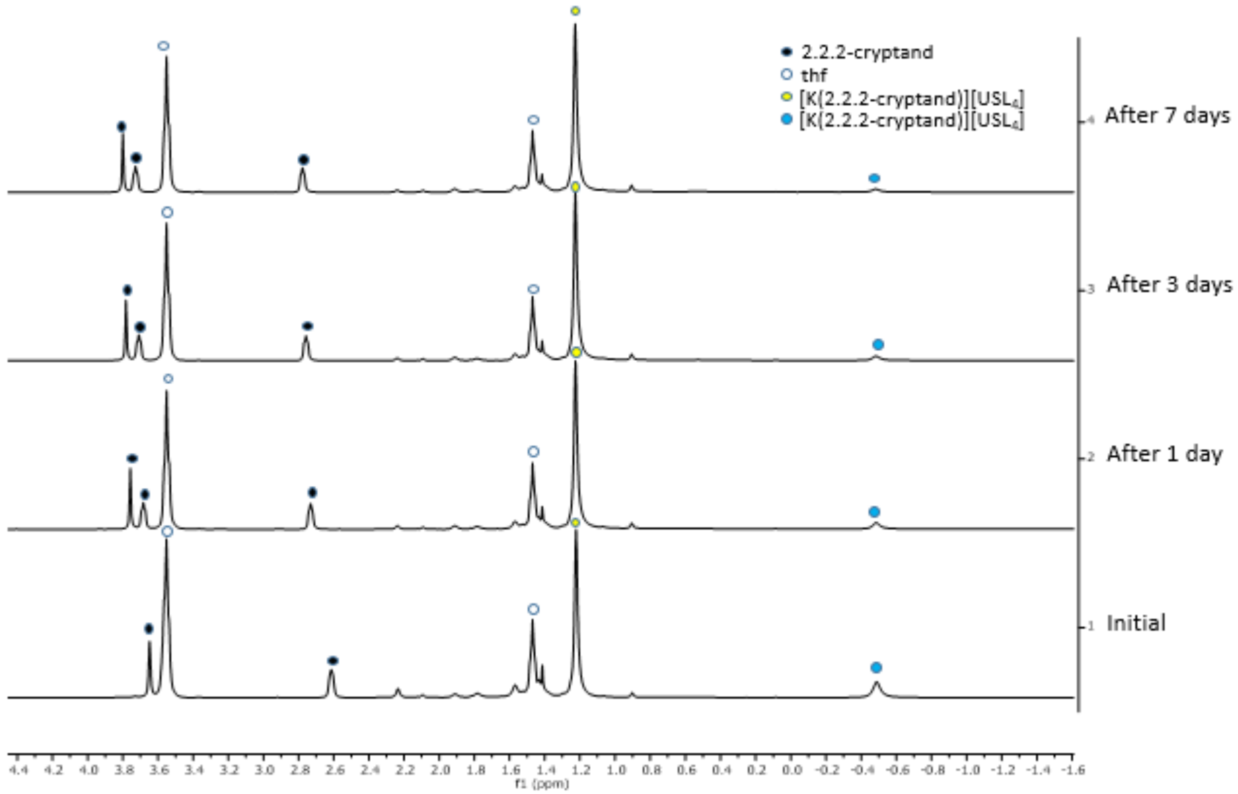

Fig. S23 ^{1}H NMR spectra (400 MHz, d_8-toluene, 298 K) over time of the crude reaction mixture after the addition of H_2S (1.3 eq.) to a suspension of **5** (1 eq.), affording **8**. The reaction mixture was stored at −40 °C between measurements.

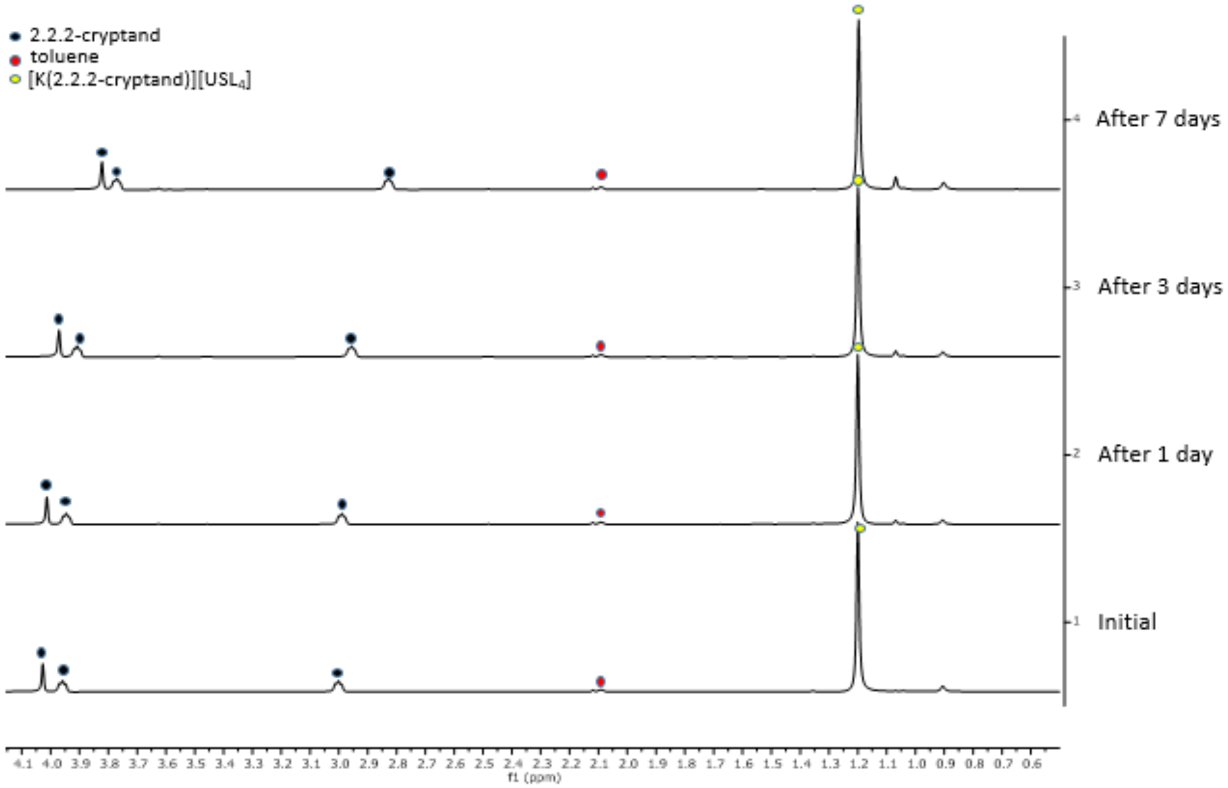

Fig. S24 Monitoring the stability of **8** in d_8-toluene over time by ^{1}H NMR spectroscopy (400 MHz, d_8-toluene, 298 K).

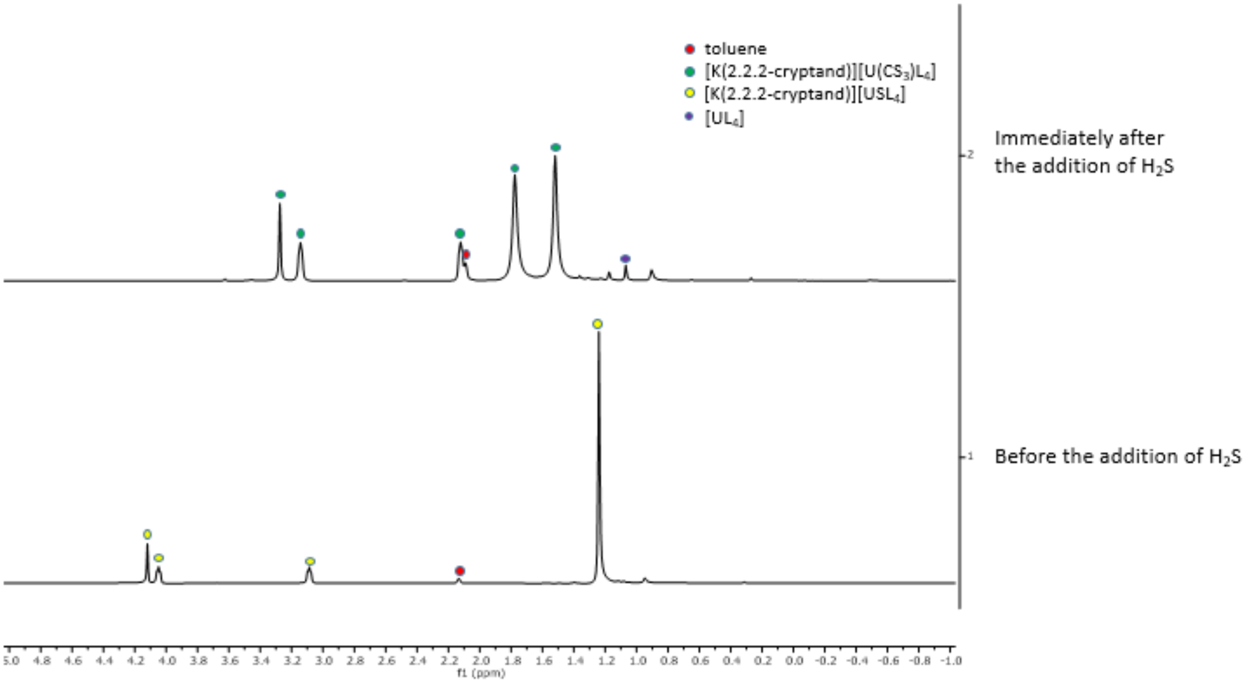

Fig. S25 Conversion of **8** into **7** by reaction with $^{13}CS_2$ (1.3 eq.) as shown by ^{1}H NMR spectroscopy (400 MHz, d_8-toluene, 298 K).

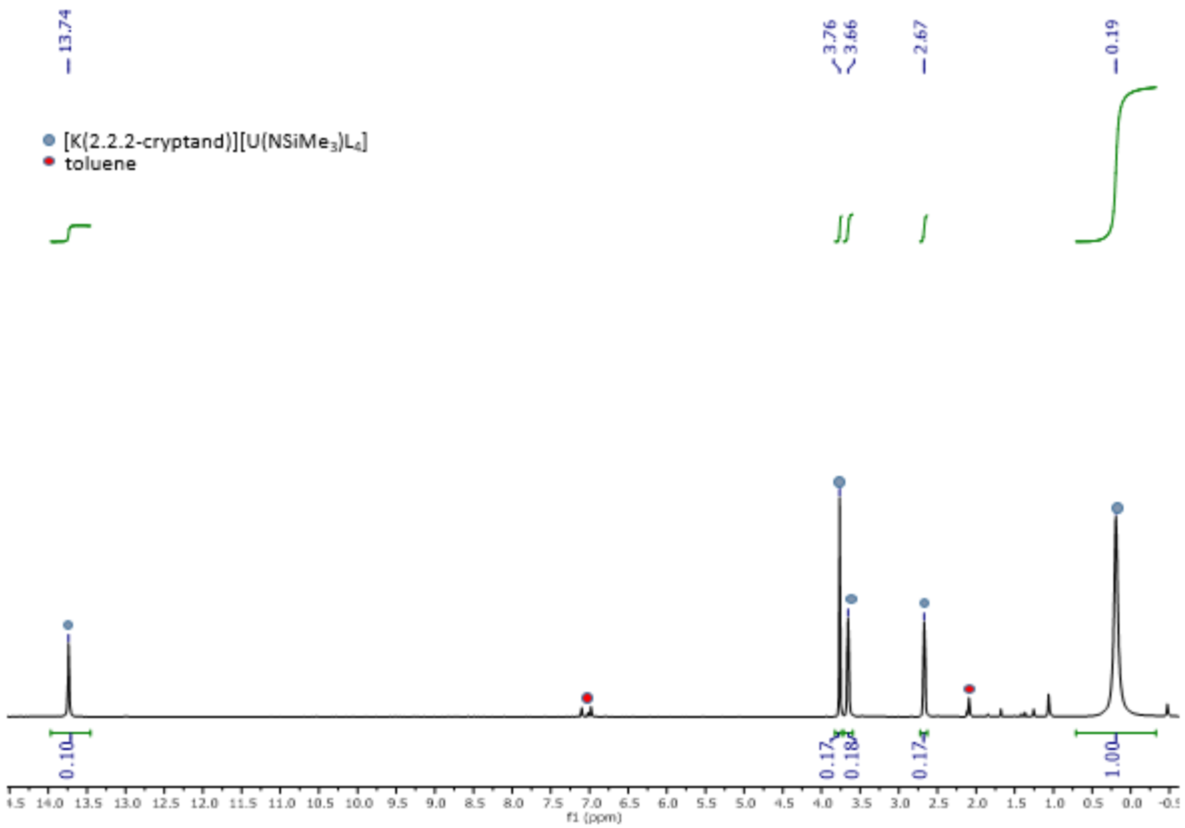

Fig. S26 ^{1}H NMR spectrum (400 MHz, d_8-toluene, 298 K) of [K(2.2.2-cryptand)][U(NSiMe$_3$){OSi(OtBu)$_3$}$_4$].

C) EPR spectra

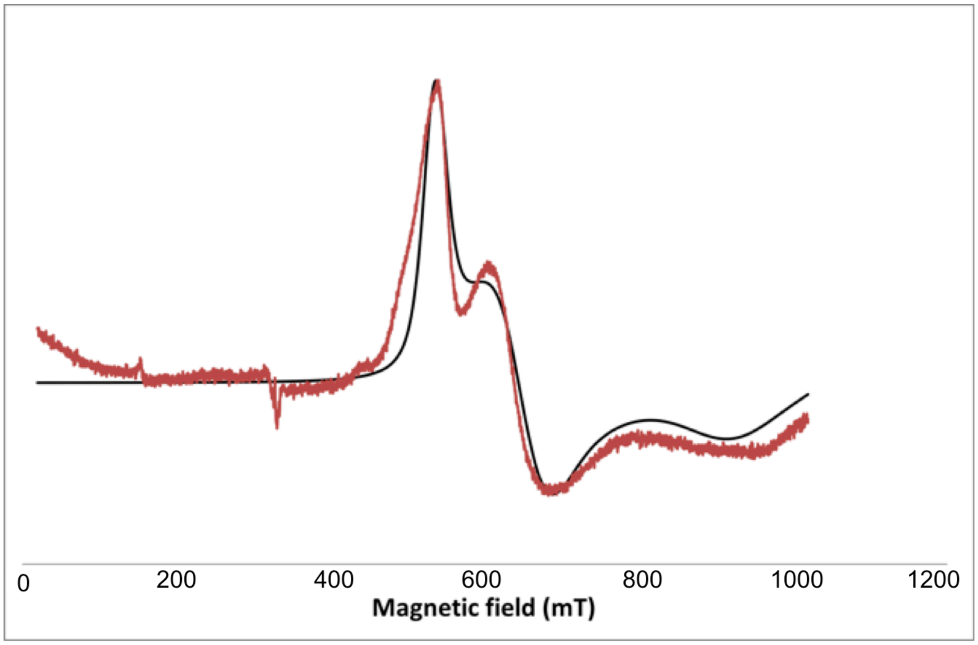

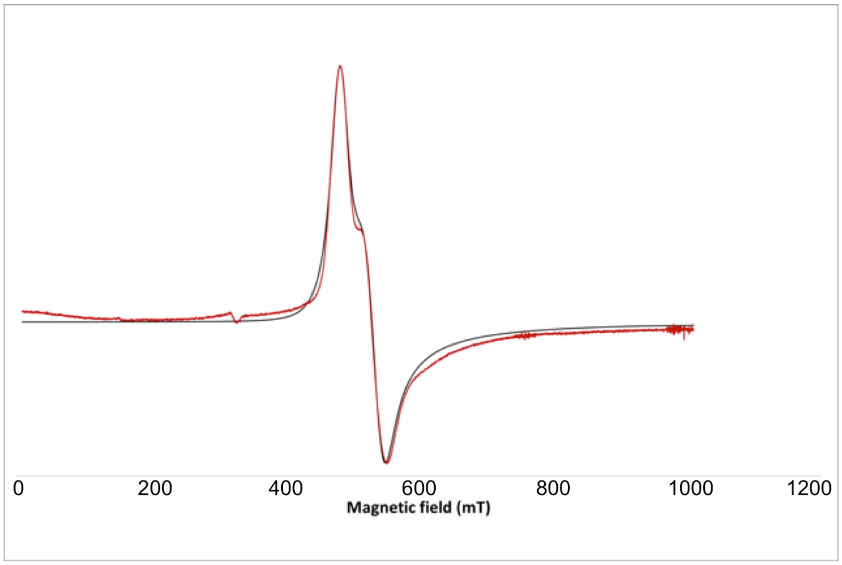

Fig. S27 Band (9.40 GHz) EPR spectrum of crystals of **7** (top) and **8** (bottom) in a 1:1 toluene/hexane glass at 10 K (black lines) and simulated EPR spectra of **7** and **8** (red lines).

D) Electrochemistry

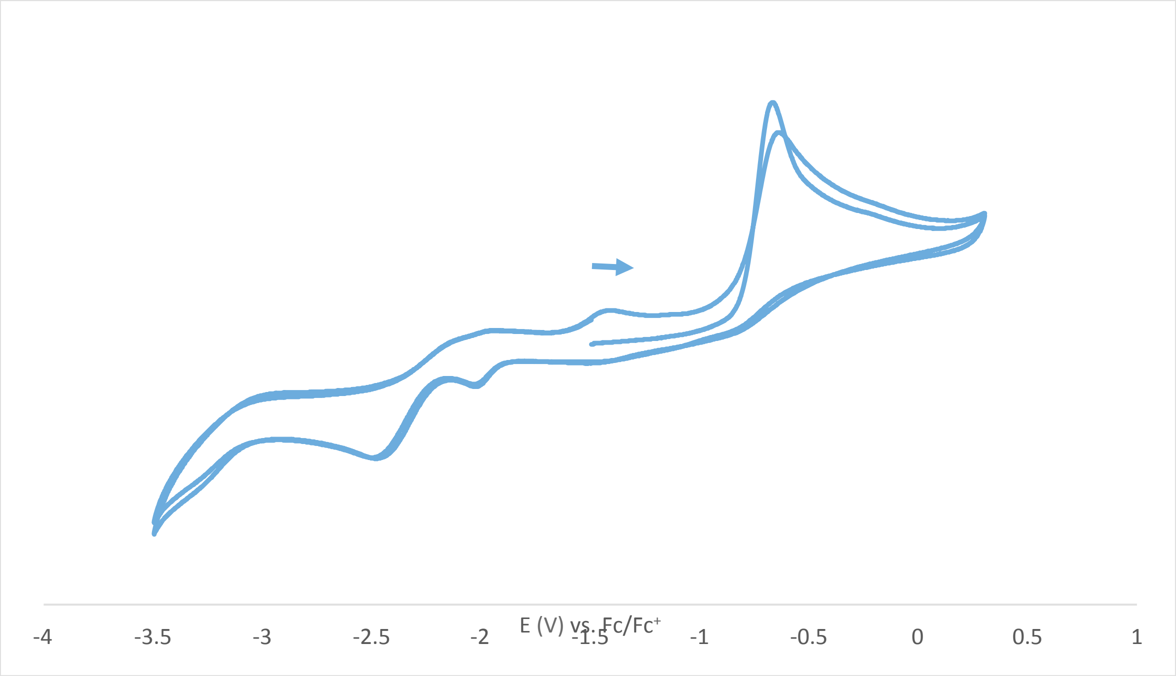

Fig. S28 Cyclic voltammogram trace of [K(2.2.2-cryptand)][US{OSi(OtBu)$_3$}$_4$] (**8**) in an 0.1 M solution of [NBu$_4$][PF$_6$] in thf (Pt electrode, 100 mV/s scan rate).

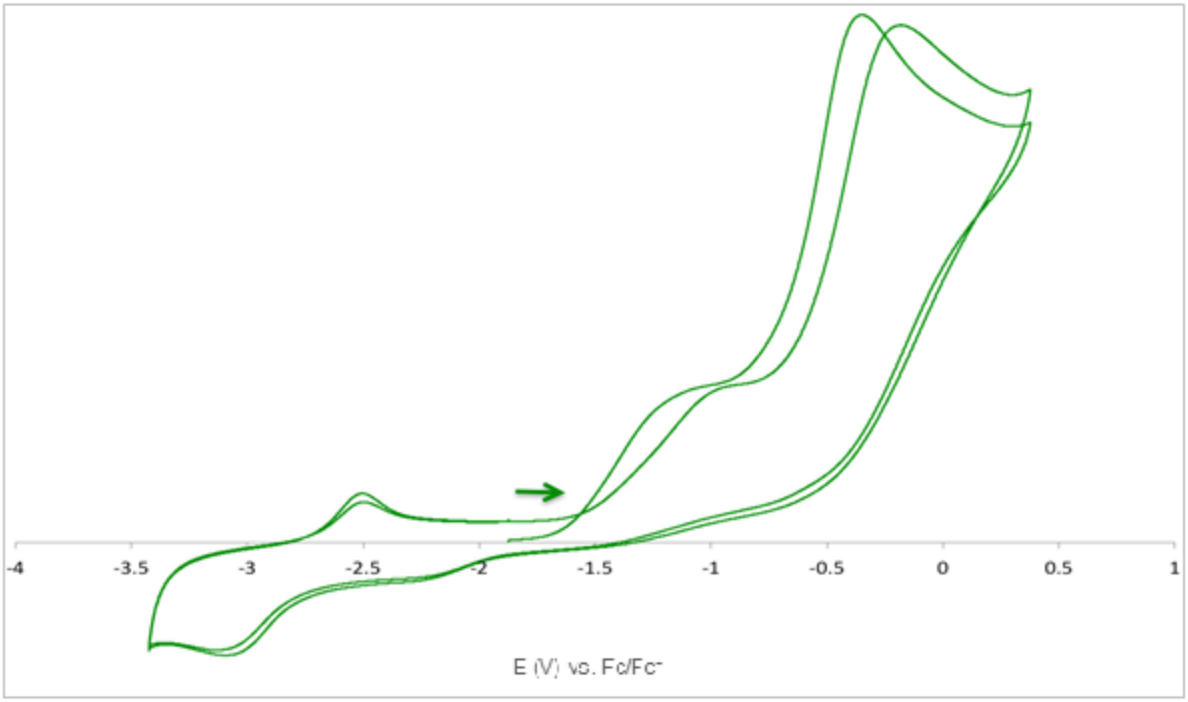

Fig. S29 Cyclic voltammogram trace of [K(2.2.2-cryptand)][US{OSi(OtBu)$_3$}$_4$K] in an 0.1 M solution of [NBu$_4$][PF$_6$] in thf (Pt electrode, 100 mV/s scan rate).

E) IR and Vis/NIR spectra

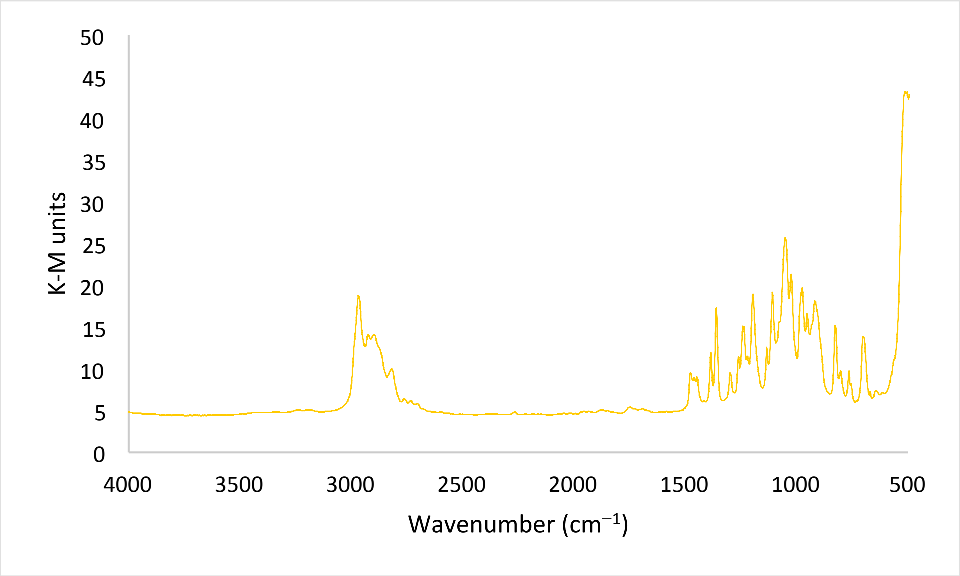

Fig. S30 DRIFT spectrum of complex **8** in KBr (approx. 2 % by weight).

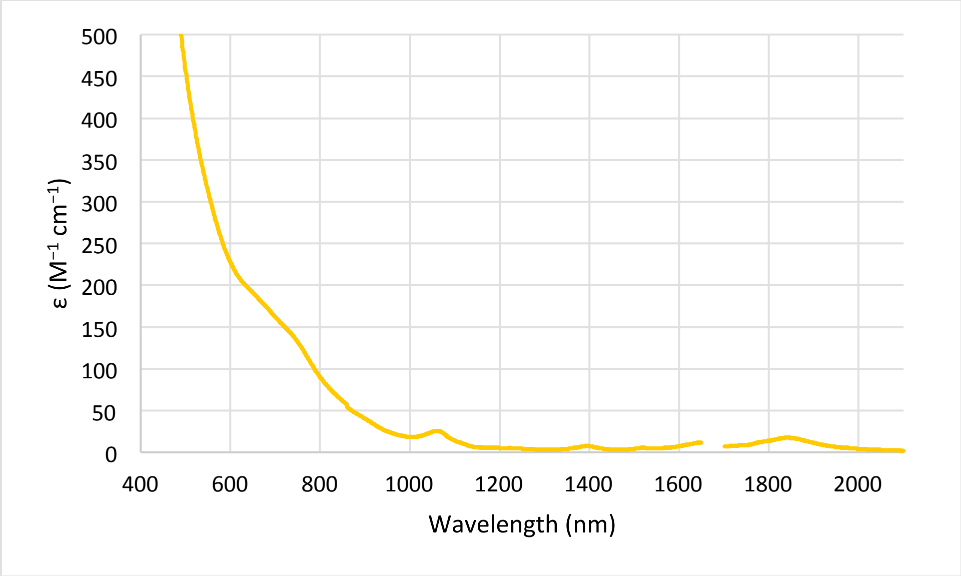

Fig. S31 Absorption spectrum (the region between 1650 and 1700 nm was removed due to heavy noise associated with a change in settings of the instrument) of complex **8** in toluene.

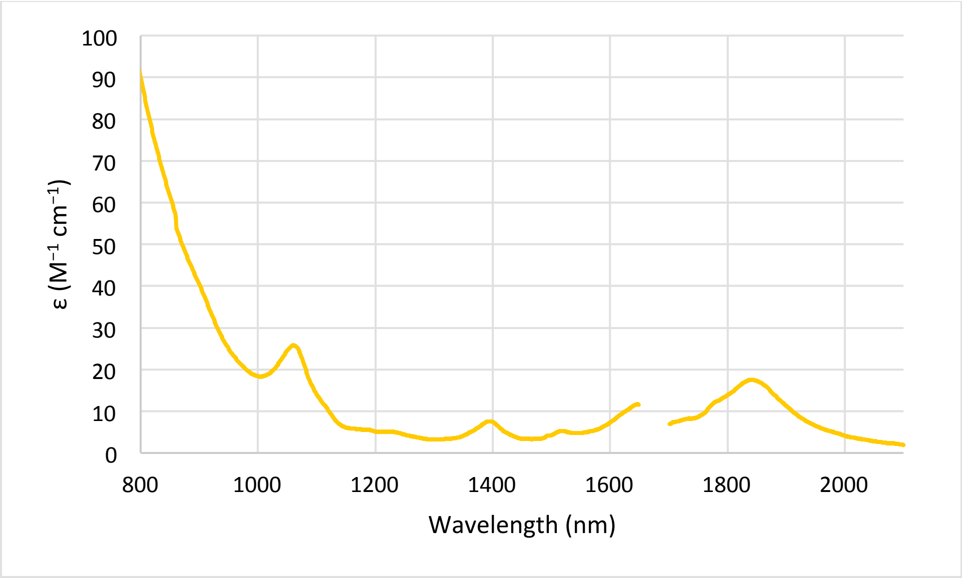

Fig. S32 Zoom (800–2000 nm) of the absorption spectrum of complex **8** in toluene.

F) Crystallographic data

Table S1. Crystal data and structure refinement for **3**.

Empirical formula	$C_{66}H_{144}KN_2O_{22}Si_4U$	
Formula weight	1707.31	
Temperature	100.01(10) K	
Wavelength	1.54184 Å	
Crystal system	Orthorhombic	
Space group	$P2_12_12_1$	
Unit cell dimensions	a = 25.92909(16) Å	α = 90°
	b = 26.18261(18) Å	β = 90°
	c = 26.16679(16) Å	γ = 90°
Volume	17764.41(19) Å^3	
Z	8	
Density (calculated)	1.277 Mg/m^3	
Absorption coefficient	6.590 mm^{-1}	
F(000)	7176	
Crystal size	0.398 x 0.261 x 0.144 mm^3	
Theta range for data collection	3.376 to 75.505°.	
Index ranges	$-32 \leq h \leq 25$, $-31 \leq k \leq 32$, $-32 \leq l \leq 25$	
Reflections collected	134443	
Independent reflections	36333 [$R_{(int)}$ = 0.0346]	

Completeness to theta = 67.684°	100.0 %
Refinement method	Full-matrix least-squares on F^2
Data / restraints / parameters	36333 / 0 / 1802
Goodness-of-fit on F^2	1.096
Final R indices [I>2sigma(I)]	R_1 = 0.0301, wR_2 = 0.0677
R indices (all data)	R_1 = 0.0312, wR_2 = 0.0683
Absolute structure parameter	0.108(3)
Largest diff. peak and hole	1.753 and -1.698 e.Å^{-3}

Table S2. Crystal data and structure refinement for **4**·tol.

Empirical formula	$C_{123}H_{254}K_2N_2O_{32}Si_8U_2$	
Formula weight	3052.25	
Temperature	100.01(10) K	
Wavelength	1.54184 Å	
Crystal system	Orthorhombic	
Space group	*Fdd*2	
Unit cell dimensions	a = 92.6284(8) Å	α = 90°
	b = 24.32104(17) Å	β = 90°
	c = 13.71515(11) Å	γ = 90°
Volume	30897.7(4) Å^3	
Z	8	
Density (calculated)	1.312 Mg/m^3	
Absorption coefficient	7.453 mm^{-1}	
F(000)	12768	
Crystal size	0.330 x 0.240 x 0.202 mm^3	
Theta range for data collection	3.731 to 76.194°.	
Index ranges	$-116 \le h \le 112$, $-30 \le k \le 30$, $-17 \le l \le 9$	
Reflections collected	48571	
Independent reflections	12702 [$R_{(int)}$ = 0.0395]	
Completeness to theta = 67.684°	100.0 %	
Absorption correction	Gaussian	
Max. and min. transmission	0.323 and 0.162	
Refinement method	Full-matrix least-squares on F^2	
Data / restraints / parameters	12702 / 93 / 818	
Goodness-of-fit on F^2	1.034	
Final R indices [I>2sigma(I)]	R_1 = 0.0252, wR_2 = 0.0639	

R indices (all data)	R_1 = 0.0255, wR_2 = 0.0642
Absolute structure parameter	-0.028(3)
Largest diff. peak and hole	1.108 and -1.151 e.Å^{-3}

Table S3. Crystal data and structure refinement for **6**.

Empirical formula	$C_{26}H_{48}K_2O_{12}S_6$	
Formula weight	823.20	
Temperature	100.02(11) K	
Wavelength	1.54184 Å	
Crystal system	Monoclinic	
Space group	$P2_1/c$	
Unit cell dimensions	a = 19.133(2) Å	α = 90°
	b = 9.4570(7) Å	β = 111.465(12)°
	c = 22.775(2) Å	γ = 90°
Volume	3835.1(7) $Å^3$	
Z	4	
Density (calculated)	1.426 Mg/m^3	
Absorption coefficient	5.699 mm^{-1}	
F(000)	1736	
Crystal size	0.134 x 0.109 x 0.084 mm^3	
Theta range for data collection	2.481 to 75.784°.	
Index ranges	$-23 \leq h \leq 24$, $-11 \leq k \leq 11$, $-28 \leq l \leq 28$	
Reflections collected	9167	
Independent reflections	9167 [$R_{(int)}$ = 0.0946]	
Completeness to theta = 67.684°	100.0 %	
Absorption correction	Gaussian	
Max. and min. transmission	0.801 and 0.635	
Refinement method	Full-matrix least-squares on F^2	
Data / restraints / parameters	9167 / 1778 / 598	
Goodness-of-fit on F^2	0.907	
Final R indices [I>2sigma(I)]	R_1 = 0.0829, wR_2 = 0.2090	
R indices (all data)	R_1 = 0.1504, wR_2 = 0.2432	
Largest diff. peak and hole	0.843 and -0.510 e.$Å^{-3}$	

Table S4. Crystal data and structure refinement for **7**·tol.

Identification code	**7**·tol
Empirical formula	$C_{74}H_{152}KN_2O_{22}S_3Si_4U$
Formula weight	1907.65
Temperature	140(2) K
Wavelength	1.54184 Å

Crystal system	Monoclinic	
Space group	$P2_1$	
Unit cell dimensions	a = 13.8270(3) Å	α = 90°.
	b = 24.6484(5) Å	β = 99.042(3)°.
	c = 14.3945(4) Å	γ = 90°.
Volume	4844.9(2) $Å^3$	
Z	2	
Density (calculated)	1.308 Mg/m^3	
Absorption coefficient	6.686 mm^{-1}	
F(000)	2002	
Crystal size	0.24 x 0.17 x 0.12 mm^3	
Theta range for data collection	3.24 to 76.47°.	
Index ranges	$-17 \leq h \leq 15$, $-31 \leq k \leq 21$, $-18 \leq l \leq 17$	
Reflections collected	37530	
Independent reflections	15546 [$R_{(int)}$ = 0.0802]	
Completeness to theta = 76.47°	98.3 %	
Absorption correction	Gaussian	
Max. and min. transmission	0.562 and 0.292	
Refinement method	Full-matrix least-squares on F^2	
Data / restraints / parameters	15546 / 457 / 989	
Goodness-of-fit on F^2	1.052	
Final R indices [I>2sigma(I)]	R_1 = 0.0627, wR_2 = 0.1516	
R indices (all data)	R_1 = 0.0722, wR_2 = 0.1607	
Absolute structure parameter	-0.027(7)	
Largest diff. peak and hole	5.562 and -3.158 e.$Å^{-3}$	

Table S5. Crystal data and structure refinement for **8**·1.5tol.

Empirical formula	$C_{73}H_{152}KN_2O_{22}SSi_4U$	
Formula weight	1831.51	
Temperature	100.01(10) K	
Wavelength	1.54184 Å	
Crystal system	Triclinic	
Space group	$P\bar{1}$	
Unit cell dimensions	a = 14.4445(4) Å	α = 82.352(3)°
	b = 14.4565(6) Å	β = 84.245(3)°
	c = 47.0418(18) Å	γ = 78.583(3)°

Volume	9515.4(6) Å^3
Z	4
Density (calculated)	1.278 Mg/m^3
Absorption coefficient	6.387 mm^{-1}
F(000)	3852
Crystal size	0.264 x 0.111 x 0.052 mm^3
Theta range for data collection	2.852 to 76.401°.
Index ranges	$-17 \leq h \leq 18$, $-18 \leq k \leq 17$, $-59 \leq l \leq 59$
Reflections collected	37472
Independent reflections	37472 [$R_{(int)}$ = 0.0811]
Completeness to theta = 67.684°	98.2 %
Absorption correction	Gaussian
Max. and min. transmission	1.000 and 0.401
Refinement method	Full-matrix least-squares on F^2
Data / restraints / parameters	37472 / 978 / 1973
Goodness-of-fit on F^2	1.093
Final R indices [I>2sigma(I)]	R_1 = 0.0906, wR_2 = 0.2307
R indices (all data)	R_1 = 0.0931, wR_2 = 0.2320
Extinction coefficient	n/a
Largest diff. peak and hole	4.635 and -4.135 e.Å^{-3}

Molecular structure of [K(18c6)]$_2$[C_2S_6] (6)

Yellow crystals of **6** crystallised from toluene in the monoclinic space group $P2_1/c$. The molecular structure is shown in Fig. S30. The complex features two potassium ions encapsulated by two 18c6 molecules. Each potassium ion is bound to two terminal sulfur atoms of the [C_2S_6]$^{2-}$ dianion. Analysis of the bond lengths shows that the binding of the two sulfur atoms to each eight-coordinate potassium ion is uneven (K1–S1 = 3.248(3) Å, K1–S2 = 3.439(3) Å; K2–S5 = 3.222(3) Å, K2–S6 = 3.504(3) Å), while the C–S bond lengths indicate delocalisation of the negative charge over the two sulfur atoms bound to each potassium ion (C1–S1 = 1.664(8) Å, C1–S2 = 1.670(9) Å, C1–S3 = 1.793(9) Å). The S–S bond length is 2.046(3) Å, and this, along with the C–S bond lengths, is consistent with the structural parameters reported for [PPh_4]$_2$[C_2S_6]. [1]

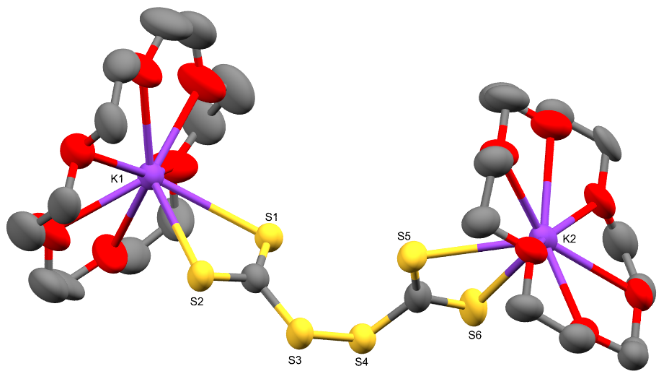

Fig. S30 Molecular structure of [K(18c6)]$_2$[C_2S_6] (**6**) shown with 50% probability thermal ellipsoids. Only one conformation of a disordered 18c6 molecule is shown and hydrogen atoms have been omitted for clarity. Selected bond lengths (Å): K1–S1 = 3.248(3), K1–S2 = 3.439(3), K2–S5 = 3.222(3), K2–S6 = 3.504(3), C1–S1 = 1.664(8), C1–S2 = 1.670(9), C1–S3 = 1.793(9), C2–S4 = 1.784(9), C2–S5 = 1.660(10), C2–S6 = 1.671(9), S3–S4 = 2.046(3).

G) Computational data

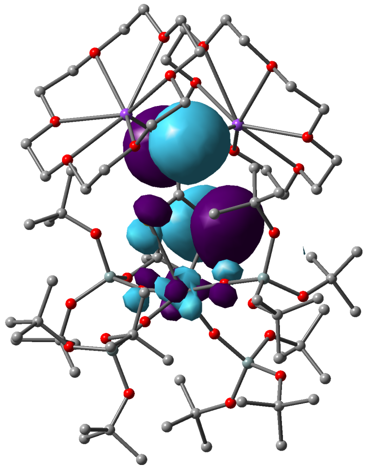	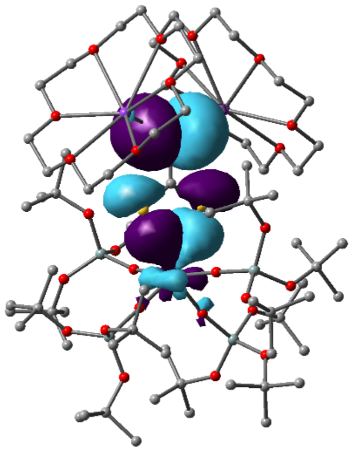
α-HOMO orbital	α-HOMO-1 orbital
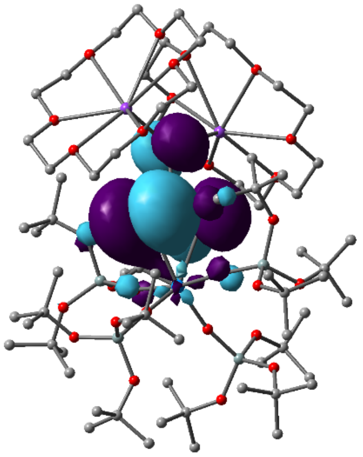	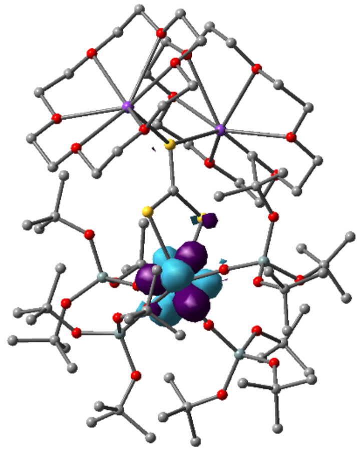
α-HOMO-2 orbital	α-HOMO-3 orbital (orbital f)
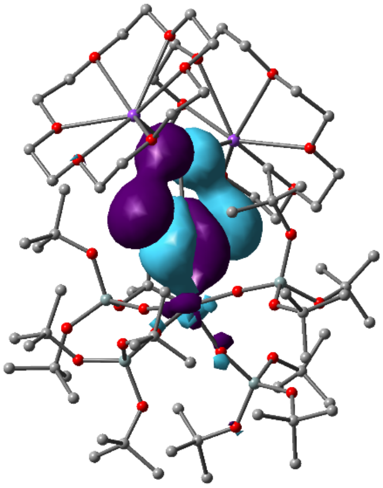	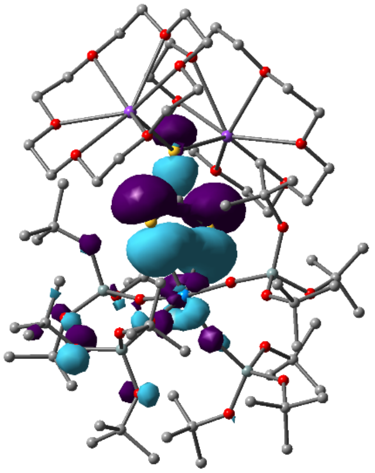
α-HOMO-4 orbital	α-HOMO-10 orbital

Fig. S31 Molecular orbitals of the putative $U^{V}K_2$ complex

Cartesian coordinates of all optimized structures

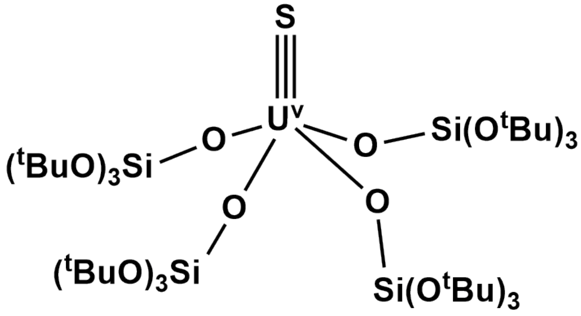

178
Ufive_tetrasiloxide_S
U 8.046663 3.837096 5.317165
S 10.127684 4.690298 6.213101
Si 4.707058 2.730971 4.032370
Si 8.631012 1.226829 7.139287
Si 6.800427 7.312743 5.712817
Si 9.519127 4.139380 1.881671
O 4.583588 1.183252 3.397705
O 3.416057 2.844662 5.090101
O 4.474170 3.904373 2.875787
O 6.163878 3.027723 4.738866
O 10.034894 0.714115 7.876193
O 7.554541 -0.015082 7.457810
O 7.901249 2.604327 7.816625
O 8.714213 1.741572 5.596184
O 5.769326 7.892635 6.912337
O 8.158635 8.269173 5.858027
O 6.179389 7.609531 4.191031
O 7.031856 5.690349 5.813636
O 9.627966 2.657426 1.102879
O 11.106412 4.645474 2.005450
O 8.616499 5.143371 0.893895
O 8.708022 4.052742 3.307810
C 3.726645 8.637014 7.859747
C 4.638378 6.349748 8.389836
C 3.691352 6.826573 6.107696
C 4.466313 7.411681 7.295927
C 11.709029 2.332836 8.595643
C 11.850911 1.273396 6.309986
C 12.188810 -0.130284 8.375606
C 11.438223 1.068673 7.770660
C 8.723261 -1.803882 6.224677
C 6.407096 -2.072540 7.173648

C 8.381559 -1.915094 8.727966
C 7.790272 -1.439160 7.391181
C 5.960964 2.684815 9.246479
C 7.882648 4.310740 9.492587
C 8.155501 1.903379 10.177926
C 7.481738 2.865085 9.188154
C 8.768157 7.661281 8.173135
C 10.348470 8.813115 6.599756
C 8.324669 10.050762 7.477688
C 8.882302 8.687188 7.039139
C 5.766147 10.039960 4.371034
C 7.480494 9.083337 2.777448
C 5.029994 8.656351 2.403736
C 6.126071 8.851793 3.463217
C 11.845588 1.952578 0.409692
C 11.035045 1.485351 2.755841
C 9.964725 0.324284 0.795833
C 10.624485 1.623949 1.284379
C 12.716516 5.523545 3.544594
C 12.402354 6.511989 1.248481
C 10.638647 6.851032 3.020761
C 11.691610 5.889951 2.461961
C 7.487516 3.981869 -0.914332
C 9.771995 4.987774 -1.280850
C 7.786372 6.481959 -0.871080
C 8.435764 5.129375 -0.534627
C 6.166424 1.445056 1.524542
C 6.571589 -0.202200 3.393518
C 4.662587 -0.557701 1.790818
C 5.511230 0.491046 2.529569
C 3.929982 0.873523 6.490216
C 1.757967 1.089723 5.230911
C 2.261715 2.643856 7.152377
C 2.863214 1.851367 5.980790
C 2.440268 3.005199 1.782680
C 2.435421 5.202399 3.023886
C 3.745055 4.961271 0.886838
C 3.268869 4.245002 2.159567
H 3.630631 9.398449 7.077733
H 4.298995 9.059022 8.693874
H 2.728051 8.351931 8.212382
H 5.192284 5.500667 7.980235
H 3.660815 6.003552 8.747589
H 5.203282 6.770188 9.229823
H 2.687199 6.528012 6.432727
H 4.202345 5.945315 5.706714

H 3.606132 7.572262 5.311641
H 11.404706 2.171577 9.636566
H 12.780258 2.568808 8.572010
H 11.157280 3.177987 8.168095
H 11.323930 2.133997 5.887934
H 12.931369 1.457523 6.258938
H 11.613105 0.378777 5.723632
H 11.972954 -1.034936 7.795360
H 13.268952 0.056821 8.359732
H 11.866066 -0.285866 9.411787
H 9.717290 -1.377013 6.390907
H 8.812719 -2.894738 6.146674
H 8.316795 -1.409374 5.288214
H 5.985496 -1.715131 6.229374
H 6.488963 -3.165948 7.145937
H 5.737735 -1.779261 7.990069
H 7.696082 -1.656566 9.543875
H 8.522632 -3.003273 8.713335
H 9.342330 -1.421663 8.897533
H 5.707975 1.649362 8.999271
H 5.591197 2.922372 10.251686
H 5.479352 3.351288 8.524605
H 7.424576 4.987942 8.766333
H 7.553537 4.588366 10.501642
H 8.968969 4.417317 9.415944
H 9.242297 1.994537 10.119580
H 7.826239 2.154695 11.193936
H 7.872607 0.869670 9.956414
H 9.120367 6.688590 7.811432
H 9.390714 7.977491 9.020152
H 7.727745 7.581030 8.504479
H 10.422657 9.513126 5.759082
H 10.963610 9.179447 7.431099
H 10.710109 7.828966 6.283756
H 7.264471 9.942939 7.729020
H 8.873329 10.420538 8.353059
H 8.429335 10.773860 6.659800
H 6.546473 10.188246 5.122352
H 5.670387 10.950056 3.765112
H 4.816647 9.847177 4.881761
H 7.712006 8.222385 2.141863
H 7.446726 9.992247 2.162570
H 8.260076 9.179585 3.538230
H 4.066275 8.478571 2.894463
H 4.953686 9.543961 1.763832
H 5.275224 7.784315 1.789655

H 12.262478 2.912303 0.726800
H 12.605145 1.167205 0.510968
H 11.540705 2.019602 -0.641665
H 10.157326 1.313539 3.386194
H 11.726549 0.640488 2.867604
H 11.533828 2.398298 3.095894
H 9.631308 0.447461 -0.241496
H 10.680237 -0.505391 0.849050
H 9.098649 0.092847 1.423630
H 13.442061 4.807880 3.138457
H 13.247824 6.422903 3.880864
H 12.194905 5.077553 4.398429
H 11.667177 6.758846 0.475922
H 12.929370 7.426164 1.547864
H 13.124336 5.797238 0.836356
H 10.166815 6.417066 3.908311
H 11.117326 7.794744 3.309355
H 9.873871 7.056304 2.264539
H 6.547025 4.096301 -0.366818
H 7.283988 3.997380 -1.992880
H 7.944552 3.027697 -0.637633
H 10.247980 4.040611 -1.012208
H 9.596123 5.010087 -2.363900
H 10.440787 5.811222 -1.012228
H 8.458455 7.296581 -0.578398
H 7.579785 6.550476 -1.946180
H 6.849124 6.582285 -0.313758
H 6.799138 2.180164 2.031302
H 6.798345 0.877672 0.832375
H 5.397571 1.975110 0.954315
H 6.081311 -0.890977 4.090851
H 7.261953 -0.770113 2.757113
H 7.138200 0.535451 3.969955
H 3.891470 -0.056871 1.194835
H 5.295943 -1.160574 1.129222
H 4.174747 -1.214375 2.520636
H 4.766929 1.418361 6.934058
H 3.493203 0.210185 7.247174
H 4.310888 0.271434 5.661491
H 2.198284 0.569774 4.374057
H 1.281931 0.358544 5.896201
H 0.998802 1.795774 4.874081
H 1.525565 3.360856 6.770940
H 1.772229 1.965041 7.861440
H 3.055087 3.194575 7.667788
H 2.093222 2.491093 2.683448

H 1.570394 3.315092 1.189794
H 3.046563 2.310567 1.193258
H 3.043022 6.076152 3.278007
H 1.540218 5.526870 2.477838
H 2.142686 4.695247 3.948013
H 4.360296 4.281129 0.288432
H 2.887261 5.296647 0.290908
H 4.355930 5.824336 1.167071

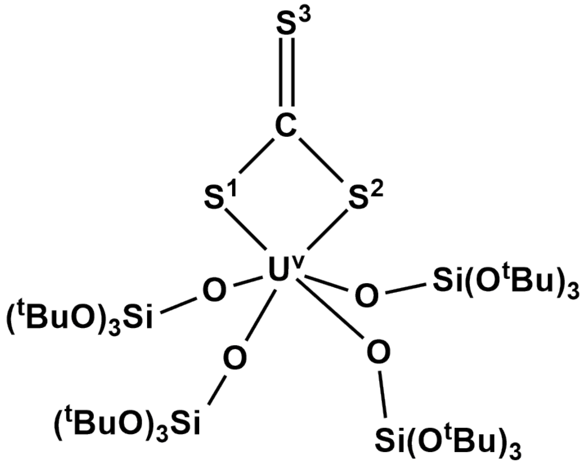

181
Ufive_tetrasiloxide_CS3
U -4.378342 1.977383 -0.662349
S -3.390475 -0.381330 -1.596735
S -3.720677 -2.813241 0.129652
S -4.721037 -0.141365 1.068286
Si -3.444419 3.538614 -3.947351
Si -5.805853 4.800513 1.352776
Si -7.452478 0.410136 -2.025040

Si -1.324025 2.562785 1.447728
O -6.813693 -1.110078 -2.156860
O -8.007275 0.810005 -3.551693
O -3.920347 5.132671 -3.816937
O -1.859915 3.369733 -4.448066
O -4.602888 5.734618 2.023376
O -6.497991 3.977096 2.625841
O -1.788924 3.431848 2.794288
O -4.332523 2.864743 -5.188735
O -3.724610 2.889060 -2.452244
O -6.865776 5.900271 0.669483
O -5.278086 3.630213 0.311463
O -8.800967 0.459766 -1.040000
O -6.250729 1.423067 -1.471037
O -2.554410 2.313754 0.357464
O -0.232195 3.518061 0.615389
O -0.649828 1.175181 2.072992
C -8.435222 1.045529 1.294704
C -8.433727 -1.369751 0.584287
C -6.436263 -2.059938 -4.342728
C -8.697925 -2.297026 -3.243920
C -7.957155 3.180145 -4.065014
C -10.096411 2.085752 -3.263460
C -5.357907 1.236510 -6.579244
C -3.069396 2.125137 -7.133012
C -3.049963 5.975314 -5.978058
C -3.981035 7.474173 -4.184293
C -0.743127 4.848051 -2.825964
C -0.254559 2.375766 -2.934289
C -6.962978 6.035824 -1.742620
C -8.770428 6.959745 -0.256906
C -4.619923 8.060146 1.283274
C -2.628545 6.993090 2.385027
C -6.753141 5.853375 4.228984
C -7.095356 3.437226 4.856008
C 0.790999 5.676121 0.801053
C 1.285453 3.944117 2.551906
C -1.199641 2.354349 4.912834
C -3.432757 1.915730 3.813171
C -0.754399 -1.213748 2.086178
C -6.691725 -3.480624 -2.281688
C -7.179697 -2.220390 -3.009861
C -9.153570 1.499366 -5.509596
C -8.799854 1.900256 -4.066693
C -5.515797 5.931593 -5.459433
C -4.108005 6.104641 -4.870222

C 0.420592 3.879398 -4.840709
C -0.623616 3.623672 -3.741519
C -3.262171 6.311080 0.049520
C -3.795124 6.769524 1.412011
C -8.736799 4.588023 3.334260
C -7.259958 4.485661 3.744698
C -2.908349 4.189525 4.749142
C -2.328571 2.949081 4.055134
C -3.393704 0.583280 -5.155818
C -4.015733 1.700683 -5.999948
C -8.499545 4.469019 -0.487806
C -7.765458 5.812704 -0.455699
C -3.944245 -1.175157 -0.126794
C -10.557389 -0.024579 0.466950
C -9.026848 0.020933 0.321758
C 2.125136 3.631437 0.198229
C 0.975519 4.173315 1.064055
C 1.442142 0.000000 2.066335
C 0.000000 0.000000 1.529978
C 0.000000 0.000000 0.000000
H -10.831960 -0.328368 1.483997
H -10.976595 0.966614 0.260506
H -10.973487 -0.742021 -0.249413
H -8.650136 0.748974 2.328711
H -7.351445 1.106388 1.168287
H -8.865754 2.032911 1.105757
H -7.344372 -1.356042 0.485247
H -8.688142 -1.683354 1.604147
H -8.847038 -2.095168 -0.123413
H -6.863287 -4.365795 -2.906768
H -5.624507 -3.387015 -2.057180
H -7.229118 -3.596612 -1.334601
H -5.361420 -1.992951 -4.145655
H -6.634399 -2.920207 -4.994503
H -6.768164 -1.143100 -4.838357
H -9.043448 -1.400788 -3.766677
H -8.927393 -3.182821 -3.849074
H -9.221296 -2.376698 -2.285608
H -9.717151 0.559408 -5.502075
H -9.762756 2.281228 -5.978635
H -8.238033 1.360691 -6.091870
H -7.026664 3.016717 -4.616619
H -8.516310 4.003229 -4.527579
H -7.696107 3.456270 -3.040234
H -10.694870 2.884780 -3.718819
H -10.673912 1.155195 -3.268398

H -9.872594 2.341039 -2.225532
H -3.225428 -0.303395 -5.779030
H -2.435842 0.908949 -4.739211
H -4.063892 0.311620 -4.334869
H -5.816306 2.053191 -7.149543
H -5.206795 0.375501 -7.241022
H -6.026157 0.949345 -5.761925
H -2.858741 1.267516 -7.783830
H -3.538840 2.915976 -7.730502
H -2.134325 2.499331 -6.707152
H -5.703684 6.694711 -6.225100
H -5.604383 4.935288 -5.901954
H -6.259165 6.030505 -4.662233
H -3.135567 5.004489 -6.474507
H -3.199868 6.770170 -6.719204
H -2.044774 6.065975 -5.554944
H -4.145700 8.280528 -4.908993
H -4.721979 7.550889 -3.382222
H -2.981456 7.576983 -3.747586
H 1.405173 4.058931 -4.393198
H 0.479562 3.006751 -5.501062
H 0.128447 4.753171 -5.434105
H -1.457338 4.654187 -2.020099
H 0.230660 5.059664 -2.370954
H -1.071204 5.720999 -3.399443
H -0.225322 1.502227 -3.595060
H 0.726989 2.506275 -2.465850
H -0.992080 2.206642 -2.145970
H -9.054493 4.323273 0.444128
H -9.206941 4.455619 -1.324343
H -7.793451 3.642236 -0.612127
H -6.443866 6.999631 -1.686766
H -6.219167 5.244765 -1.876029
H -7.631845 6.041167 -2.611020
H -9.482618 6.989279 -1.089790
H -9.318181 6.811868 0.680535
H -8.234545 7.914210 -0.205291
H -2.597248 7.076823 -0.367745
H -2.701327 5.379402 0.168170
H -4.080641 6.139799 -0.657141
H -4.987973 8.358345 2.271981
H -3.991392 8.863254 0.878787
H -5.475926 7.894199 0.624735
H -2.080250 6.054569 2.508783
H -1.957777 7.769273 1.997262
H -3.016982 7.308109 3.360424

H -9.348573 4.886578 4.194405
H -8.845364 5.333017 2.540131
H -9.083028 3.616811 2.965999
H -7.328342 6.159067 5.112030
H -5.693325 5.790965 4.487754
H -6.877942 6.606277 3.443814
H -6.039983 3.361848 5.136307
H -7.686798 3.718242 5.735750
H -7.430478 2.461234 4.488964
H 3.059103 4.157737 0.429870
H 2.260696 2.561731 0.386880
H 1.883130 3.772793 -0.860077
H 1.702304 6.224501 1.069894
H 0.568199 5.840317 -0.257696
H -0.045742 6.051019 1.397068
H 1.364122 2.874371 2.767397
H 2.236457 4.431812 2.799967
H 0.488276 4.365230 3.169266
H -3.671352 4.639916 4.108143
H -3.349845 3.905123 5.712233
H -2.112979 4.923819 4.924101
H -0.423875 3.110011 5.082287
H -1.604287 2.039356 5.882861
H -0.760055 1.494405 4.402179
H -3.852515 1.588665 4.772691
H -4.234179 2.359571 3.213994
H -3.036617 1.040434 3.289398
H 1.951703 -0.923173 1.765348
H 1.995897 0.855931 1.666536
H 1.430116 0.062299 3.160567
H -0.286976 -2.142589 1.739248
H -0.734190 -1.187585 3.182202
H -1.793942 -1.202833 1.748535
H 0.504954 0.895922 -0.377144
H 0.525656 -0.889020 -0.368697
H -1.024090 -0.023916 -0.384464

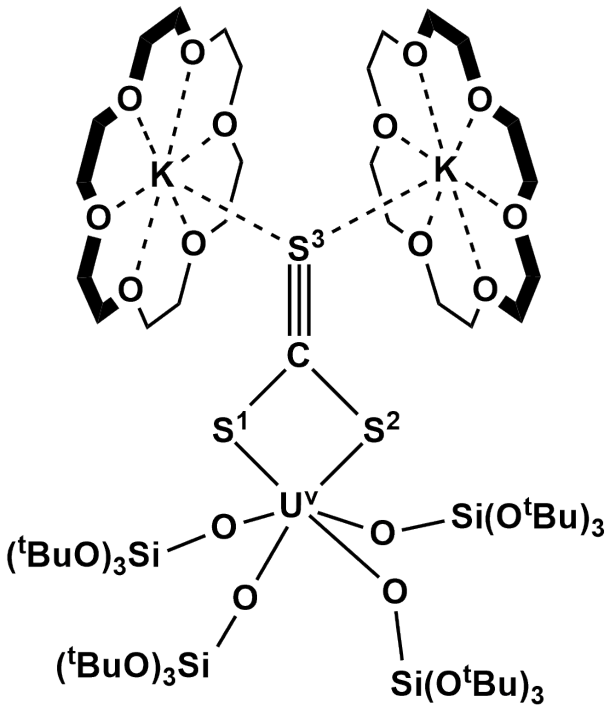

267
Ufive_tetrasiloxide_CS2_2Kcrownether
U -4.320207 1.964001 -0.803863
K -5.805906 -3.243712 3.322727
K -1.685927 -4.368054 -1.465762
S -4.838821 -0.149146 1.121477
S -3.968699 -2.959809 0.519177
S -3.393406 -0.718376 -1.420351
Si -7.436520 0.500983 -2.309412
Si -3.222770 3.326715 -4.109151
Si -5.707488 4.901565 1.056845
Si -1.288883 2.550159 1.372442
O -6.769254 -1.234495 5.179523
O -8.658992 -2.637855 3.635168
O -7.976148 -5.249887 2.879167
O -5.306272 -6.181453 2.852619
O -3.559026 -4.699956 4.547784
O -4.065181 -2.001446 5.134329
O -1.249363 -3.377870 -4.092696
O 0.769467 -3.023237 -2.144970
O 0.961821 -5.035604 -0.204232
O -1.291799 -6.572665 0.570918
O -3.243848 -6.874849 -1.474094
O -3.502835 -4.967768 -3.513221
O -6.868376 -1.052856 -2.475650
O -7.878445 0.990343 -3.833340

O -3.582747 4.932424 -3.917718
O -1.670159 2.997134 -4.603065
O -4.454411 5.763095 1.710341
O -6.488376 4.129119 2.307369
O -8.814909 0.600292 -1.381831
O -6.167227 1.358735 -1.642724
O -4.196927 2.710836 -5.304652
O -3.539378 2.665581 -2.598502
O -6.647303 6.011372 0.260325
O -5.201062 3.629064 0.083149
O -1.737908 3.514745 2.648851
O -2.545736 2.235089 0.316563
O -0.176308 3.385627 0.461937
O -0.687060 1.162891 2.083235
C -4.546010 -0.972120 5.995179
C -5.707656 -0.284461 5.299156
C -7.959208 -0.619392 4.692570
C -9.039529 -1.685031 4.627706
C -9.638749 -3.661581 3.492306
C -9.165036 -4.615856 2.408408
C -7.535620 -6.278024 1.998180
C -6.431647 -7.044633 2.708813
C -4.294772 -6.782756 3.657579
C -3.130574 -5.811974 3.769040
C -2.505447 -3.750260 4.716510
C -2.972940 -2.703428 5.713958
C -3.228342 -4.488584 -4.828006
C -2.524703 -3.149662 -4.695731
C -0.511842 -2.159965 -3.969046
C 0.880360 -2.511387 -3.474117
C 2.029039 -3.476200 -1.659408
C 1.848064 -3.917768 -0.216706
C 0.814404 -5.570155 1.107240
C 0.036080 -6.870495 0.996991
C -2.041023 -7.769190 0.370509
C -3.415603 -7.397303 -0.161455
C -4.498717 -6.594324 -2.095119
C -4.225175 -6.192272 -3.535029
C -6.888778 6.165020 -2.128561
C -8.605708 7.092531 -0.542195
C -4.468339 8.109169 0.989492
C -2.484300 7.035871 2.087439
C -8.723366 1.280423 0.940640
C -8.440727 -1.146112 0.321632
C -6.745498 -2.010130 -4.692983
C -8.918819 -2.089316 -3.423898

C -5.342427 1.221565 -6.754359
C -3.001984 1.964526 -7.298401
C -2.652235 5.777881 -6.057466
C -3.486428 7.288233 -4.226968
C -7.907628 3.333000 -4.436176
C -10.015010 2.223977 -3.582576
C -6.933794 -3.417627 -2.615066
C -7.386328 -2.119929 -3.302567
C -9.073313 1.568826 -5.807322
C -8.721074 2.036945 -4.386092
C -0.443075 4.434409 -3.015084
C 0.002123 1.961377 -3.211990
C -5.115836 5.887533 -5.546438
C -3.703282 5.953443 -4.952264
C 0.596257 3.502858 -5.114534
C -0.390031 3.236189 -3.967926
C 0.835539 5.567790 0.485481
C 1.349389 3.969922 2.353009
C -1.149056 2.582397 4.836272
C -3.410804 2.142278 3.797412
C -6.712021 6.114936 3.793960
C -7.036202 3.748711 4.590168
C -3.114450 6.361544 -0.252436
C -3.646386 6.818255 1.110287
C -8.709400 4.789740 3.029537
C -7.224998 4.718222 3.413729
C -10.658420 -0.004611 -0.025192
C -9.131559 0.174446 -0.038753
C -3.424404 0.381300 -5.369373
C -3.967678 1.568455 -6.173164
C -8.362891 4.599672 -0.794749
C -7.625227 5.939975 -0.804203
C -2.801403 4.456142 4.558431
C -2.268900 3.151800 3.951360
C -4.066163 -1.311481 0.073731
C -0.754911 -1.217726 2.114513
C 2.180461 3.493995 0.024894
C 1.034598 4.091733 0.855452
C 1.428068 0.021558 2.141710
C 0.001268 0.002549 1.569462
C 0.032516 -0.012698 0.039696
H -5.183226 -6.067411 -4.063880
H -3.639934 -6.981809 -4.031907
H -5.140002 -7.490979 -2.086794
H -5.012606 -5.772607 -1.574951
H -3.891324 -6.658912 0.502281

H -4.040895 -8.306012 -0.189309
H -1.537745 -8.420564 -0.360513
H -2.149519 -8.319935 1.319639
H 0.024585 -7.375151 1.977054
H 0.536584 -7.524633 0.266665
H 1.800012 -5.783654 1.552171
H 0.277029 -4.863685 1.758858
H 1.432508 -3.086616 0.372288
H 2.828247 -4.197480 0.202363
H 2.392755 -4.323528 -2.260926
H 2.774914 -2.665919 -1.695694
H 1.512263 -1.609746 -3.484835
H 1.320516 -3.269545 -4.140054
H -0.427207 -1.660549 -4.947247
H -1.006917 -1.478778 -3.262085
H -3.130072 -2.472419 -4.076095
H -2.400203 -2.706263 -5.695836
H -2.583458 -5.197076 -5.370658
H -4.165568 -4.348710 -5.388880
H -8.360540 -6.971609 1.767840
H -7.153517 -5.853187 1.057046
H -6.161988 -7.937872 2.121854
H -6.801947 -7.365064 3.694643
H -4.683230 -7.003855 4.663643
H -3.943359 -7.722245 3.199561
H -2.812893 -5.495800 2.764190
H -2.287133 -6.322203 4.264381
H -1.603186 -4.244041 5.113446
H -2.264977 -3.268992 3.757313
H -2.140905 -2.014726 5.932503
H -3.282380 -3.199072 6.647646
H -4.889549 -1.398498 6.950627
H -3.752830 -0.234358 6.191669
H -5.391294 0.068795 4.307426
H -6.030022 0.579149 5.902066
H -8.283539 0.185121 5.372154
H -7.798943 -0.193381 3.690875
H -10.000635 -1.216374 4.362723
H -9.135317 -2.171125 5.610533
H -9.761701 -4.213761 4.436609
H -10.609776 -3.231472 3.198979
H -8.970490 -4.055604 1.481962
H -9.951171 -5.364001 2.217654
H -10.998877 -0.303186 0.973345
H -11.143376 0.937381 -0.302176
H -10.946730 -0.774226 -0.749695

H -9.009447 1.002479 1.962389
H -7.643074 1.445600 0.906586
H -9.227652 2.213460 0.673711
H -7.352816 -1.038107 0.273556
H -8.717687 -1.438172 1.341181
H -8.748367 -1.932868 -0.374243
H -7.254909 -4.286634 -3.202878
H -5.843359 -3.415505 -2.515847
H -7.371262 -3.475399 -1.612616
H -5.655411 -2.013134 -4.587713
H -7.054765 -2.853132 -5.323378
H -7.053066 -1.073200 -5.164609
H -9.238761 -1.162702 -3.908577
H -9.254592 -2.941459 -4.027073
H -9.383585 -2.150551 -2.435231
H -9.618414 0.619280 -5.761679
H -9.703958 2.316756 -6.301228
H -8.162149 1.429292 -6.395655
H -6.968285 3.164629 -4.970370
H -8.479004 4.120370 -4.942330
H -7.670611 3.664298 -3.421393
H -10.641449 2.970388 -4.085575
H -10.563625 1.278704 -3.522689
H -9.798882 2.558736 -2.566078
H -3.326637 -0.488167 -6.029962
H -2.442457 0.627511 -4.952742
H -4.115691 0.136684 -4.555596
H -5.739422 2.083961 -7.301731
H -5.258522 0.371487 -7.441535
H -6.028034 0.967194 -5.940782
H -2.870233 1.123355 -7.989562
H -3.415781 2.812435 -7.855902
H -2.033289 2.247349 -6.877865
H -5.249806 6.687644 -6.284010
H -5.269575 4.917984 -6.028720
H -5.856268 6.009174 -4.750466
H -2.793511 4.826177 -6.578418
H -2.751856 6.593473 -6.783351
H -1.643394 5.805621 -5.634977
H -3.598752 8.121900 -4.929311
H -4.220665 7.394956 -3.422464
H -2.481537 7.318161 -3.791612
H 1.608105 3.645601 -4.718440
H 0.598461 2.653458 -5.806908
H 0.297598 4.401957 -5.663325
H -1.104010 4.227672 -2.167785

H 0.561022 4.627622 -2.623157
H -0.796133 5.325457 -3.541841
H 0.023730 1.115111 -3.908840
H 0.992534 2.079614 -2.758718
H -0.724551 1.762215 -2.419533
H -8.856095 4.453657 0.170543
H -9.122818 4.590911 -1.583474
H -7.667994 3.771331 -0.963982
H -6.365895 7.127400 -2.096517
H -6.153515 5.371956 -2.298439
H -7.601437 6.176157 -2.960809
H -9.361837 7.134290 -1.334281
H -9.105788 6.944720 0.421043
H -8.062126 8.043004 -0.514543
H -2.466124 7.136489 -0.677517
H -2.532899 5.441854 -0.132410
H -3.935611 6.177340 -0.953339
H -4.849047 8.396941 1.976014
H -3.829161 8.915405 0.610341
H -5.312458 7.962493 0.312594
H -1.944986 6.094732 2.226346
H -1.804223 7.800449 1.694380
H -2.871406 7.369700 3.056529
H -9.303439 5.130528 3.885690
H -8.847178 5.494519 2.204051
H -9.061104 3.800206 2.719010
H -7.283433 6.485025 4.653593
H -5.651571 6.075567 4.054983
H -6.843430 6.811246 2.958966
H -5.974644 3.687257 4.849378
H -7.602664 4.096377 5.461669
H -7.391722 2.753403 4.300625
H 3.110243 4.043130 0.212232
H 2.329954 2.442496 0.291689
H 1.935383 3.557397 -1.039949
H 1.748954 6.135713 0.698245
H 0.597950 5.657038 -0.578519
H 0.010465 5.987639 1.066860
H 1.457994 2.920138 2.642440
H 2.288759 4.494188 2.564622
H 0.546209 4.416782 2.943577
H -3.556053 4.885446 3.893408
H -3.239584 4.257588 5.544122
H -1.982697 5.174813 4.674516
H -0.346537 3.320299 4.941915
H -1.548481 2.357933 5.833197

H -0.742793 1.672834 4.387249
H -3.823372 1.915482 4.788407
H -4.205173 2.570393 3.177531
H -3.049688 1.215129 3.339418
H 1.961397 -0.895000 1.860599
H 1.980985 0.881211 1.751949
H 1.389406 0.091158 3.234463
H -0.256789 -2.143261 1.798912
H -0.769827 -1.172482 3.209888
H -1.783177 -1.223210 1.742207
H 0.539628 0.881968 -0.334961
H 0.561015 -0.903356 -0.319244
H -0.988013 -0.042253 -0.353737

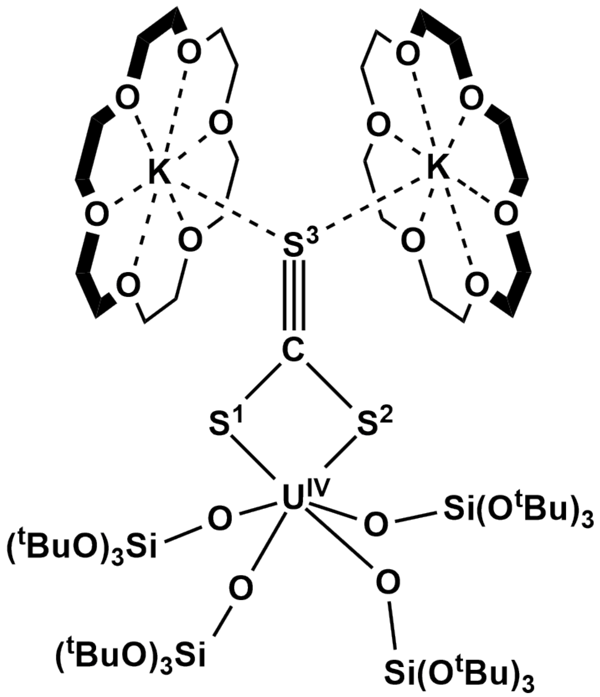

267
Ufour_tetrasiloxide_carbS_K2_scpop.log
U 3.725709 2.876791 -0.756753
K -0.253679 6.489388 3.076574
K -2.641225 3.637426 -2.097557
S -0.969389 5.079575 0.265520
S 0.829873 3.471520 -1.547753
S 1.842230 4.404119 1.101569
Si 4.163380 6.398672 -2.290180
Si 4.771182 1.269174 -4.045990
Si 6.792003 2.582344 1.485600
Si 2.533311 -0.235986 1.240893
O -0.999475 9.466506 2.777092
O -3.067832 7.536112 2.542769
O -2.775366 5.188431 4.148082
O -0.196498 4.381917 4.894726
O 1.826989 6.332986 5.109415
O 1.577074 8.682539 3.565297
O -2.241996 5.632345 -4.006898
O -1.849535 2.954474 -4.776777
O -2.733806 0.905784 -3.036282
O -4.802250 1.546409 -1.249388
O -5.044694 4.194657 -0.260087
O -4.207091 6.229833 -2.087284
O 8.349615 2.971941 1.001114
O 6.901202 1.028290 2.085601

O 4.755919 7.653425 -1.336383
O 2.573762 6.694025 -2.730727
O 4.903892 2.385591 -5.287637
O 6.330137 0.804112 -3.672702
O 5.011137 6.586952 -3.726719
O 4.220692 4.939351 -1.568959
O 3.875875 0.029013 -4.740823
O 4.102247 1.832286 -2.661571
O 2.822827 -1.612731 0.331001
O 3.432010 -0.424900 2.643956
O 6.348448 3.516009 2.806996
O 5.638111 2.816506 0.346838
O 2.932850 1.045630 0.318013
O 0.941917 -0.219357 1.807295
C -2.066036 9.581986 1.842291
C -3.296500 8.937026 2.461346
C -4.154080 6.861260 3.166879
C -3.828257 5.376482 3.210033
C -2.403581 3.811942 4.232503
C -1.353132 3.678695 5.322584
C 0.879326 4.260537 5.819751
C 2.095681 4.931271 5.206153
C 2.956616 7.028163 4.586410
C 2.611542 8.506464 4.531837
C 1.217734 10.051174 3.422974
C 0.168127 10.150813 2.327572
C -2.922402 6.878985 -3.986489
C -1.798892 5.277191 -5.313557
C -0.975214 4.007559 -5.188170
C -1.133710 1.728930 -4.608871
C -2.149502 0.639883 -4.311774
C -3.781503 -0.009557 -2.744319
C -4.241359 0.237585 -1.316879
C -5.275213 1.838800 0.060325
C -5.998646 3.174756 0.010278
C -5.670991 5.467706 -0.375268
C -4.615990 6.496192 -0.751360
C -3.254438 7.194567 -2.537374
C 3.225937 -2.195335 -5.246726
C 4.259491 -1.681452 -3.006278
C 2.512328 2.881520 -5.583917
C 4.328357 4.174009 -6.737487
C 7.873417 5.159378 3.715888
C 8.145785 2.733659 4.339601
C 8.067791 5.102004 -0.189974
C 8.795020 2.994656 -1.379358

C 6.507212 7.132111 -5.487589
C 7.022673 5.241514 -3.903475
C 4.931307 9.467624 0.170153
C 5.136331 7.100752 0.997058
C 6.847228 -0.314411 0.030070
C 8.960342 -0.104042 1.415651
C 10.318057 4.087170 0.297882
C 8.854555 3.793028 -0.071166
C 6.966596 -1.333237 2.320988
C 7.424813 -0.160663 1.441964
C 0.611340 7.943468 -3.160117
C 2.067568 7.133417 -5.050091
C 2.897772 7.983515 0.241256
C 4.414557 8.026434 0.008394
C 2.852167 9.015928 -3.572643
C 2.058706 7.697720 -3.620497
C 8.108121 1.717467 -5.048281
C 6.897683 -0.364744 -5.785289
C 3.878034 1.789147 -7.408726
C 3.895945 2.788448 -6.241256
C 8.361264 -0.422497 -3.740765
C 7.401366 0.438919 -4.575740
C 0.554397 4.329348 -0.064162
C 7.162930 7.645936 -3.128290
C 6.422586 6.645700 -4.030089
C 1.951837 -0.828818 -3.555122
C 3.341330 -1.158193 -4.117584
C 1.819498 -3.689751 -0.309641
C 4.192557 -3.572816 0.516384
C 4.599684 0.041776 4.663532
C 2.120569 -0.388501 4.701068
C 2.287165 -3.268009 2.126198
C 2.772738 -3.011199 0.689712
C 3.033390 1.751280 3.710991
C 3.276742 0.250525 3.911743
C -1.156855 -1.322017 1.493463
C -0.976877 1.184571 1.581252
C 6.127720 4.014672 5.119217
C 7.137188 3.835343 3.973290
C -0.301179 -0.106280 1.093932
C -0.103615 -0.071195 -0.425252
H -2.279076 7.676367 -4.390278
H -3.851167 6.828464 -4.577001
H -3.680150 8.210439 -2.474875
H -2.337570 7.141724 -1.931907
H -3.764377 6.435736 -0.057609

H -5.062907 7.503558 -0.680526
H -6.447076 5.447665 -1.156737
H -6.139470 5.754825 0.581680
H -6.498032 3.355393 0.977367
H -6.762581 3.138621 -0.782281
H -5.981514 1.062971 0.400804
H -4.438534 1.894240 0.773723
H -3.380319 0.146914 -0.638445
H -4.994030 -0.519705 -1.040402
H -4.626023 0.135027 -3.436387
H -3.425615 -1.049622 -2.827266
H -1.645395 -0.339019 -4.303382
H -2.923540 0.644046 -5.095223
H -0.588372 1.470280 -5.530296
H -0.413302 1.811663 -3.782513
H -0.176545 4.159244 -4.447711
H -0.521057 3.768543 -6.162253
H -2.658290 5.103217 -5.980076
H -1.166701 6.074624 -5.734247
H -2.282765 10.642426 1.630262
H -1.817855 9.071893 0.898686
H -4.178011 9.151019 1.833268
H -3.461191 9.365343 3.462538
H -4.304738 7.231749 4.193140
H -5.084697 7.015453 2.594366
H -3.529053 5.033268 2.208276
H -4.727676 4.819972 3.526495
H -3.278029 3.192994 4.495212
H -1.988220 3.466773 3.274659
H -1.123853 2.611524 5.474763
H -1.742014 4.100885 6.263301
H 0.624969 4.746434 6.775108
H 1.110705 3.199625 6.001342
H 2.284461 4.505210 4.210871
H 2.974189 4.751548 5.845503
H 3.834471 6.883838 5.236835
H 3.204087 6.667943 3.576899
H 3.507466 9.079035 4.242888
H 2.272103 8.842467 5.523816
H 0.803657 10.443527 4.364951
H 2.094447 10.654709 3.136819
H 0.557544 9.697129 1.404286
H -0.055962 11.213690 2.137756
H 4.734585 9.833607 1.185536
H 6.010033 9.493722 -0.018950
H 4.432137 10.121193 -0.554276

H 4.919741 7.404514 2.028963
H 4.813205 6.066854 0.850646
H 6.216793 7.155064 0.831064
H 2.516009 6.967446 0.100613
H 2.672053 8.299431 1.266662
H 2.393662 8.651098 -0.465987
H 0.123108 8.677449 -3.814490
H 0.060246 6.997257 -3.177278
H 0.615657 8.318794 -2.130797
H 1.511550 6.189326 -5.066404
H 1.607194 7.843102 -5.749615
H 3.099830 6.937605 -5.352419
H 3.882622 8.842819 -3.894848
H 2.381757 9.750180 -4.238768
H 2.866414 9.412904 -2.553254
H 6.046806 8.123338 -5.572710
H 7.555431 7.195553 -5.802738
H 5.979011 6.435936 -6.145255
H 6.466909 4.537078 -4.529082
H 8.075737 5.248768 -4.211061
H 6.957053 4.898674 -2.867257
H 8.212879 7.704604 -3.441351
H 6.705067 8.636947 -3.213609
H 7.110887 7.336654 -2.082301
H 1.787415 3.249409 -6.320320
H 2.199147 1.893323 -5.232263
H 2.548296 3.572984 -4.735505
H 5.331072 4.109524 -7.175474
H 3.628418 4.542235 -7.497150
H 4.351363 4.871249 -5.894305
H 3.149846 2.105859 -8.165907
H 4.871427 1.749289 -7.870664
H 3.615873 0.795054 -7.036083
H 8.962065 1.464965 -5.689091
H 7.401204 2.338881 -5.605163
H 8.465137 2.279717 -4.179929
H 6.211411 0.239704 -6.385010
H 7.752614 -0.658312 -6.406882
H 6.374043 -1.265617 -5.451152
H 9.232328 -0.712310 -4.340275
H 8.695837 0.144935 -2.866708
H 7.844101 -1.324508 -3.395102
H 2.779958 -3.122986 -4.869113
H 2.599097 -1.795143 -6.052019
H 4.220044 -2.413080 -5.651810
H 4.288949 -0.979697 -2.167580

H 3.874968 -2.636784 -2.633344
H 5.272871 -1.826980 -3.392575
H 1.312388 -0.451889 -4.363236
H 1.494325 -1.724543 -3.119611
H 2.042209 -0.065694 -2.777110
H 8.107098 5.644475 0.759848
H 8.504762 5.728792 -0.975171
H 7.021573 4.898976 -0.438053
H 9.354603 2.059388 -1.263783
H 7.757695 2.756423 -1.633573
H 9.237296 3.573892 -2.198053
H 10.794195 4.687659 -0.485971
H 10.357056 4.636956 1.245111
H 10.866260 3.145714 0.414897
H 7.198440 -1.253047 -0.414705
H 5.754246 -0.332396 0.079459
H 7.155875 0.511841 -0.618018
H 9.340568 -0.002251 2.438669
H 9.358374 -1.027196 0.977053
H 9.294960 0.756452 0.832012
H 5.873956 -1.338194 2.374954
H 7.321938 -2.280142 1.897080
H 7.372734 -1.219142 3.332684
H 8.404651 5.484010 4.619093
H 8.595160 5.021732 2.905722
H 7.151665 5.929808 3.422163
H 8.673230 3.017198 5.259012
H 7.626659 1.784130 4.494148
H 8.876171 2.605826 3.534161
H 5.589675 3.075078 5.280886
H 6.644470 4.303601 6.042234
H 5.407638 4.794382 4.846638
H 1.802087 -4.773948 -0.146502
H 0.805841 -3.293805 -0.183885
H 2.154500 -3.483762 -1.331415
H 4.202792 -4.652407 0.710872
H 4.541197 -3.385079 -0.503982
H 4.870731 -3.074343 1.214533
H 1.289809 -2.844121 2.277157
H 2.249527 -4.349744 2.304566
H 2.972593 -2.803164 2.838698
H 5.418382 0.477095 4.082817
H 4.549289 0.517719 5.651013
H 4.786462 -1.030275 4.794843
H 2.319248 -1.456416 4.846097
H 2.033447 0.089450 5.685544

H 1.186494 -0.273235 4.145280
H 2.949701 2.230936 4.694550
H 3.877089 2.198079 3.176537
H 2.112496 1.928576 3.145088
H -2.156626 -1.248885 1.046639
H -0.679506 -2.243471 1.145136
H -1.253040 -1.364531 2.584433
H -1.962373 1.305896 1.112515
H -1.099833 1.135382 2.670095
H -0.355057 2.047937 1.328220
H 0.430177 -0.967881 -0.754736
H -1.073988 -0.019103 -0.932900
H 0.476188 0.813188 -0.703690

1. A. Muller, E. Krickemeyer, F. Elkatri, D. Rehder, A. Stammler, H. Bogge and F. Hellweg, *Z. Anorg. Allg. Chem.*, **1995**, 621, 1160-1170.
